# Multi-wavelength photo-activation of transmembrane chloride transport in distinct vesicle populations using photocaged indolocarbazole anionophores

**DOI:** 10.1039/d6sc04824b

**Published:** 2026-08-26

**Authors:** Kelly R. Britton, Matthew J. Langton

**Affiliations:** a Chemistry Research Laboratory, Department of Chemistry, University of Oxford 12 Mansfield Road Oxford UK matthew.langton@chem.ox.ac.uk

## Abstract

Synthetic ionophores enable the study of transmembrane ion transport and show promise as therapeutics or for engineering responsive synthetic cells. Incorporation of photolabile protecting groups (photocages) allows for OFF–ON control over transport to spatiotemporally regulate activity; however, systems incorporating two different photocages such that two wavelengths of irradiation are required for activation are unknown. Here, we report a family of photocaged indolocarbazole-based anionophores that enable multi-wavelength photo-activation of chloride transport in lipid bilayer vesicles. Mono- and di-*ortho*-nitrobenzyl photocaged derivatives fully inhibit anion binding and transport until irradiation cleaves the protecting group to restore activity. Introduction of a BODIPY photocage afforded a unique dual-photocaged transporter, in which sequential irradiation at 530 nm and 365 nm triggers stepwise photocage cleavage to regenerate the active anionophore. This strategy also allows for selective activation of anion transport in different vesicles in a mixed population containing different mono-photocaged transporters. These results establish photocaged indolocarbazoles as a versatile platform for photo-responsive ionophores and enable unprecedented multi-wavelength sequential control of transmembrane transport in separate vesicle populations. Beyond fundamental insights into the design of stimuli-responsive transporters, this approach provides new opportunities for spatiotemporal regulation of ion transport in artificial and living cellular systems.

## Abstract 2

Photocaged indolocarbazole chloride transporters are reported that provide spatiotemporal control of transmembrane chloride transport in distinct vesicle populations by multi-wavelength activation.

## Graphical abstract

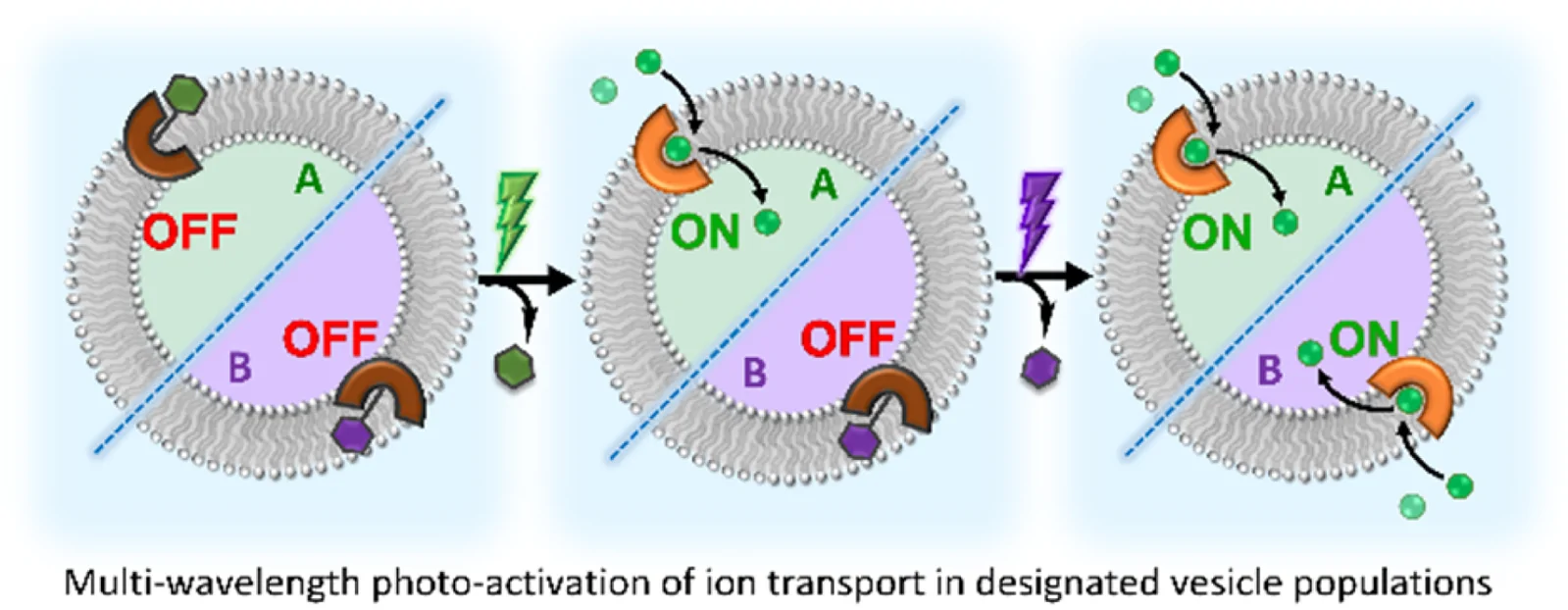

## Introduction

Many ion transporter proteins are regulated in a stimuli-responsive manner, such as in response to signalling molecules, membrane potential, and light. Their functions control homeostasis, signal transduction, and the overall maintenance of cellular and biological processes vital for life.^1^ Malfunction of these membrane proteins as a result of genetic mutations can lead to channelopathies, such as cystic fibrosis, which is caused by dysfunctional chloride channels in epithelial cells.^2,3^ Inspired by the potential to treat such diseases, there has been significant interest in developing synthetic ion transporters that mimic the functionality of natural membrane proteins and have the potential to bypass faulty ion channels or act as anticancer agents through disruption of the homeostasis of cancer cells, triggering apoptosis.^4–7^

Stimuli-responsive ion transporters are comparably rare; however, they offer the possibility of spatiotemporally targeted activation of ion transport.^8–10^ One stimulus that has gained increasing popularity in medicinal chemistry is the use of light, such as in photodynamic therapy and more recently in the field of photopharmacology.^11,12^ Such photocontrol provides a non-invasive and spatiotemporally precise mechanism to trigger the activation of the target compound, resulting in control over the corresponding chemical or biological processes.^13,14^ The development of synthetic photo-controlled ionophores not only enables the study of ion transport processes but also offers promising applications for spatiotemporally controlled therapeutics.^15^ Molecular photoswitches, such as azobenzenes and stilbenes, have been incorporated into photo-switchable transmembrane ion transporters, enabling transport to be reversibly switched between a transport-inactive (*E*) and transport-active (*Z*) state.^16–22^ Although reversible control is favourable, achieving high switching efficiency between the *E* and *Z* isomers without background activity in the OFF state is challenging.

An alternative molecular approach for the photocontrol of active compounds is the incorporation of photocages, which are photolabile protecting groups (PPGs) that are covalently bound to a target molecule.^23^ Protecting groups have been widely used in synthetic chemistry to protect certain functional groups during synthesis.^14^ Photocages provide spatiotemporal control over the release of active compounds by rendering them inactive until the photocage has been released by irradiation with light at the appropriate wavelength (Fig. 1). Talukdar and co-workers reported the first photocaged anion carrier, blocking the binding site of an indole-2-carboxamide receptor with an *ortho*-nitrobenzyl (*o*NB) photocage; upon irradiation at 365 nm, the *o*NB photocage was cleaved, restoring anion binding and transport activity.^24^ A number of other photocaged ionophores have since been reported in which the photocage inhibits ion binding,^25,26^ including those capable of inducing photo-triggered cancer cell death.^27–29^ An alternative strategy was reported by our group, using a photocage lipid anchor to reduce the mobility of an ion carrier in the membrane to deactivate transport, until photo-cleavage of the anchor regenerated carrier mobility and transport activity.^30^ Whilst photocaging is an irreversible process, the advantage of photocaged systems is that very precise control of activation is achieved only when the photolabile protecting group has been cleaved from the compound; providing explicit ON and OFF states.

**Fig. 1:**
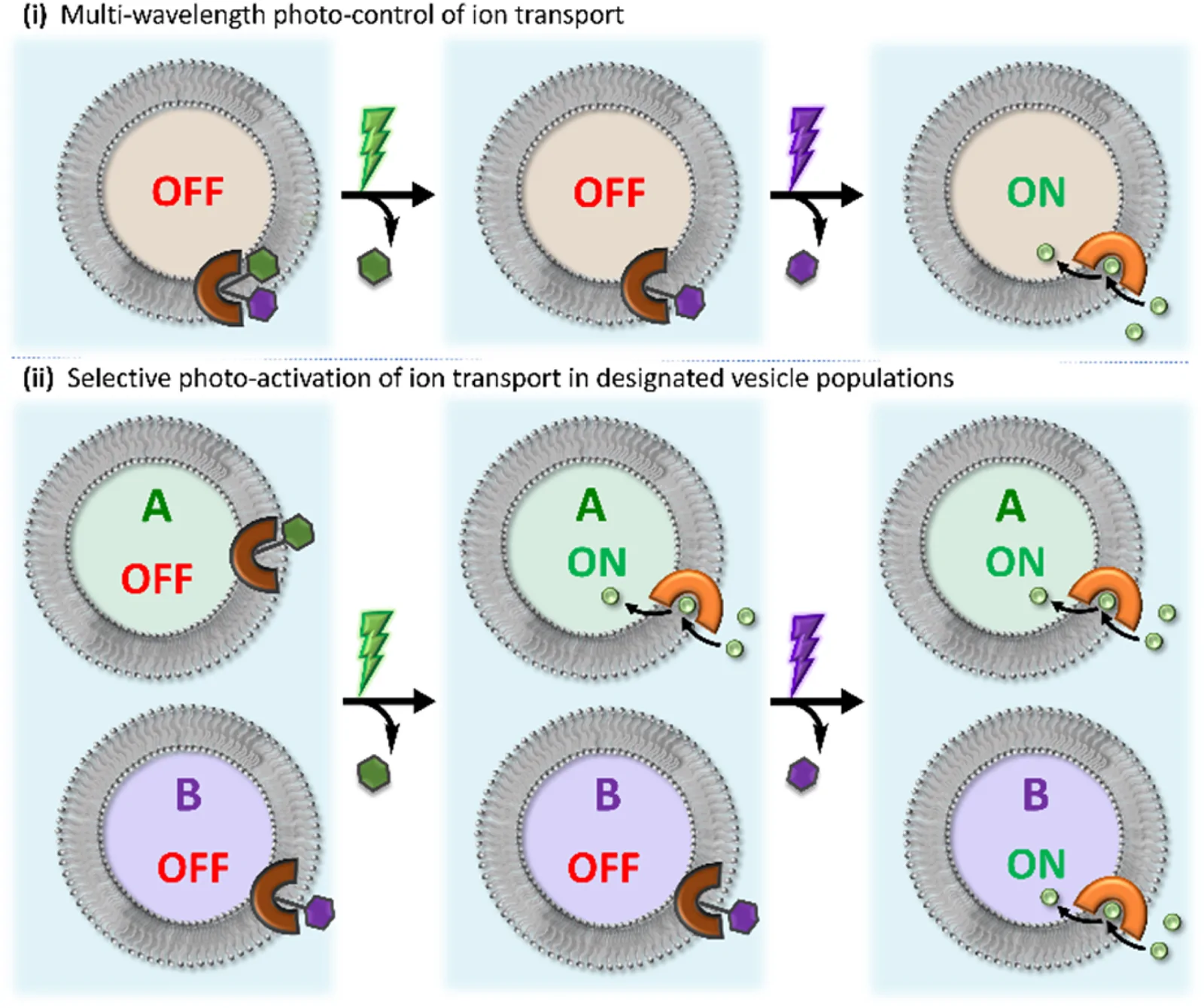
Schematic representation of multi-wavelength photo-triggered activation of ion transport by (i) sequential irradiation of a dual-photocaged ion transporter, or (ii) in designated vesicle populations A then B.

Herein, we report novel photo-responsive chloride transporters derived from a functionalised indolocarbazole scaffold. Photocaging of the anionophore using either one or two *o*NB photocages affords an inactive state, with transport activity inhibited until irradiation with light cleaves the photocage(s), unblocking the binding site and restoring transport activity. Functionalisation of the indolocarbazole scaffold with two different photocages responding to different wavelengths, *o*NB and boron dipyrromethane (BODIPY) photocages, provides access to an unprecedented dual-photocaged system. This enables enhanced control of transport activation *via* sequential irradiation using two different wavelengths of light, as well as photo-control over transport in different vesicle populations.

## Results and discussion

### Design, synthesis and transport experiments with indolocarbazole anionophores

To design an anionophore capable of multi-wavelength photocontrol, we first considered molecular structures capable of binding chloride and having the potential to prevent binding through direct functionalisation of the anion binding site with two photocages. We identified indolo[2,3-*α*]carbazoles as suitable for this purpose, being effective anion receptors that bind anions *via* their two preorganised hydrogen-bond-donating pyrrole groups.^31^ Accordingly, a series of indolo[2,3-*α*]carbazole derivatives were synthesised *via* an acid-catalysed Fischer indole synthesis (Schemes S1–S3).^32,33^ The parent compound indolo[2,3-*α*]carbazole 1 and two novel fluorinated derivatives 3,8-(trifluoromethyl)indolocarbazole 2 and 2,4,7,9-(trifluoromethyl)indolocarbazole 3 were synthesised (Fig. 2) to investigate the binding and transport capabilities and explore the effect on anion transport of incorporation of the lipophilic trifluoromethyl electron-withdrawing group. Full synthetic procedures and characterisation are available in the SI.

**Fig. 2:**
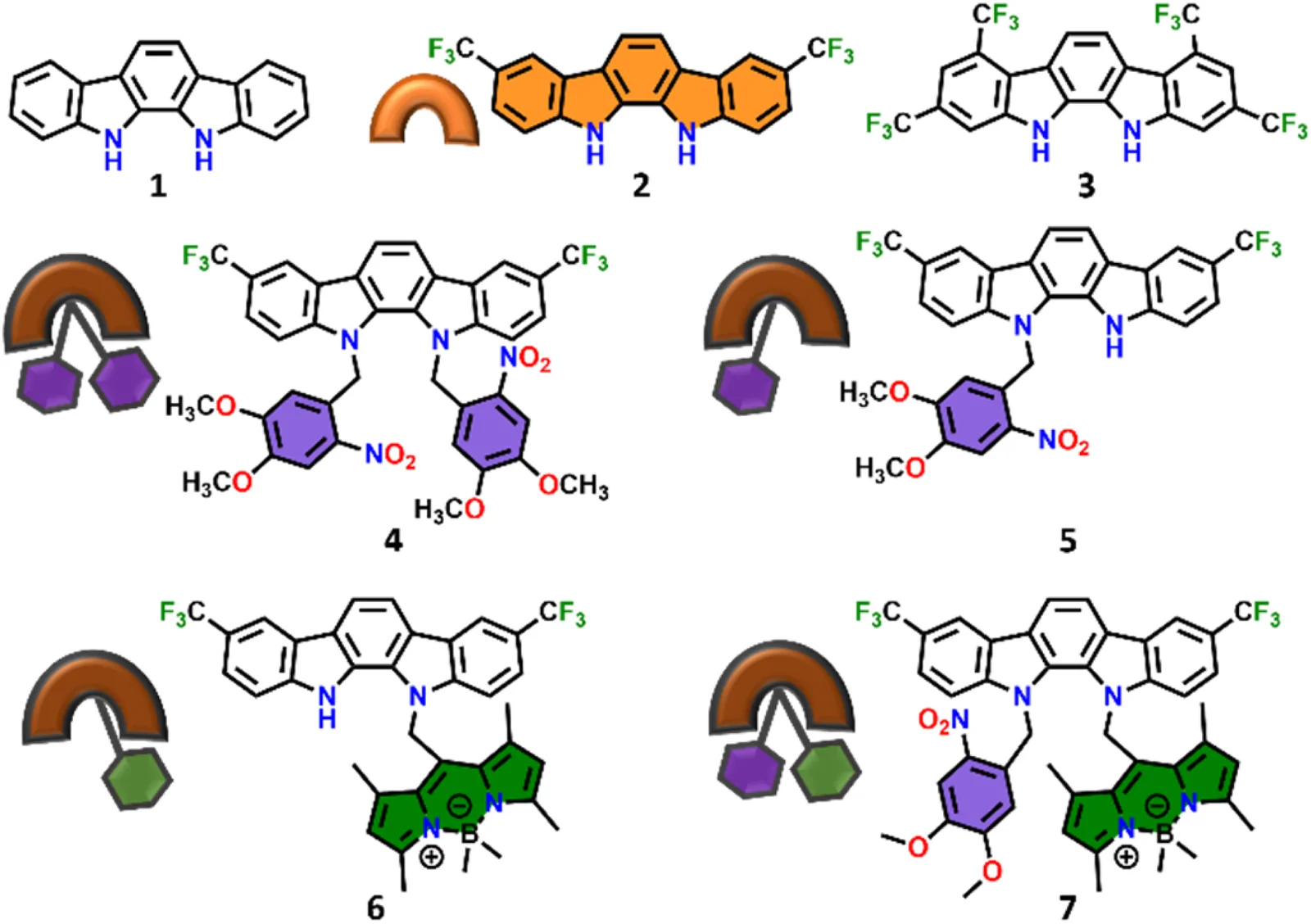
Indolo[2,3-*α*]carbazole anionophores 1–3 and double- (4), mono- (5, 6), and dual- (7) photocaged bis(trifluoromethyl)indolo[2,3-*α*]carbazoles.

We first explored the chloride anion binding capabilities of the indolo[2,3-*α*]carbazole derivatives, 1–3, by UV-visible spectroscopic titration experiments in acetonitrile with tetrabutylammonium (TBA) chloride. Bathochromic shifts, with new absorption maxima and corresponding isosbestic points, were observed with increasing additions of chloride, indicative of the formation of host–guest complexes.^34^ The concentration dependence of the changes in the absorbance spectra was fitted to a 1 : 1 stoichiometric host–guest binding isotherm using the Musketeer software (Fig. S26).^35,36^ This analysis revealed that the chloride binding affinity (Table 1) increased with increasing trifluoromethyl substitution and was greatest for the tetra(trifluoromethyl)-substituted indolocarbazole 3 (*K* = 8710 M^−1^), followed by the bis(trifluoromethyl)-substituted indolocarbazole 2 (*K* = 6705 M^−1^) and the unsubstituted indolocarbazole 1 (*K* = 1900 M^−1^).

**Table 1:** Summary of c log *P*, chloride binding *K* (M^−1^), EC_50_ (µM), and Hill coefficient (*n*) values^a^

Compound	c log *P*	*K* (M^−1^)/Cl^−^b	cEC_50_/µM	*n* d
1	4.1	1900 ± 80	—e	—
2	6.1	6705 ± 135	0.4	1.0 ± 0.08
3	8.1	8710 ± 440	>5^e^	1.1 ± 0.26

a Consensus *c* log *P* values were calculated using Swiss ADME.^37^.

b Binding constants were obtained by UV-vis spectroscopy (10 µM, in acetonitrile), using the Musketeer software to fit to a 1 : 1 binding model.

c Effective concentration to reach 50% of maximal activity across POPC LUVs (200 nm, lipid concentration 31 µM) encapsulating 1 mM HPTS, 100 mM NaCl, buffered at pH 7.0 using 10 mM HEPES.

d Hill coefficient.

e EC_50_ could not be reliably determined due to the lack of transport activity of 1 and insolubility of 3.

With evidence of strong chloride binding by the indolo[2,3-*α*]carbazole derivatives 1–3, their transmembrane chloride transport capabilities were explored in large unilamellar vesicles (LUVs). Transport activity was investigated in 1-palmitoyl-2-oleoyl-*sn-*glycero-3-phosphocholine vesicles (POPC LUVs, lipid concentration 31 µM), encapsulating the pH responsive fluorophore 8-hydroxypyrene-1,3,6-trisulfonic acid trisodium salt (HPTS) buffered to pH 7.0 in NaCl solution. HPTS acts as an internal probe to report pH changes across the lipid membrane induced by Cl^−^/OH^−^ antiport (or the functionally equivalent Cl^−^/H^+^ symport).^30^ The fluorescence intensity at 510 nm was monitored over time following excitation of the protonated and deprotonated forms of HPTS (405 nm and 460 nm, respectively) and the ratiometric output plotted for analysis. Ion transporter 2, with the strongest binding affinity for chloride, was most active in this anion transport assay, with an EC_50_ = 0.4 µM (Fig. 3 and Table 1). The corresponding values for 1 and 3 could not be reliably determined due to the lack of transport activity of the former and insolubility of the latter.

**Fig. 3:**
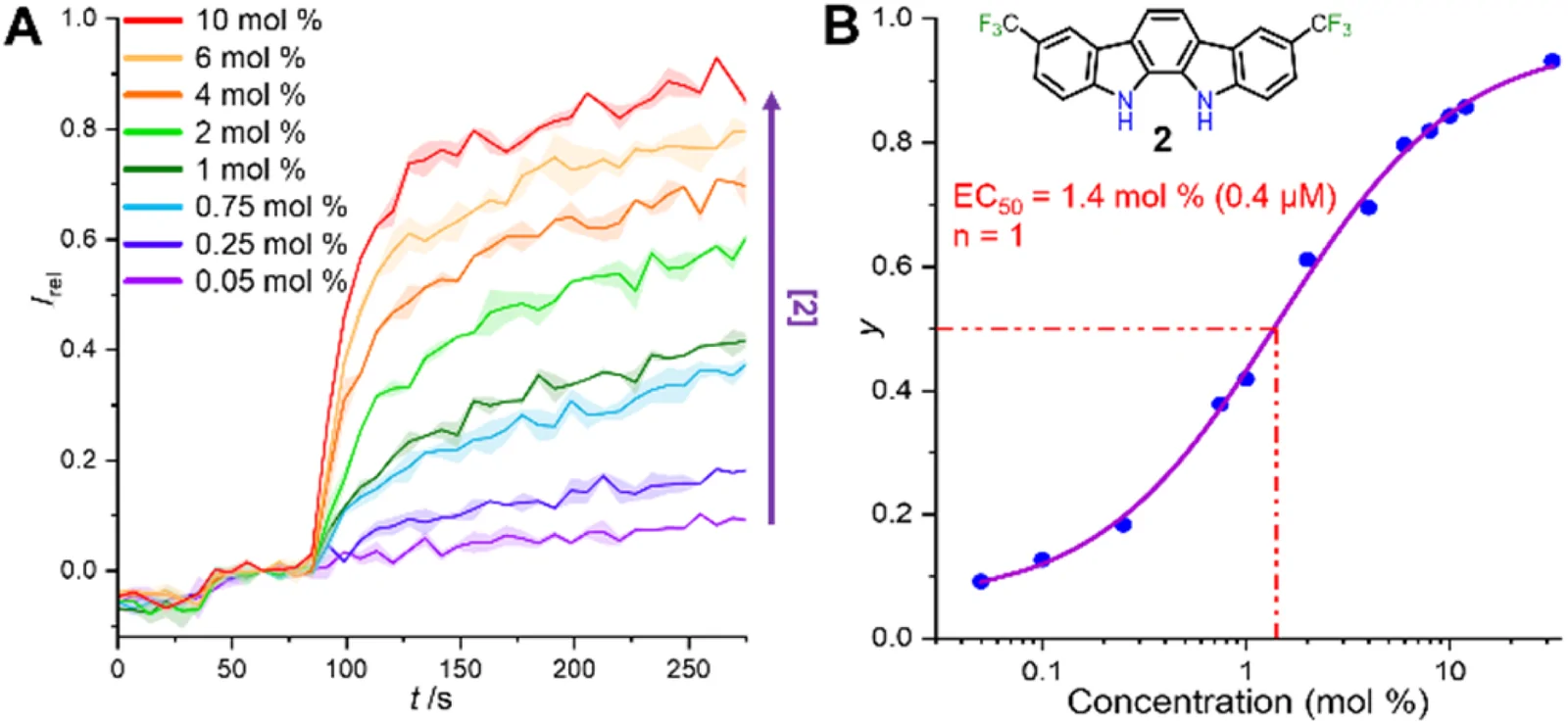
(A) Change in ratiometric emission, *I*_rel_ (*λ*_em_ = 510 nm; *λ*_ex1_ = 405 nm, *λ*_ex2_ = 460 nm) with increasing concentration of ion transporter 2 upon addition of a NaOH base pulse (5 mM) to POPC LUVs (31 µM) containing 1 mM HPTS, 100 mM internal and external NaCl, buffered with 10 mM HEPES at pH 7.0. (B) Dependence of fractional activities (*y*, the relative intensity at *t* = 276 s immediately prior to lysis) on concentration of 2, (●), and fitted to the Hill equation (■).

A lack of transport activity by 2 when chloride was replaced by the larger and more hydrophilic gluconate anion, which is not typically transported by anionophores, confirmed that 2 transports *via* a cation independent Cl^−^/OH^−^ antiport (or H^+^/Cl^−^ symport) process and not *via* a cation-dependent H^+^/Na^+^ antiport mechanism (Fig. S47).

To determine whether 2 facilitates transport as a mobile carrier or as a membrane-spanning channel, the HPTS assay was performed using 1,2-dipalmitoyl-*sn*-glycero-3-phosphocholine (DPPC) LUVs. No transport was observed at room temperature where DPPC is in a gel phase below its phase transition temperature (*T*_m_ = 41 °C), inhibiting mobility (Fig. S48). Above the phase transition temperature, at 45 °C, ion transport activity was restored, indicating that ion transporter 2 mediates transport *via* a carrier mechanism. Finally, the selectivity of 2 for chloride over proton or hydroxide transport was explored by addition of carbonyl cyanide 4-(trifluoromethoxy)-phenylhydrazone (FCCP), a protonophore that effectively uncouples H^+^/OH^−^ transport from that of Cl^−^ (Fig. S49). The transport activity of 2 was unchanged in the presence of FCCP, revealing a lack of selectivity for Cl^−^ over H^+^/OH^−^.

### Mono- and dual-caged indolocarbazole anionophores

3,8-(Trifluoromethyl)indolocarbazole (2), with the optimum binding and transport activity of the indolocarbazole derivatives, was selected for photocaging experiments. The two pyrrole groups of the indolo[2,3- *α*]carbazole were amenable to functionalisation with the photolabile protecting group dimethoxy-2-nitrobenzyl *via* alkylation with the corresponding benzyl bromide (for synthesis and characterisation, see the SI). Double-caging (compound 4) of the indolocarbazole was anticipated to inhibit binding and transport by blocking both NH H-bond donors in the binding site. To determine if a single photocage would be sufficient to block binding, a mono-caged transporter 5 was also synthesised.

The anion binding capabilities of the double- and mono-photocaged derivatives were evaluated by ^1^H NMR titration experiments. Compounds 4 and 5 were dissolved in acetone-*d*_6_ : D_2_O (98 : 2 v/v) at a concentration of 1 mM. ^1^H NMR spectra were recorded upon successive additions of aliquots of TBACl. As anticipated, no perturbation of the proton shifts was observed, indicating that no anion binding occurs with the double-photocaged derivative 4 (Fig. 4). Minimal chloride binding was observed for mono-photocaged transporter 5 (*K*_1:1_ = 31 M^−1^, Fig. S31).

**Fig. 4:**
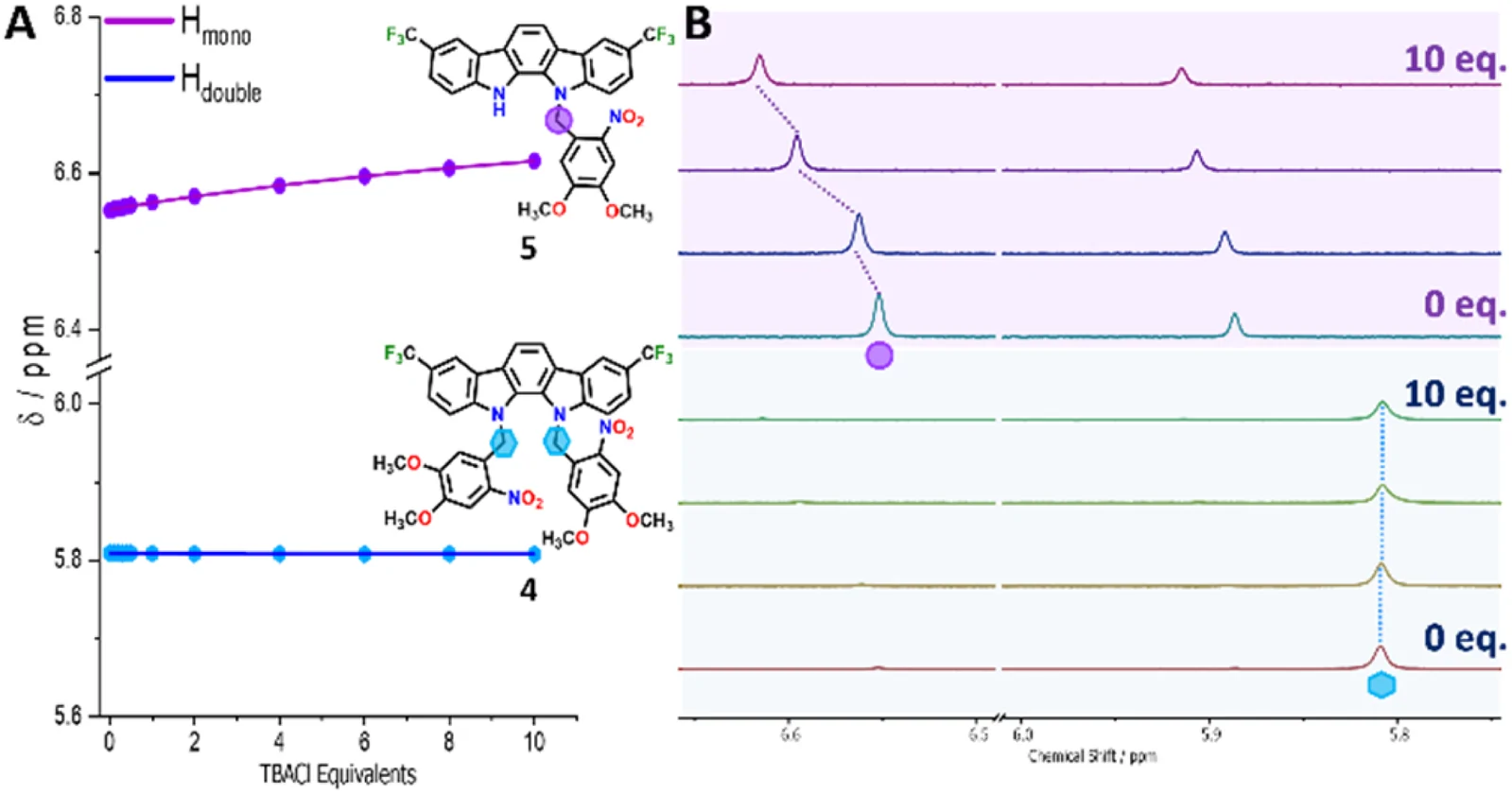
(A) 1 : 1 binding isotherms for 4 and 5 during the titration with increasing tetrabutylammonium (TBA) chloride in 2.0% D_2_O in acetone-*d*_6_. Experimental data (●), fitted to a 1 : 1 binding isotherm (■). (B) Stacked partial ^1^H NMR spectra of 4 (bottom) and 5 (top) showing 0, 1, 6, and 10 equivalents of TBACl.

The photo-triggered activation of the double- (4) and mono- (5) caged ion transporters was then explored using the HPTS anion transport assay, following *in situ* activation of the compounds within the vesicle membrane. The caged compound was added to POPC LUVs encapsulating HPTS in an aliquot of DMSO, and then subjected to photo-irradiation at 405 nm using an LED (1 W) for various periods of time, prior to initiating the transport assay (Fig. 5). For both the double- and mono-caged ion transporters, transport activity is inhibited by the presence of either one or two photocages, in agreement with results from chloride binding titrations, and could be restored by photo-irradiation, with the observed transport activity increasing with irradiation time. Transport activity comparable to that of the uncaged transporter 2 (Fig. 3) was observed after 300 seconds of irradiation of the mono-photocaged 5, indicative of near quantitative decaging in the membrane.

**Fig. 5:**
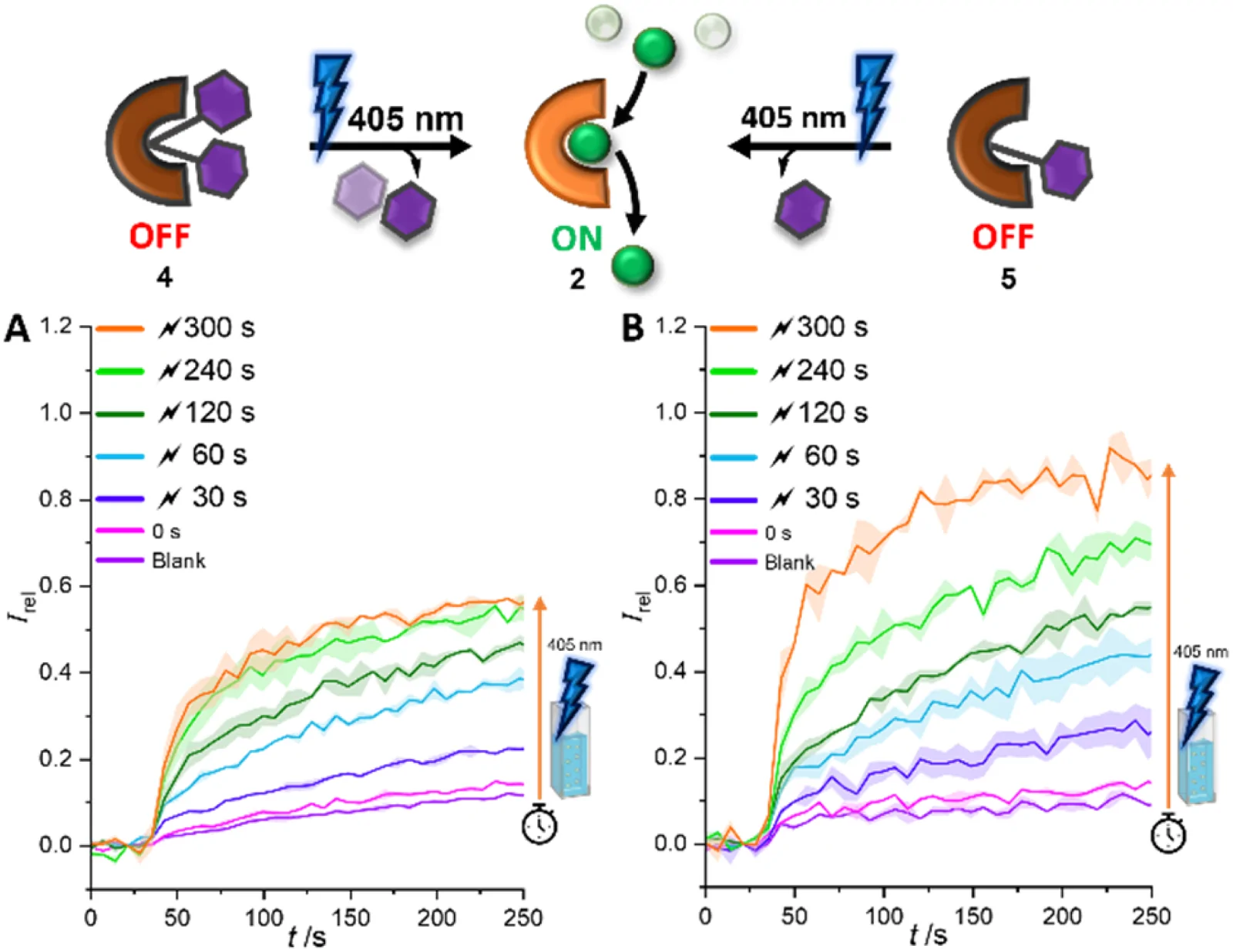
Change in ratiometric emission, *I*_rel_ (*λ*_em_ = 510 nm; *λ*_ex1_ = 405 nm, *λ*_ex2_ = 460 nm), with increasing *in situ* irradiation time of photocaged transporters with a 405 nm LED (1 W) for (A) double- (4), and (B) mono- (5) photocaged bis(trifluoromethyl)indolo[2,3-*α*]carbazoles. Assay conditions as in Fig. 3.

### Sequential activation of dual-caged indolocarbazole 7

The inactivity of mono-photocaged transporter 5 demonstrates that just one photocage is required to fully suppress anion transport. This suggested that a dual-photocaged system, in which each cage is responsive to different wavelengths of light and thus requires both cages to be removed to trigger anion transport, should be accessible.

Photo-cleavage of two different wavelength-absorbing photocages may, in principle, occur *via* either orthogonal or sequential activation. Orthogonal activation in practice requires negligible overlap of the two chromophores, which is difficult to achieve, especially at shorter wavelengths.^23^ However, sequential activation is possible provided one photocage is sufficiently red-shifted and separated from its blue-shifted partner, such that it can be selectively excited. Irradiating at the non-overlapping longer wavelength first, followed by the shorter wavelength cage, enables a two-step sequence in cleaving the substrate. Despite the obvious attractiveness of achieving high levels of spatial and temporal control from photo-activation requiring multiple wavelengths, there are only a handful of reported systems in which two photocages are employed together on one substrate. For example, Winter and co-workers investigated wavelength-selective release of succinic acid using a BODIPY and coumarin photocage, and another using two different BODIPY-based photocages.^38^ For both photocaged analogues, only sequential activation was achieved when irradiated at the lower-energy-longer wavelength first, followed by the higher-energy, shorter wavelength, due to overlap of the respective absorption spectra. To the best of our knowledge, no synthetic ionophores exist that utilise two different photocages, requiring two-wavelength irradiation (sequential or orthogonal), to restore ion transport activity.

With a mono-caged dimethoxy-*o*NB transporter already in hand, a BODIPY photocage was chosen as the second photocage due to its significantly red-shifted absorbance profile (Fig. 6A). Accordingly, a dual-photocaged derivative (7) was synthesised by alkylating 4 with the corresponding bromomethyl-BODIPY photocage (Scheme S7), and the wavelength selectivity of cleavage was investigated by ^1^H NMR (1 mM, DMSO-*d*_6_). Irradiation at 530 nm using an LED (370 mW) resulted in selective cleavage of the BODIPY photocage, as observed by the appearance of the ^1^H peak corresponding to the pyrrole proton of the mono-photocaged dimethoxy-*o*NB analogue (5) (Fig. 6B). Sequential irradiation at 365 nm resulted in cleavage of the dimethoxy-*o*NB photocage and appearance of the pyrrole protons of the non-photocaged transporter (2) (Fig. S41). In contrast, irradiating 7 first with 365 nm light produced a mixture of mono-photocaged (5) and non-photocaged (2) analogues, with no observation of the formation of mono-photocaged BODIPY (6) (Fig. S42). This is indicative of faster cleavage of the BODIPY photocage under these conditions.

**Fig. 6:**
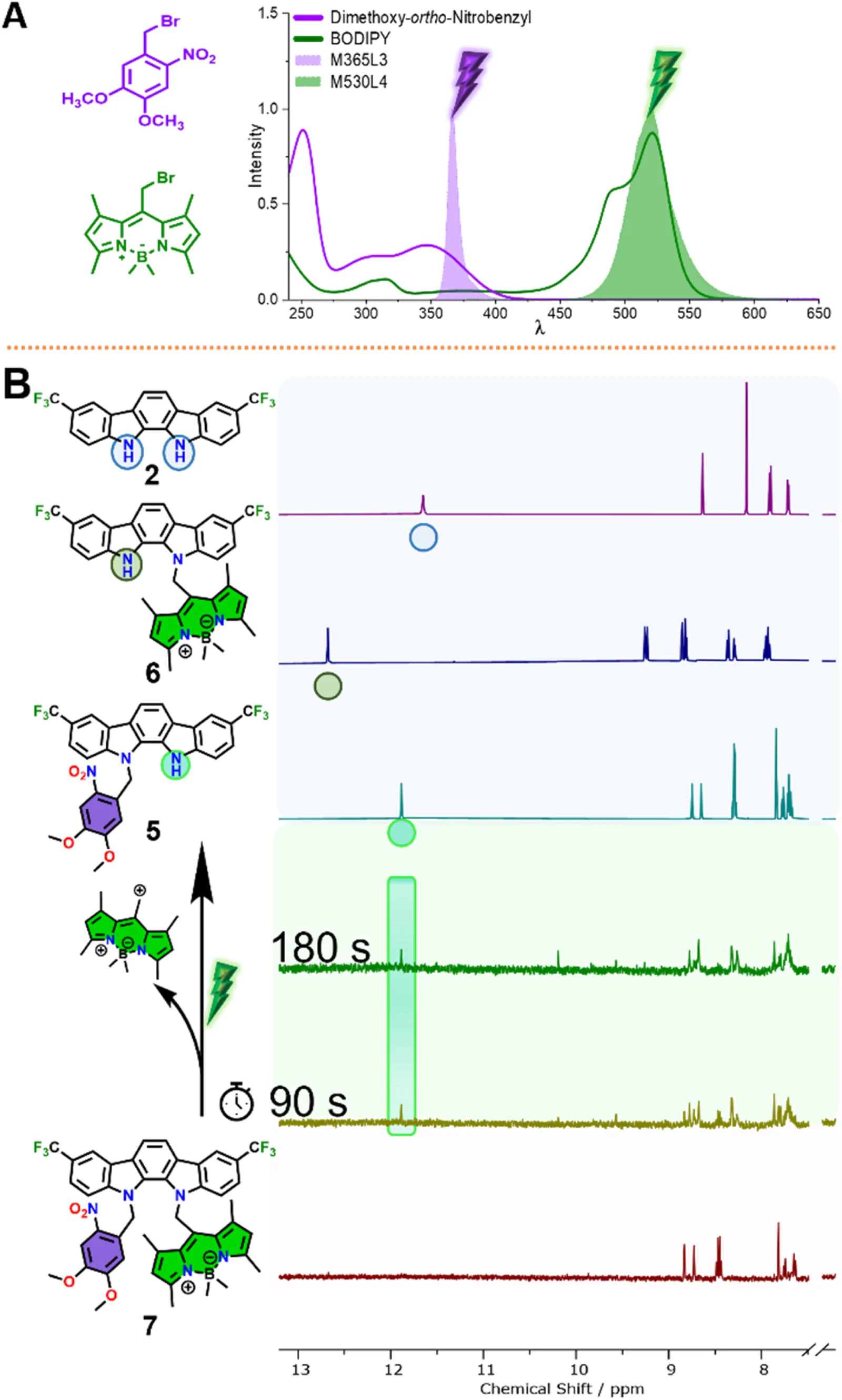
(A) Absorbance profiles of dimethoxy-*o*NB and BODIPY photocages, and the emission profile of the Thorlabs M365L3 (880 mW) and M530L4 (370 mW) LEDs centred at 365 nm (purple) and 530 nm (green). (B) Stacked ^1^H NMR spectra of dual-photocaged (7) with increasing irradiation times at 530 nm, in comparison to the ^1^H NMR spectra of reference samples of mono-photocaged (5), mono-photocaged (6), and non-photocaged ion transporter (2).

With a multi-wavelength sequentially activated anion receptor in hand, we investigated the activation of chloride transport *via* sequential irradiation using the HPTS assay in LUVs (Fig. 7). Consistent with the results of the NMR photo-decaging experiments, irradiating first with 365 nm resulted in full restoration of chloride transport (Fig. 7ii), indicative of full cleavage of both the *o*NB and BODIPY photocages. Sequential multi-wavelength photocontrol over transmembrane chloride transport could be achieved by irradiation first with the 530 nm LED, followed by 365 nm. After irradiation with 530 nm to generate the transport-inactive mono-(*o*NB)-photocaged (5), minimal transport activity was observed after 480 s of irradiation (Fig. 7i), and extended irradiation to 1200 s did not further increase activity (Fig. S54B), indicative of selective cleavage of the BODIPY photocage. Subsequent irradiation at 365 nm restored chloride transport (Fig. 7iii) by removing the remaining *o*NB photocage.

**Fig. 7:**
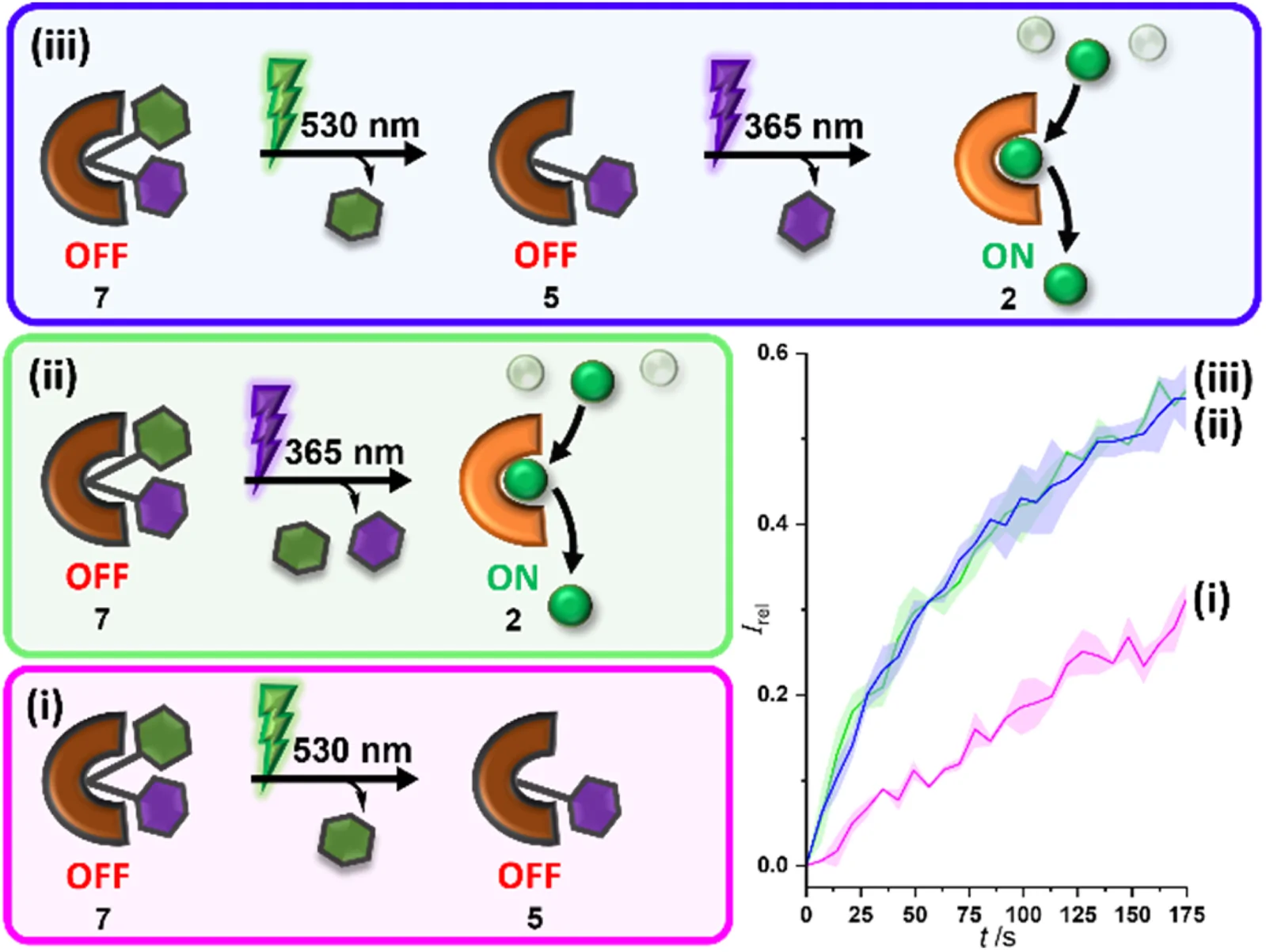
Photo-triggered activation of chloride transport of dual-photocaged (7). Change in ratiometric emission, *I*_rel_ (*λ*_em_ = 510 nm; *λ*_ex1_ = 405 nm, *λ*_ex2_ = 460 nm) with *ex situ* irradiation of 7, upon addition of a NaOH base pulse (5 mM) to POPC LUVs (31 µM) containing 1 mM HPTS, 100 mM internal and external NaCl, buffered with 10 mM HEPES at pH 7.0. (iii) Transport activity is fully restored with sequential irradiation at 530 nm for 1200 s followed by 480 s of irradiation at 365 nm; (ii) unselective cleavage at 365 nm (720 s) resulting in immediate restoration of chloride transport activity; (i) negligible transport is observed following irradiation for 480 s at 530 nm.

### Temporal photocontrol over ion transport in distinct vesicles in a mixed population

With a sequential activation system established and successful inhibition of transport achievable with mono-photocaged transporters, we explored the potential of an enhanced temporally-controlled transport system in which different wavelengths of light are used to activate transport in distinct populations of vesicles. Mono-photocaged analogues 5 and 6 were incubated in separate suspensions of POPC LUVs encapsulating HPTS. Two vesicle populations were formed, which we label A and B, each incorporating within their membranes either mono-photocaged BODIPY (6), or mono-photocaged dimethoxy-*o*NB (5), respectively, at a 4 mol% loading with respect to lipid. Suspensions of vesicles A and B were combined to form a 50 : 50 mixture of the two populations (Fig. 8i). Control experiments revealed that the hydrophobic ionophores were not able to diffuse between vesicles once incorporated (Fig. S57).

**Fig. 8:**
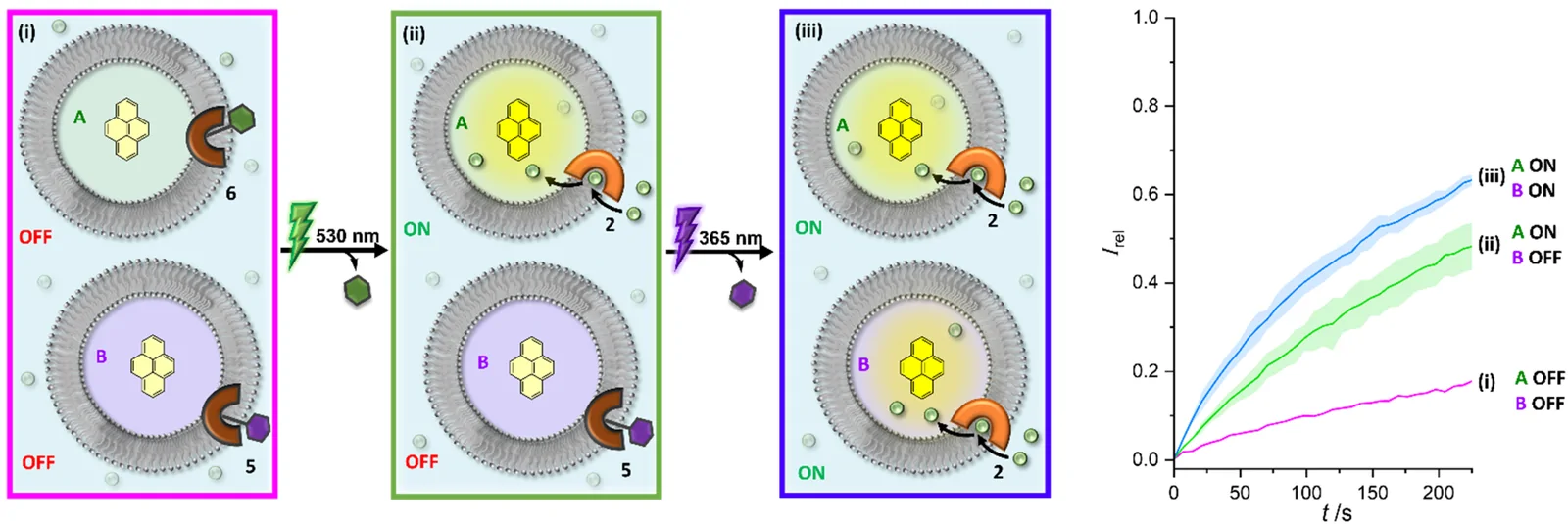
Sequential photo-activation of chloride transport in two populations of vesicles. Vesicles A contain mono-BODIPY-caged 6 (4 mol% w.r.t lipid) and vesicles B contain mono-*o*NB-caged 5 (4 mol% w.r.t. lipid). Plot shows change in ratiometric emission, *I*_rel_, (*λ*_em_ = 510 nm; *λ*_ex1_ = 405 nm, *λ*_ex2_ = 460 nm) following initiation of transport with a NaOH base pulse (5 mM) to POPC LUVs (31 µM) containing 16 mM HPTS, 100 mM internal and external NaCl, buffered with 10 mM HEPES at pH 7.0, for (i) a 50 : 50 mixture of vesicles A and B prior to irradiation; (ii) activity following irradiation at 530 nm for 720 s, activating transport in vesicles A; (iii) activity following irradiation at 530 nm for 720 s and then at 365 nm for 120 s, activating transport in vesicles A and B sequentially.

The suspension of vesicles was first irradiated with the 530 nm LED to selectively activate photocaged anionophore 5 in population A (Fig. 8), and the observed transport activity was monitored as previously described *via* changes in the HPTS fluorescence. The observed initial rate of anion transport was *k* = 4.2 × 10^−3^ s^−1^. In a separate experiment, the vesicle mixture was first irradiated at 530 nm, followed by irradiation at 365 nm, to sequentially activate first photocaged anionophore 5 in vesicles A and then compound 6 in vesicles B. In this experiment, in which the anionophores in both populations of vesicles were activated, the observed initial rate of anion transport increased ∼2-fold to *k* = 7.1 × 10^−3^ s^−1^. Given that our previous experiments revealed that photo-irradiation under the same conditions leads to near quantitative activation of anionophore 2, which is present in equal concentrations in both populations and has equivalent activities, this doubling of observed transport rates is consistent with sequential activation of transport in both vesicle populations. To the best of our knowledge, this represents the first example of a synthetic transport system that can be activated in different populations of vesicles within the same system upon the application of different stimuli.

## Conclusions

We have developed a new class of photo-responsive chloride transporters based on an indolocarbazole scaffold, in which incorporation of photolabile protecting groups provides excellent OFF–ON control over transmembrane transport activity. Mono- and double-dimethoxy-*o*-nitrobenzyl photocaged derivatives were shown to fully inhibit chloride binding and transport, with activity restored upon decaging by photo-irradiation. Extension to BODIPY photocaging enabled access to a unique dual-photocaged system, in which sequential multi-wavelength irradiation affords unprecedented temporal control over transporter activation. Furthermore, sequential irradiation of distinct vesicle populations containing different mono-photocaged ionophores demonstrated selective and stepwise activation of anion transport in each vesicle population, offering a strategy to control ion transport in complex multi-cellular environments. These results establish multi-wavelength photocaged indolocarbazoles as a versatile platform for engineering spatiotemporally regulated ion transport and open new avenues for the development of synthetic transporters for controlling transmembrane ion flux in synthetic and living cells or for dose-controlled release of molecular cargo.

## Author contributions

K. R. B. synthesised the compounds and carried out the experimental work. M. J. L. conceived and supervised the project. Both authors analysed the data and wrote the article.

## Conflicts of interest

There are no conflicts to declare.

## Supplementary Material

SC-017-D6SC04824B-s001

## Data Availability

The data supporting this article have been included as part of the supplementary information (SI). Supplementary information is available. See DOI: https://doi.org/10.1039/d6sc04824b.
