# Supplementary material for: Multi-wavelength photo-activation of transmembrane chloride transport in distinct vesicle populations using photocaged indolocarbazole anionophores
Source: Chem Sci. 2026 Aug 26;17(41):20036–42. doi: 10.1039/d6sc04824b (PMC13557027; doi:10.1039/d6sc04824b)
Supplement: Supplementary file 1 [file SC-017-D6SC04824B-s001.pdf]

Supplementary Information (SI) for Chemical Science.
This journal is © The Royal Society of Chemistry 2026

Supporting Information for

Multi-wavelength Photo-activation of Transmembrane Chloride Transport in Distinct Vesicle Populations using Photocaged Indolocarbazole Anionophores

Kelly R. Britton [a] and Matthew J. Langton*[a]

[a] Department of Chemistry, University of Oxford, 12 Mansfield Road, Oxford, OX1 3TA, UK

Contents

Contents 1
1. Materials and Methods 2
1.1 Abbreviations 3
2 Synthesis and Characterisation 4
3 UV-Vis Titration Experiments 26
4 ^{1}H NMR Titration Experiments 28
5 Photocleavage Experiments 31
5.1 Irradiation Set-up 31
5.2 UV-Vis Analysis 31
5.3 ^{1}H NMR Analysis 33
6 Anion Transport Experiments 39
6.1 Vesicle Preparation 39
6.2 The HPTS Assay 40
6.3 Sodium Gluconate HPTS Assay 42
6.4 HPTS Assay with DPPC Lipids 43
6.5 HPTS Assay with FCCP 44
6.6 Photo-triggered HPTS Transport Assay 45
6.7 Photo-triggered HPTS Transport Assay in Designated Vesicle Populations 50
7 References 53

1. Materials and Methods

All reagents and solvents were purchased from commercial sources and used without further purification. Lipids were purchased from Avanti Polar Lipids and used without further purification. Where necessary, solvents were dried by passing through an MBraun MPSP-800 column and degassed with nitrogen. Triethylamine was distilled from and stored over potassium hydroxide. Column chromatography was carried out on Merck® silica gel 60 under a positive pressure of nitrogen. Where mixtures of solvents were used, ratios are reported by volume. GPC purification of vesicles was carried out using GE Healthcare PD10 desalting columns prepacked with Sephadex G 25 medium. NMR spectra were recorded on a Bruker AVIII 400, Bruker AVII 500 (with cryoprobe) and Bruker AVIII 500 spectrometers. Chemical shifts are reported as δ values in ppm. Mass spectra were carried out on a Waters Micromass LCT and Bruker microTOF spectrometers. Fluorescence spectroscopic data were recorded using a Horiba Duetta (or Agilent Cary eclipse) fluorescence spectrophotometer, equipped with Peltier temperature controller and stirrer. UV-Vis absorption spectra were recorded on a V-770 UV-Visible/NIR Spectrophotometer equipped with Peltier temperature controller and stirrer. For absorption spectra in scattering samples, a temperature controlled 60 mm integrating sphere (JASCO) was used. Vesicles were prepared as described below using Avestin "LiposoFast" extruder apparatus, equipped with polycarbonate membranes with 200 nm pores. When using lipids with a phase transition temperature above RT, the Avanti Mini Extruder (equipped with a heating block) was used instead.

1.1 Abbreviations

BODIPY: Boron-dipyrromethene

DCM: Dichloromethane

DMF: Dimethylformamide

DPPC: 1,2- Dipalmitoyl-sn-glycero-3-phosphocholine

EtOAC: Ethyl acetate

EtOH: Ethanol

FCCP: Carbonyl cyanide-p-trifluoromethoxyphenylhydrazone

HEPES: 4-(2-Hydroxyethyl)-1- piperazineethanesulfonic acid

HOAc: Acetic acid

HPTS: 8-Hydroxypyrene-1,3,6-trisulfonic acid trisodium salt

HRMS: High resolution mass spectrometry

H_2SO_4: Sulfuric acid

LUV: Large unilamellar vesicles

$MgSO_4$: Magnesium sulfate

POPC: 1-Palmitoyl-2- oleoyl-sn-3-phosphatidylcholine

rt: Room temperature

TFA: Trifluoroacetic acid

2 Synthesis and Characterisation

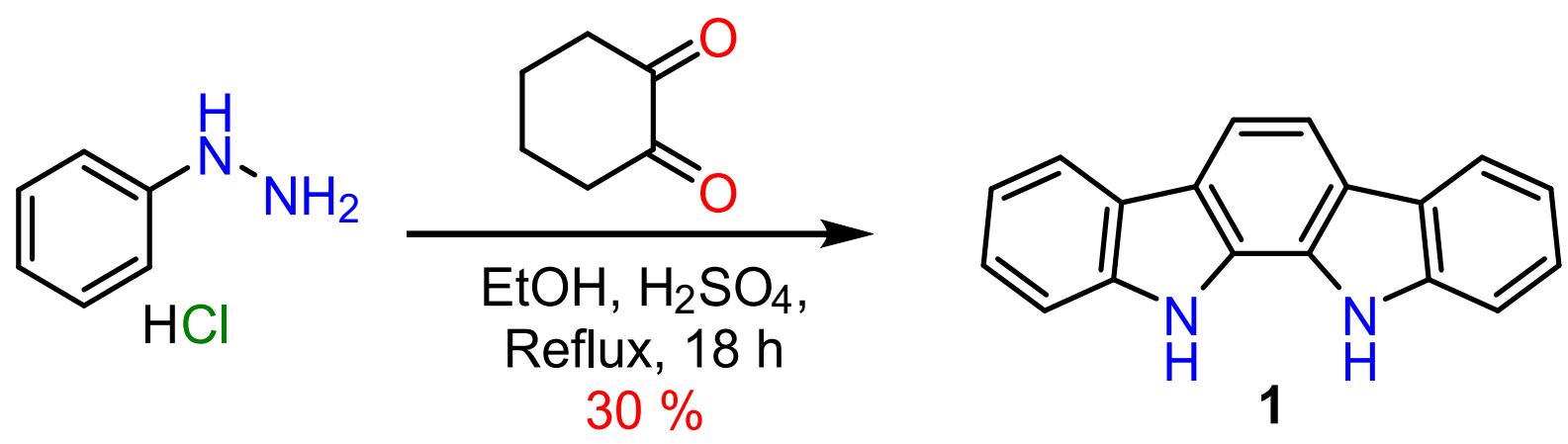

Scheme S1: Synthesis of indolo[2,3-α]carbazole **1**

Transporter 1. Prepared by a modified procedure.[1] 1,2-Cyclohexanedione (0.56 g, 4.99 mmol, 1 equiv.) and phenylhydrazine (1.47 mL, 15.0 mmol, 3 equiv.) were dissolved in EtOH (50 mL), and concentrated H_2SO_4(aq) (1 mL) was added dropwise *via* syringe. The solution was heated to reflux for 18 h, and then deionized H_2O (200 mL) was added and left to stir at rt for 1 h. The precipitate was filtered and washed with H_2O (5 x 15 mL). The crude product was dry-loaded onto silica and purified by silica flash column chromatography in hexane: ethyl acetate 9:1 graded to 7:3. The solvent was evaporated *in vacuo* to yield the product as a pinkish-beige crystalline solid (0.39 g, 1.50 mmol, 30 %). ^{1}H NMR (400 MHz, DMSO-d_6) δ 11.07 (s, 2H, N*H*), 8.15 (d, *J* = 7.7 Hz, 2H), 7.91 (s, 2H), 7.70 (d, *J* = 8.1 Hz, 2H), 7.39 (t, *J* = 7.5 Hz, 2H), 7.21 (t, *J* = 7.4 Hz, 2H). Data consistent with the literature.[2]

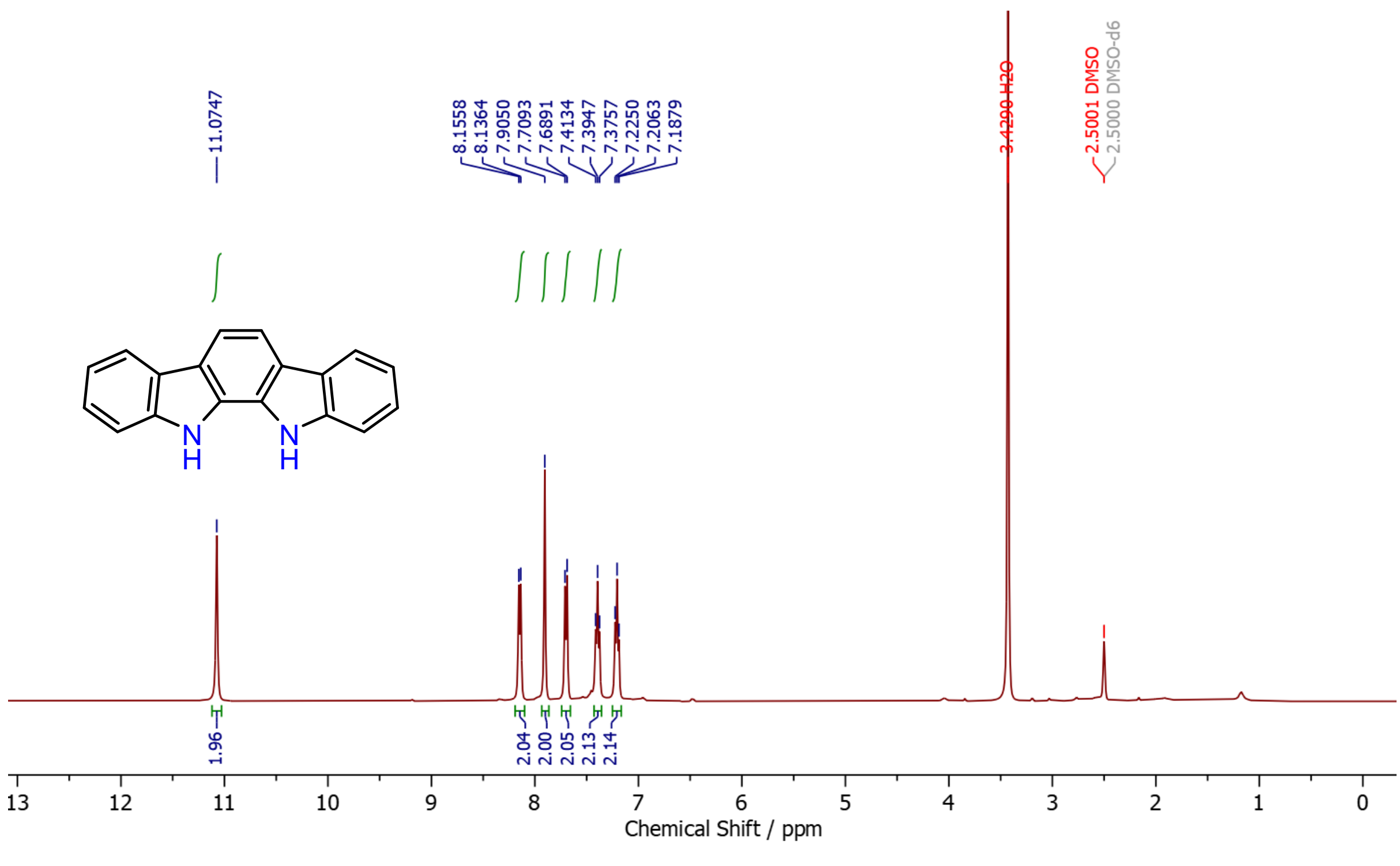

Figure S1: ^{1}H NMR Spectrum of **1** (DMSO-d_6, 298 K, 400 MHz)

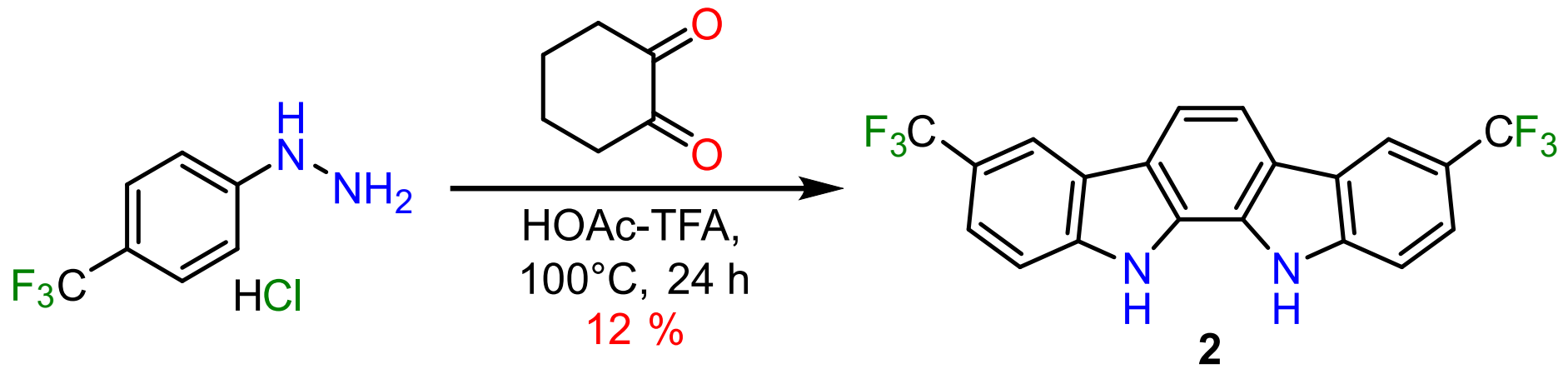

Scheme S2: Synthesis of 3,8-(trifluoromethyl)-indolo[2,3-α]carbazole **2**

Transporter 2. Prepared by a modified procedure.[3] 1,2-Cyclohexanedione (187.6 mg, 1.67 mmol, 1 equiv.) and 4-trifluoromethyl phenylhydrazine hydrochloride (1.07 g, 5.03 mmol, 3 equiv.), were dissolved in HOAc (7.5 mL) and TFA (2.5 mL). The solution was heated to 100°C for 24 h, and then cooled to rt. Deionized H_2O (100 mL) was added and the precipitate was filtered and washed with H_2O (5 x 15 mL). The crude product was dry-loaded onto silica and purified by silica flash column chromatography in hexane: ethyl acetate 9:1. The solvent was evaporated *in vacuo* to yield the product as a light brown-beige crystalline solid (76.5 mg, 0.20 mmol, 12 %). ^{1}H NMR (600 MHz, DMSO-d_6) δ 11.65 (s, 2H), 8.64 (s, 2H), 8.16 (s, 2H), 7.91 (d, *J* = 8.5 Hz, 2H), 7.72 (dd, *J* = 8.6, 1.8 Hz, 2H). ^{13}C NMR (151 MHz, DMSO-d_6) δ 140.8, 128.3 – 122.9 (q), 126.4, 123.3 - 121.2 (q), 120.6, 120.0 - 119.4 (q), 117.7 - 117.6 (q), 112.9, 112.3, 40.1. ^{19}F NMR (565 MHz, DMSO-d_6) δ -58.14. HRMS-ESI (m/z) calculated for $[C_{20}H_{10}F_6N_2]^+$, $[M]^+$: 392.0743, found: 392.0738

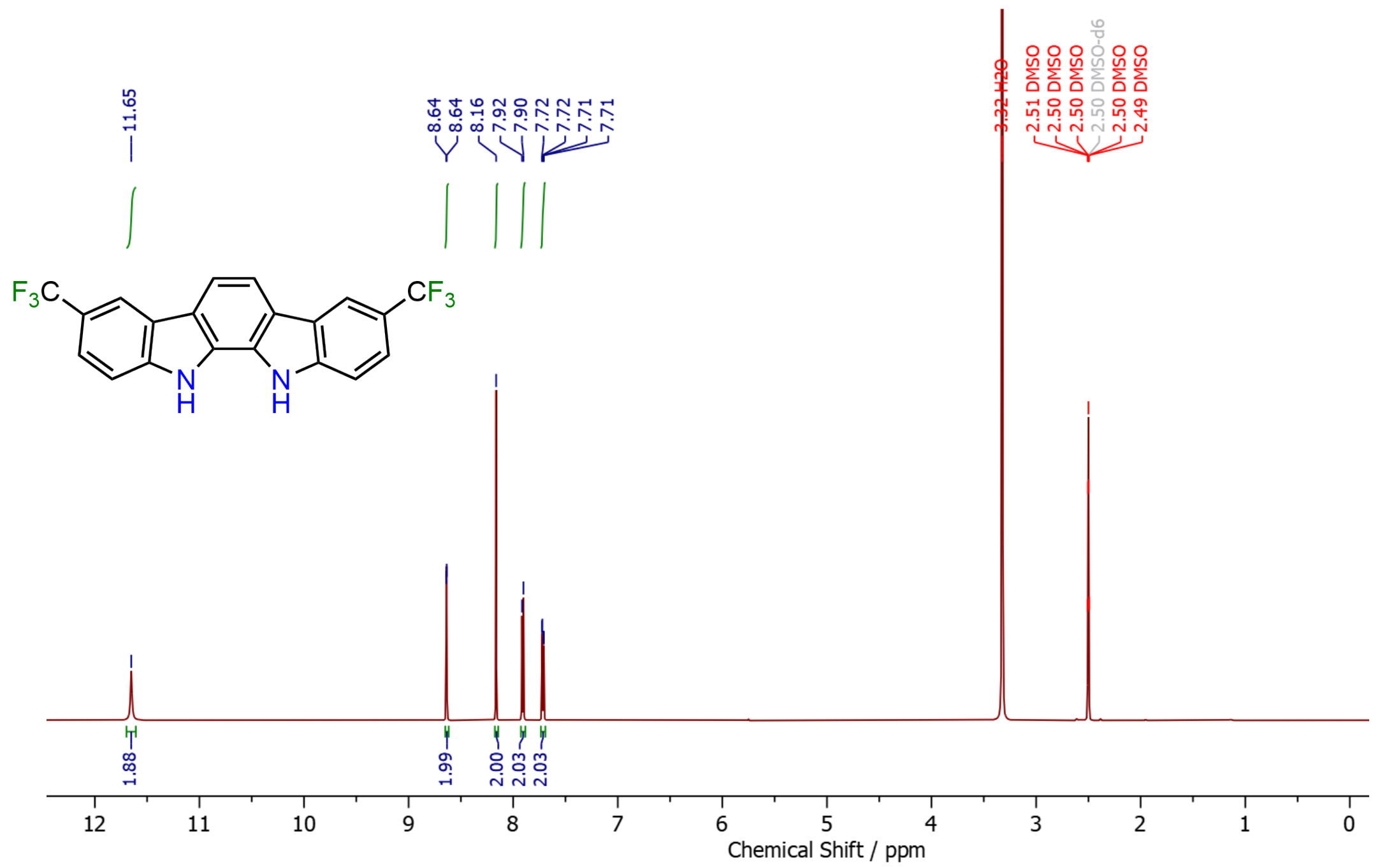

Figure S2: ^{1}H NMR Spectrum of **2** (DMSO-d_6, 298 K, 600 MHz)

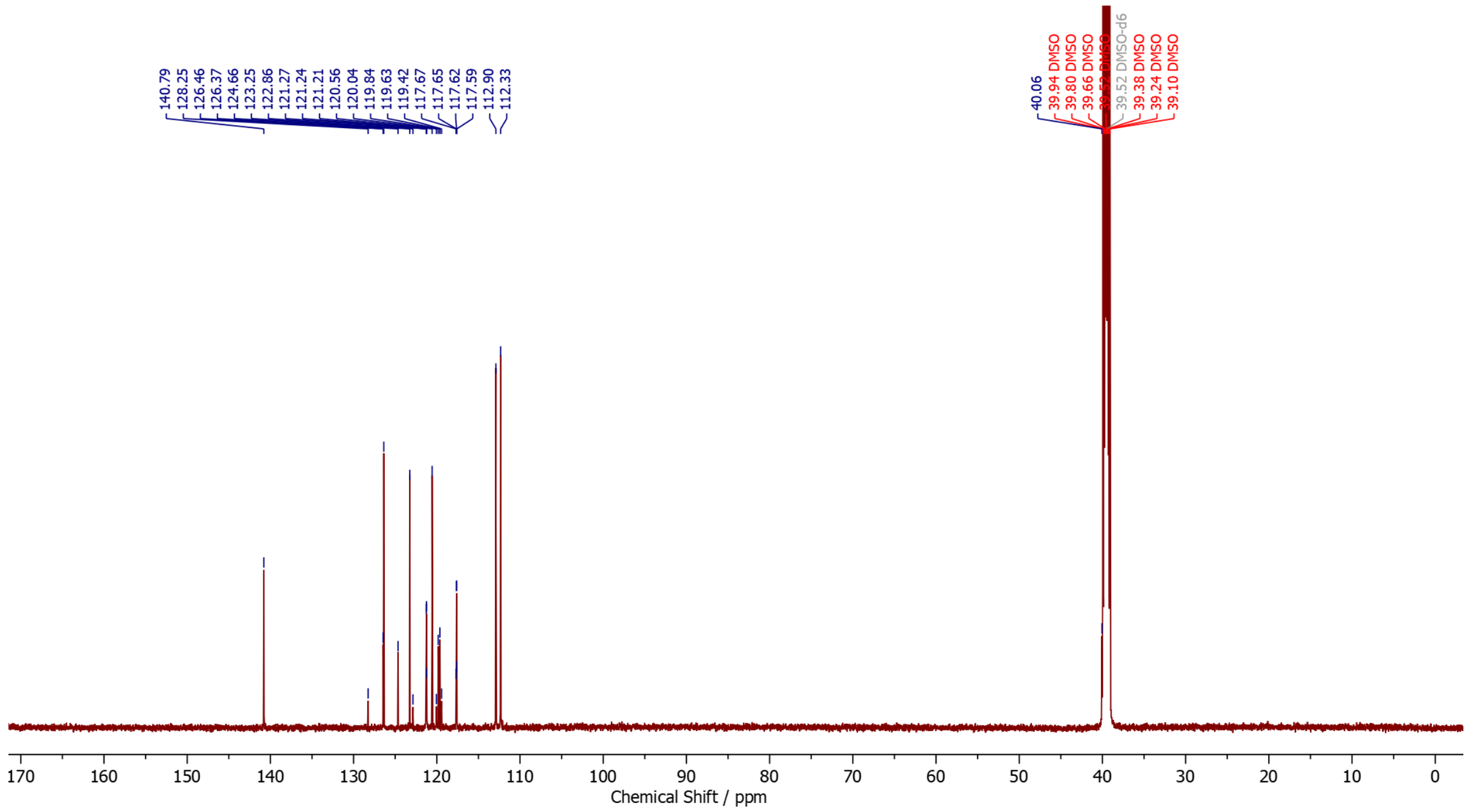

Figure S3: ^{13}C NMR Spectrum of **2** (DMSO-d_6, 298 K, 151 MHz)

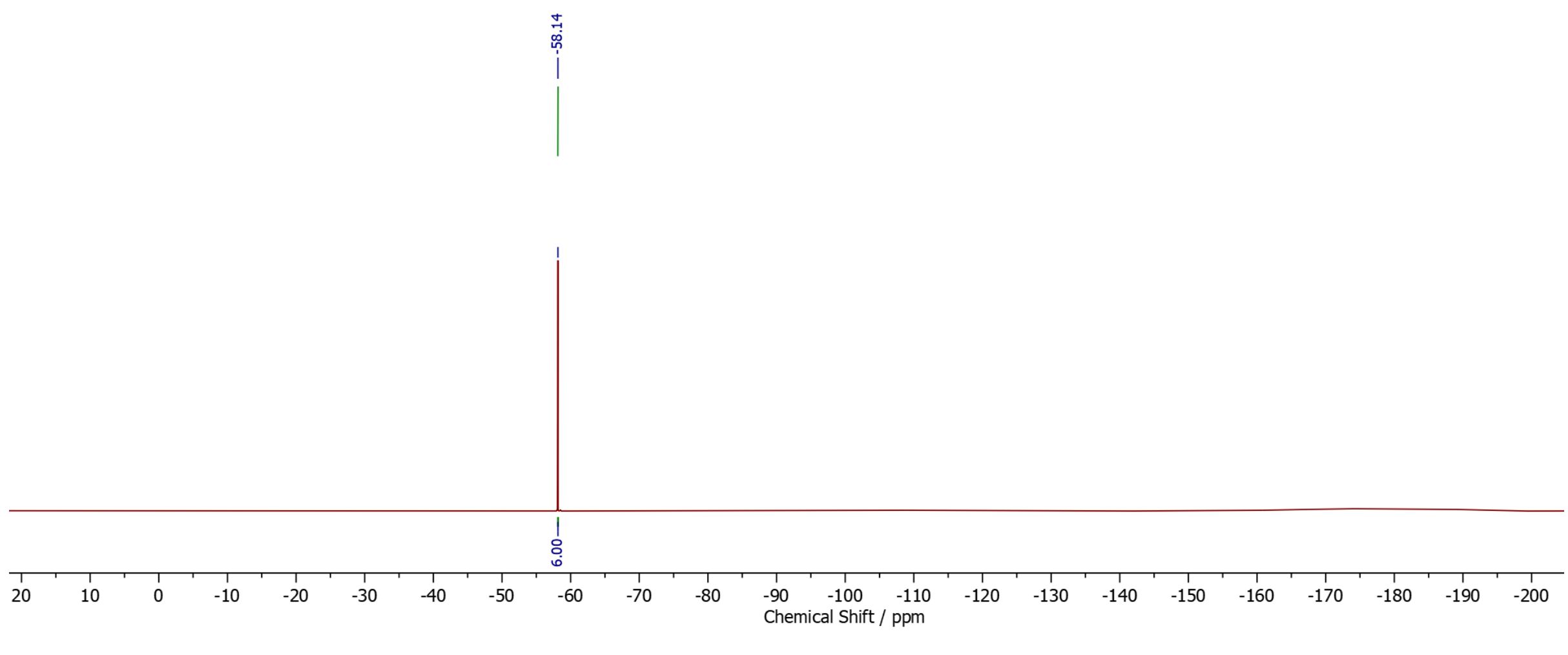

Figure S4: ^{19}F NMR Spectrum of **2** (DMSO-d_6, 298 K, 565 MHz)

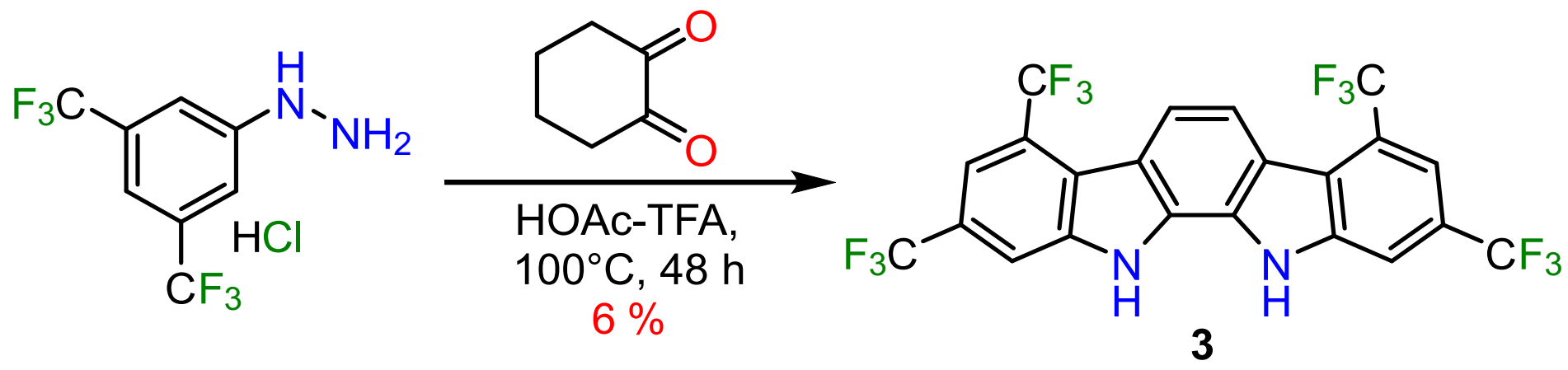

Scheme S3: Synthesis of 2,4,7,9-(trifluoromethyl)-indolo[2,3-α]carbazole **3**

Transporter 3. Prepared by a modified procedure.[3, 4] 1,2-Cyclohexanedione (380.0 mg, 3.39 mmol, 1 equiv.) and 3,5-trifluoromethyl phenylhydrazine hydrochloride (2.85 g, 10.2 mmol, 3 equiv.) were dissolved in HOAc (15 mL) and TFA (5 mL). The solution was heated to 100°C for 48 h, and then cooled to rt. The reaction mixture was reduced *in vacuo*. The residue was then dissolved in EtOAc and the organic layer was washed with H_2O (3 x 50 mL). EtOAc was used to extract any organics from the combined aqueous layers. The organic layers were combined and dried over $MgSO_4$, filtered and concentrated *in vacuo.* The crude product was dry-loaded onto silica and purified by silica flash column chromatography first in DCM. This removed the majority of impurities but one impurity remained in the collected fractions with the product, which was then dry-loaded onto silica again and re-purified in hexane: ethyl acetate 7:3 graded to 6:4. The solvent was evaporated *in vacuo* to yield the product as a light brown-beige crystalline solid (106.4 mg, 0.201 mmol, 6 %). ^{1}H NMR (600 MHz, DMSO-d_6) δ 12.32 (s, 2H), 8.56 (s, 2H), 8.14 (s, 2H), 7.86 (s, 2H). ^{13}C NMR (151 MHz, DMSO-d_6) δ 139.3, 127.9, 124.9 (q), 124.5 (q), 123.1 (q), 121.7 (q), 121.0, 117.1, 115.0 (q), 114.0 (q), 112.8 (q). ^{19}F NMR (565 MHz, DMSO-d_6) δ -59.62, -60.96. HRMS-ESI (m/z) calculated for $[C_{22}H_7F_{12}N_2]^-$, $[M-H]^-$: 527.0423, found: 527.0435

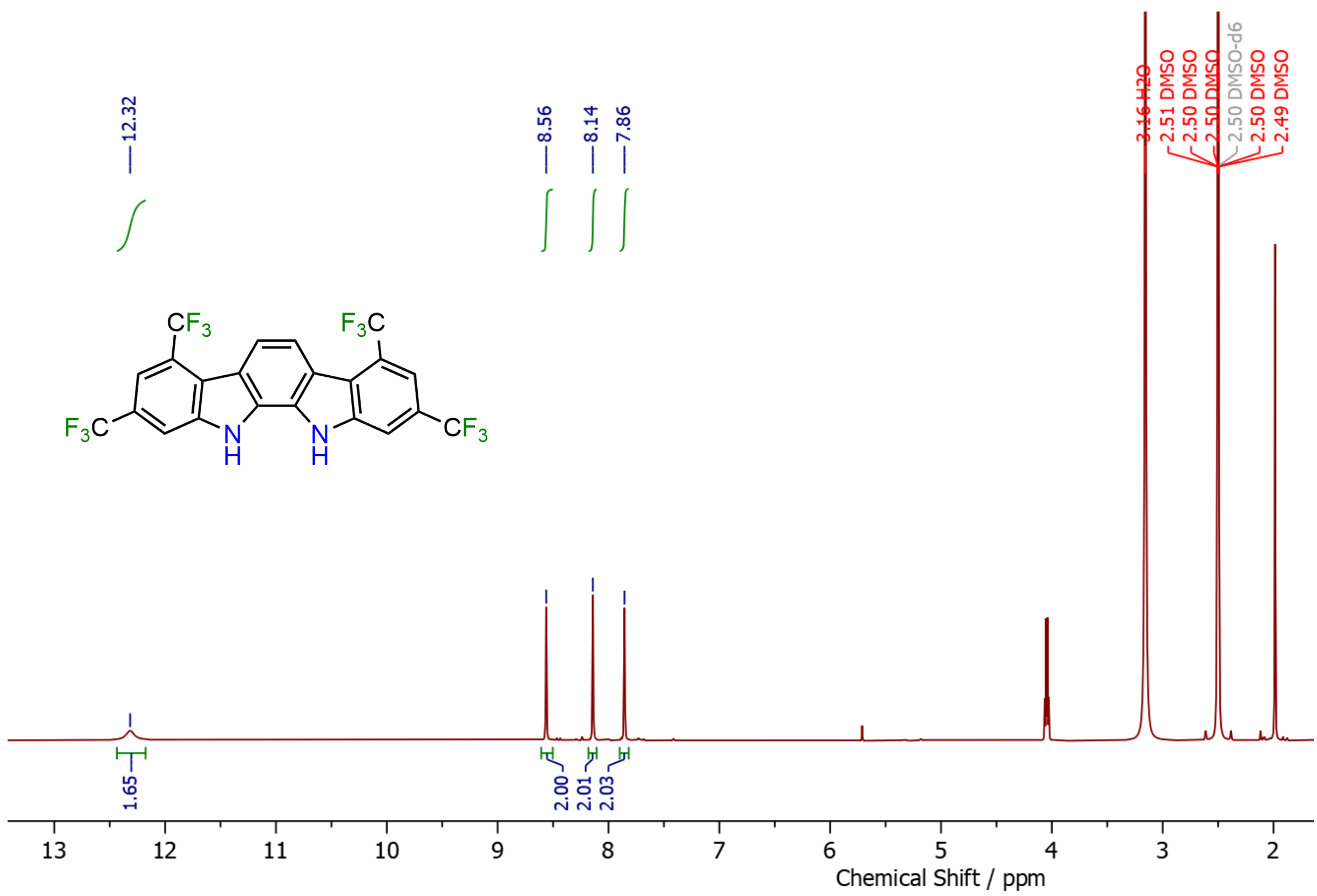

Figure S5: ^{1}H NMR Spectrum of **3** (DMSO-d_6, 333 K, 600 MHz)

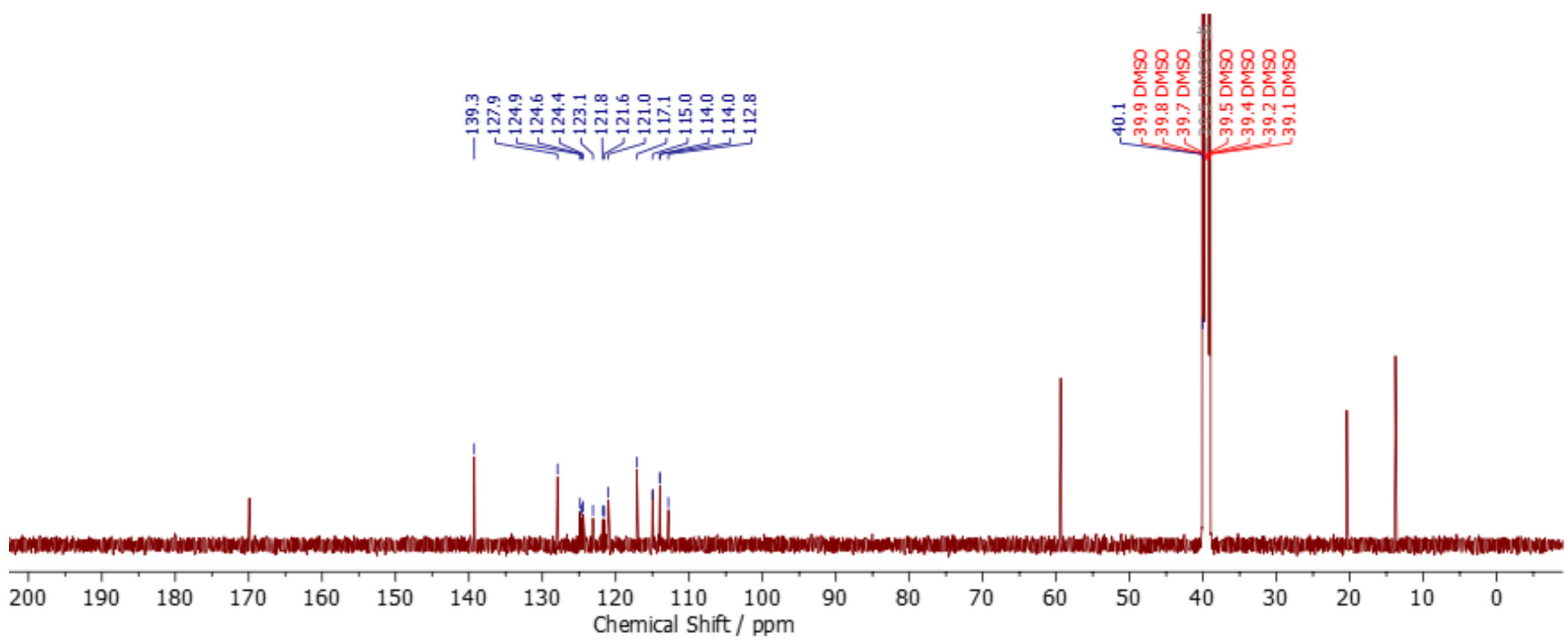

Figure S6: ^{13}C NMR Spectrum of **3** (DMSO-d_6, 333 K, 151 MHz)

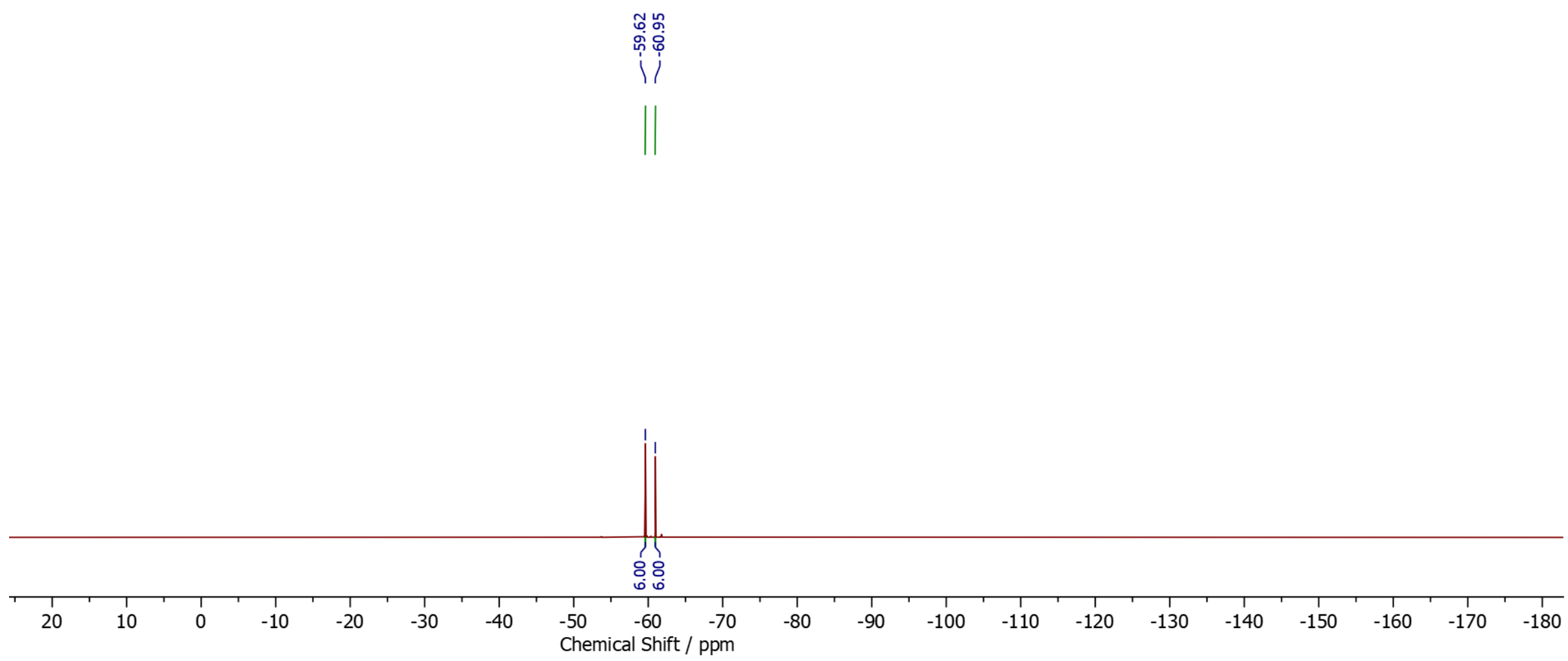

Figure S7: ^{19}F NMR Spectrum of **3** (DMSO-d_6, 333 K, 565 MHz)

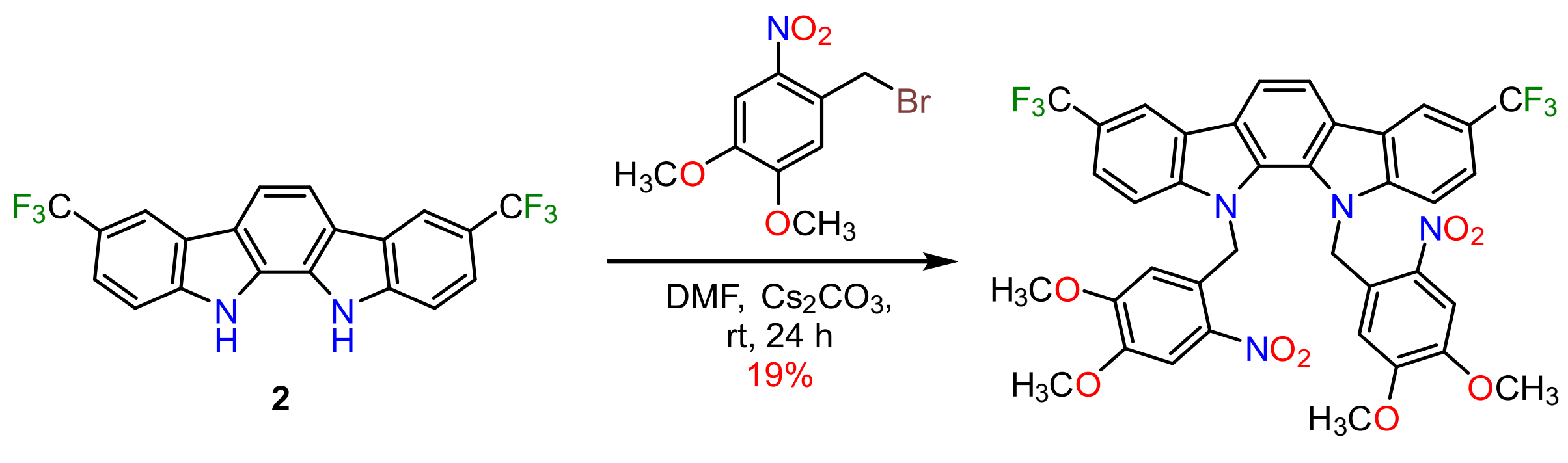

Scheme S4: Synthesis of double-caged dimethoxy-*o*-nitrobenzyl 3,8-(trifluoromethyl)-indolo[2,3-α]carbazole **4**

Double-caged transporter 4. Transporter **2** (20.4 mg, 0.05 mmol, 1 equiv.), dimethoxy-*o*-nitrobenzyl bromide **I** (43.1 mg, 0.16 mmol, 3 equiv.), and Cs_2CO_3 (50.8 mg, 0.16 mmol, 3 equiv.) were dissolved in dry DMF (2 mL) and left to react at rt for 24 h. The reaction mixture was washed with 5 % LiCl (3 x 10 mL) and the organic layer was extracted with EtOAc (3 x 5 mL). The organic layers were combined, washed with H_2O (50 mL), and then dried over $MgSO_4$, filtered and concentrated *in vacuo.* The crude product was dry-loaded onto silica and purified by silica gel auto-column chromatography in hexane: ethyl acetate 7:3. The solvent was evaporated *in vacuo* to yield the product as a yellow-orange crystalline solid (7.7 mg, 0.01 mmol, 19 %). ^1H NMR (600 MHz, DMSO-d_6) δ 8.79 (s, 2H), 8.46 (s, 2H), 7.70 (s, 2H), 7.67 (dd, *J* = 8.7, 1.8 Hz, 2H), 7.39 (d, *J* = 8.6 Hz, 2H), 6.85 (s, 2H), 5.71 (s, 4H), 3.91 (s, 6H), 3.44 (s, 6H). ^{13}C NMR (151 MHz, DMSO-d_6) δ 154.2, 148.0, 144.7, 137.8, 130.2, 127.9 – 122.5 (q), 127.4, 124.6, 124.2, 122.6 (q), 121.9 - 121.3 (q), 117.8 - 117.7 (q), 114.7, 112.0, 109.4, 108.6, 56.2, 56.0, 51.3.. ^{19}F NMR (565 MHz, DMSO-d_6) δ -58.52. HRMS-ESI (m/z) calculated for $[C_{38}H_{28}F_6N_4O_8K]^+$, $[M+K]^+$: 821.1443, found: 821.1427

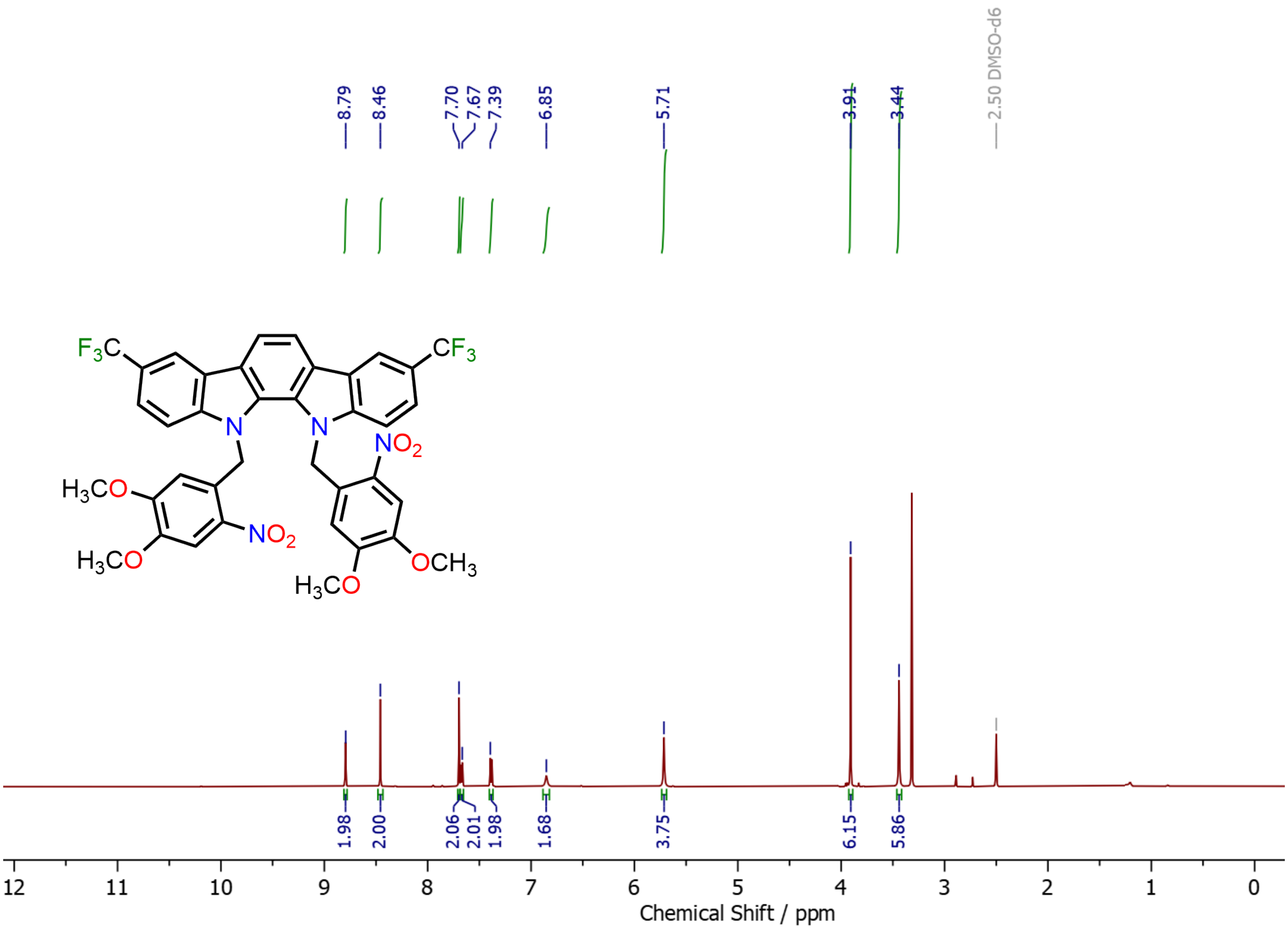

Figure S8: ^{1}H NMR Spectrum of **4** (DMSO-d_6, 298 K, 600 MHz)

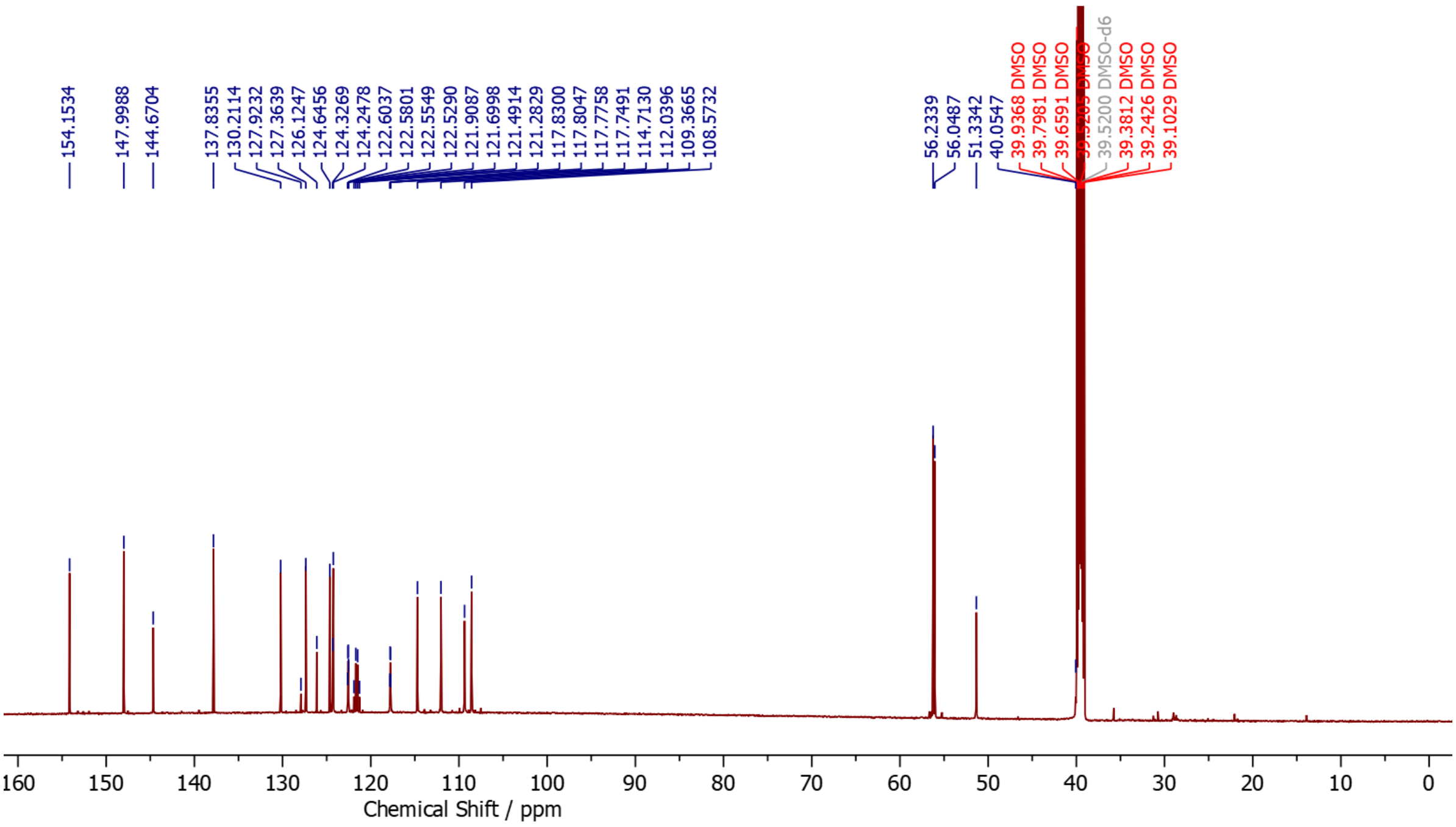

Figure S9: ^{13}C NMR Spectrum of **4** (DMSO-d_6, 298 K, 151 MHz)

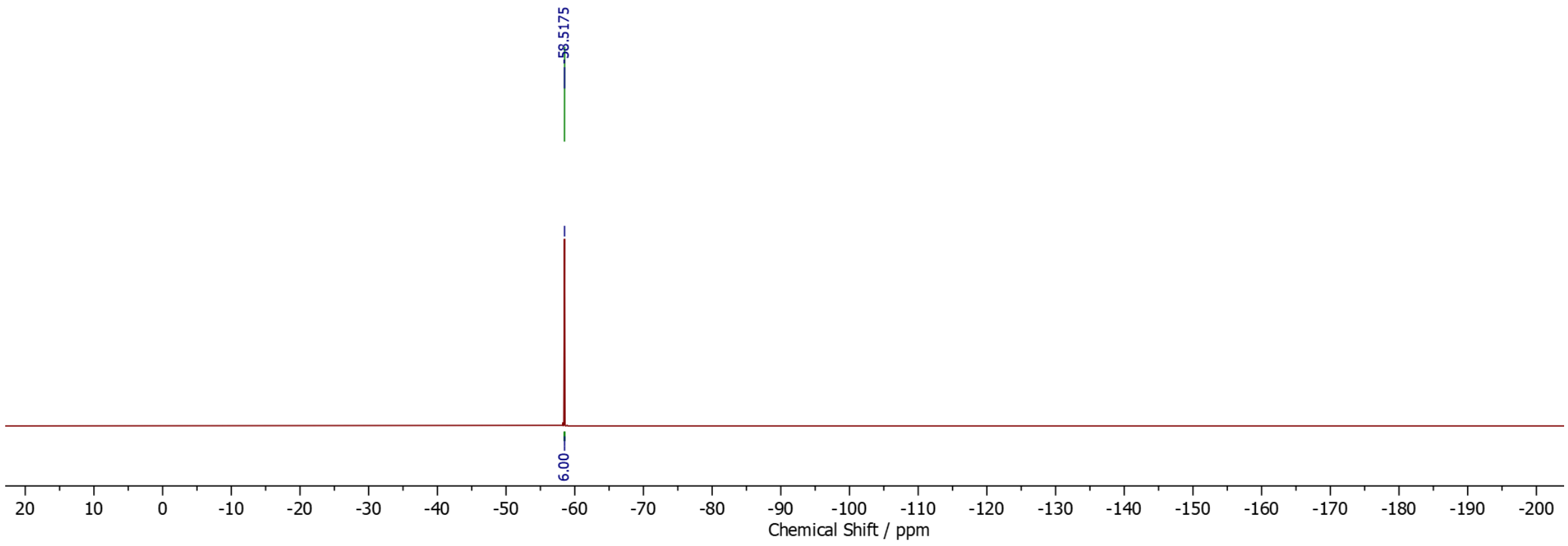

Figure S10: ^{19}F NMR Spectrum of **4** (DMSO-d_6, 298 K, 565 MHz)

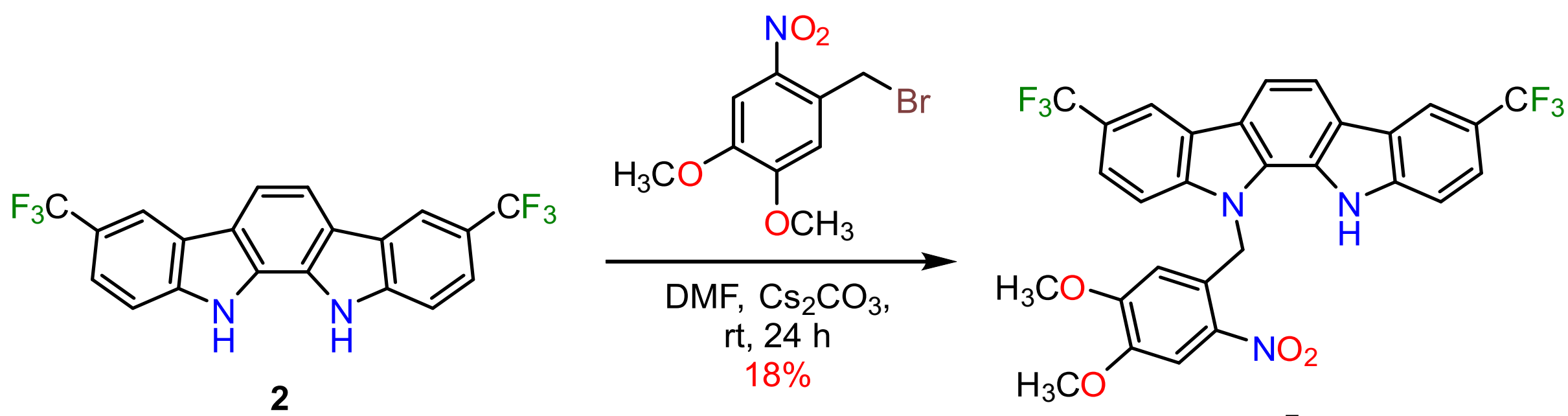

Scheme S5: Synthesis of mono-caged dimethoxy-*o*-nitrobenzyl 3,8-(trifluoromethyl)-indolo[2,3-α]carbazole **5**

Mono-caged transporter 5. Transporter **2** (30.5 mg, 0.08 mmol, 1 equiv.), dimethoxy-*o*-nitrobenzyl bromide **I** (21.1 mg, 0.08 mmol, 1 equiv.), and Cs_2CO_3 (37.4 mg, 0.12 mmol, 1.5 equiv.) were dissolved in dry DMF (2 mL) and left to react at rt for 24 h. The reaction mixture was washed with 5% LiCl (3 x 10 mL) and the organic layer was extracted with EtOAc (3 x 5 mL). The organic layers were combined, washed with H_2O (50 mL), and then dried over $MgSO_4$, filtered and concentrated *in vacuo.* The crude product was dry-loaded onto silica and purified by silica flash column chromatography in hexane: ethyl acetate 8:2 graded to 6:4. The solvent was evaporated *in vacuo* to yield the product as a pale yellow crystalline solid (8.1 mg, 0.01 mmol, 18 %). ^{1}H NMR (600 MHz, DMSO-d_6) δ 11.88 (s, 1H), 8.75 (s, 1H), 8.65 (s, 1H), 8.30 (d, J = 8.3 Hz, 1H), 8.28 (d, J = 8.3 Hz, 1H), 7.84 (s, 1H), 7.77 (d, J = 8.6 Hz, 1H), 7.71 (d, J = 2.0 Hz, 1H), 7.70 (d, J = 4.0 Hz, 1H), 7.68 (dd, J = 8.6, 1.9 Hz, 1H), 6.49 (s, 2H), 5.62 (s, 1H), 3.82 (s, 3H), 2.89 (s, 3H). ^{13}C NMR (151 MHz, DMSO-d_6) δ 153.4, 147.7, 141.7, 141.6, 139.7, 128.6, 128.3 – 122.9 (q), 128.2 – 122.8 (q), 127.9, 125.8, 123.5, 122.8, 122.3, 122.1 (q), 121.8 (q), 121.2 – 120.6 (q), 120.5 - 119.8 (q), 118.0 (q), 117.8 (q), 114.1, 113.4, 112.4, 110.9, 109.1, 108.4, 56.3, 55.4, 46.8, 40.1.. ^{19}F NMR (565 MHz, DMSO-d_6) δ -58.27, -58.35. HRMS-ESI (m/z) calculated for $[C_{29}H_{20}F_6N_3O_4]^+$, $[M+H]^+$: 588.1353, found: 588.1351

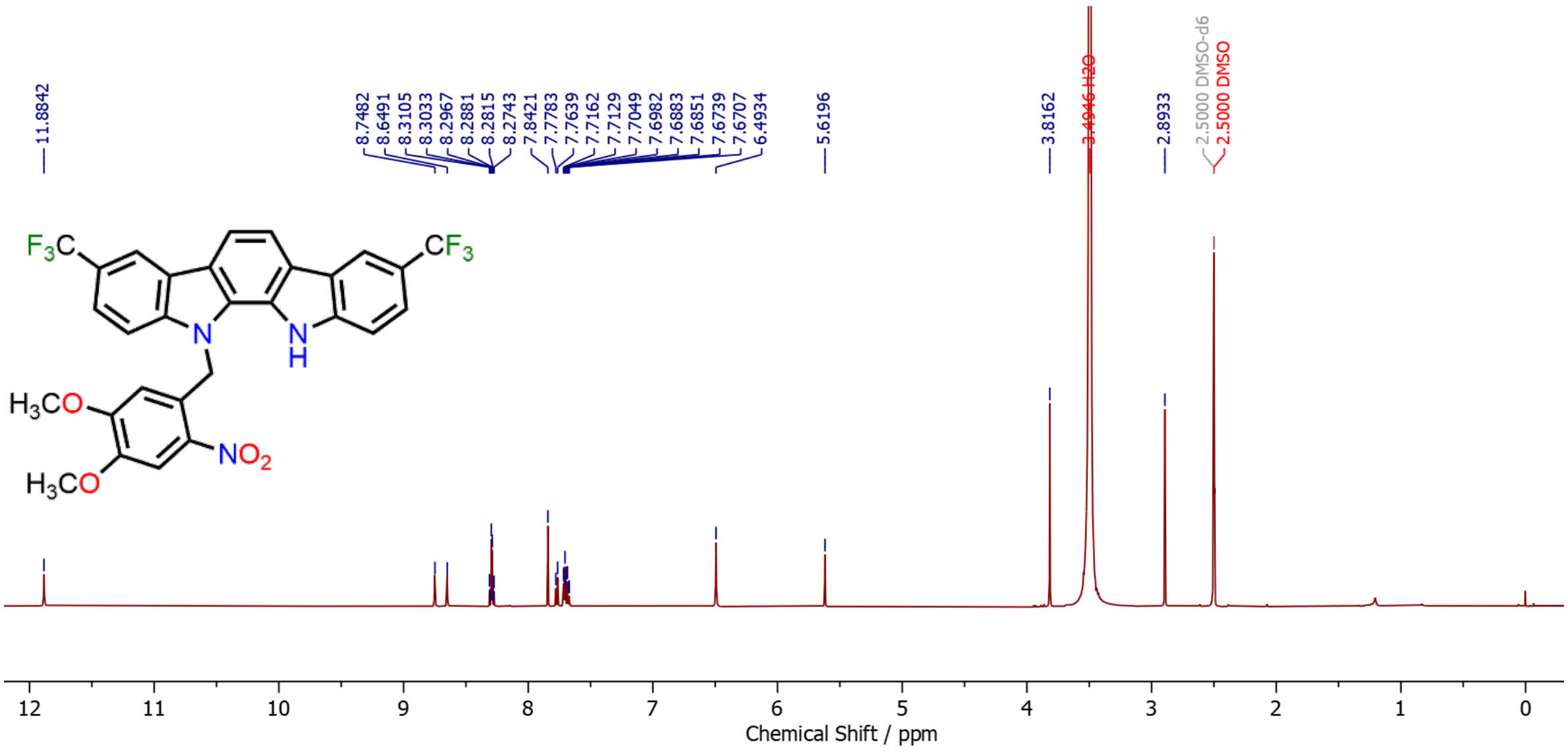

Figure S11: ^{1}H NMR Spectrum of **5** (DMSO-d_6, 298 K, 600 MHz)

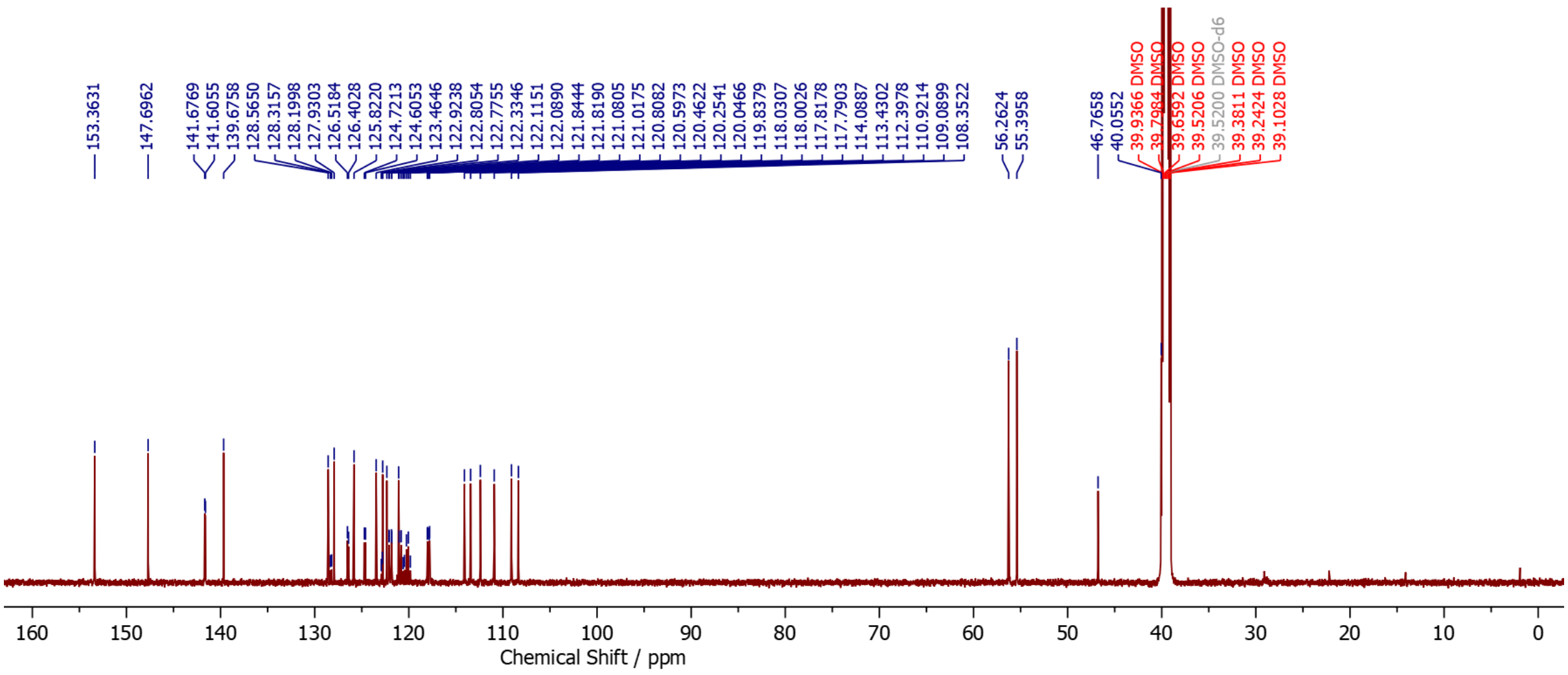

Figure S12: ^{13}C NMR Spectrum of **5** (DMSO-d_6, 298 K, 151 MHz)

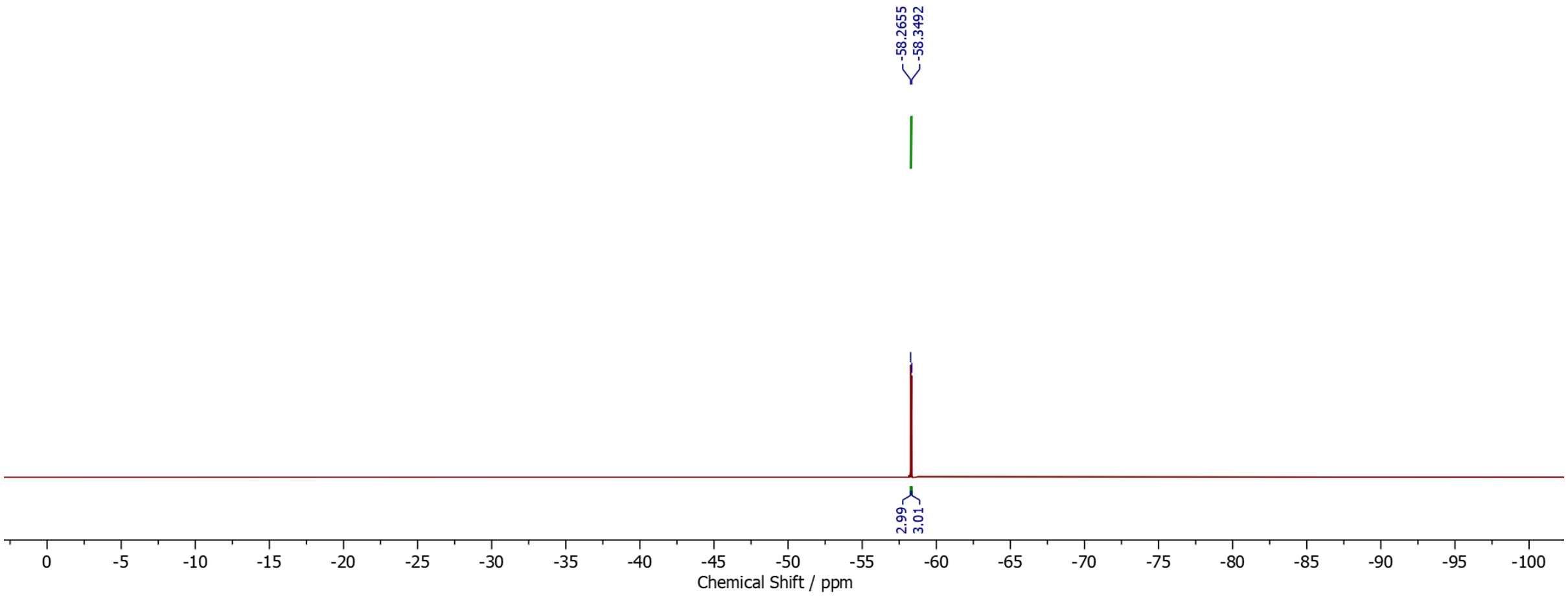

Figure S13: ^{19}F NMR Spectrum of **5** (DMSO-d_6, 298 K, 565 MHz)

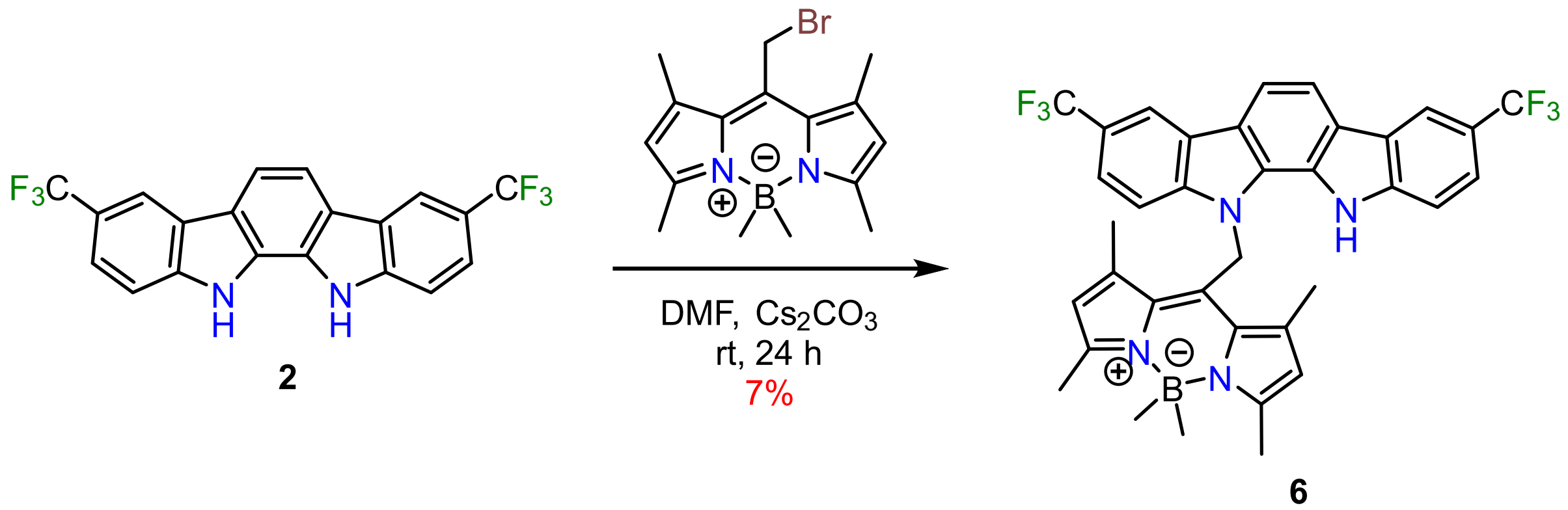

Scheme S14: Synthesis of mono-caged BODIPY 3,8-(trifluoromethyl)-indolo[2,3-α]carbazole **6**

Mono-caged transporter 6. Transporter **2** (84.9 mg, 0.22 mmol, 1 equiv.), boron-dipyrromethene bromide **II** (64.8 mg, 0.19 mmol, 0.9 equiv.), and Cs_2CO_3 (211.4 mg, 0.65 mmol, 3 equiv.) were dissolved in dry DMF (6 mL) and left to react at rt for 24 h. The reaction mixture was washed with 5% LiCl (3 x 10 mL) and the organic layer was extracted with EtOAc (3 x 5 mL). The organic layers were combined, washed with H_2O (50 mL), and then dried over $MgSO_4$, filtered and concentrated *in vacuo.* The crude product was dry-loaded onto silica and purified by silica flash column chromatography in hexane: ethyl acetate 9:1. The solvent was evaporated *in vacuo* to yield the product as a red-orange crystalline solid (9.4 mg, 0.01 mmol, 7 %). ^{1}H NMR (600 MHz, DMSO-d_6) δ 12.15 (s, 1H), 8.71 (d, J = 16.1 Hz, 2H), 8.33 (d, J = 8.4 Hz, 1H), 8.28 (d, J = 8.2 Hz, 1H), 7.83 (d, J = 8.4 Hz, 1H), 7.76 (dd, J = 8.5, 1.9 Hz, 1H), 7.43 (d, J = 10.9 Hz, 1H), 7.39 (d, J = 9.0 Hz, 1H), 6.43 (s, 2H), 6.22 (s, 2H), 2.52 (s, 6H), 1.97 (s, 6H), 0.33 (s, 3H), 0.25 (s, 3H). ^{13}C NMR (151 MHz, Acetone-d_6) δ 154.9, 142.6, 141.4, 138.8, 133.5, 133.1, 129.7, 127.6, 127.4, 127.2 – 122.1 (q), 125.6, 125.4, 124.2, 123.8, 122.8 (q), 122.4 - 122.3 (q), 122.3, 122.2, 122.0, 121.8, 118.6 - 118.5 (q), 114.9, 114.0, 113.0, 112.2, 47.0, 30.3, 16.8, 15.7. ^{19}F NMR (565 MHz, DMSO-d_6) δ -58.18, -58.62. HRMS-ESI (m/z) calculated for $[C_{36}H_{32}BF_6N_4]^+$, $[M+H]^+$: 645.2619, found: 645.2628

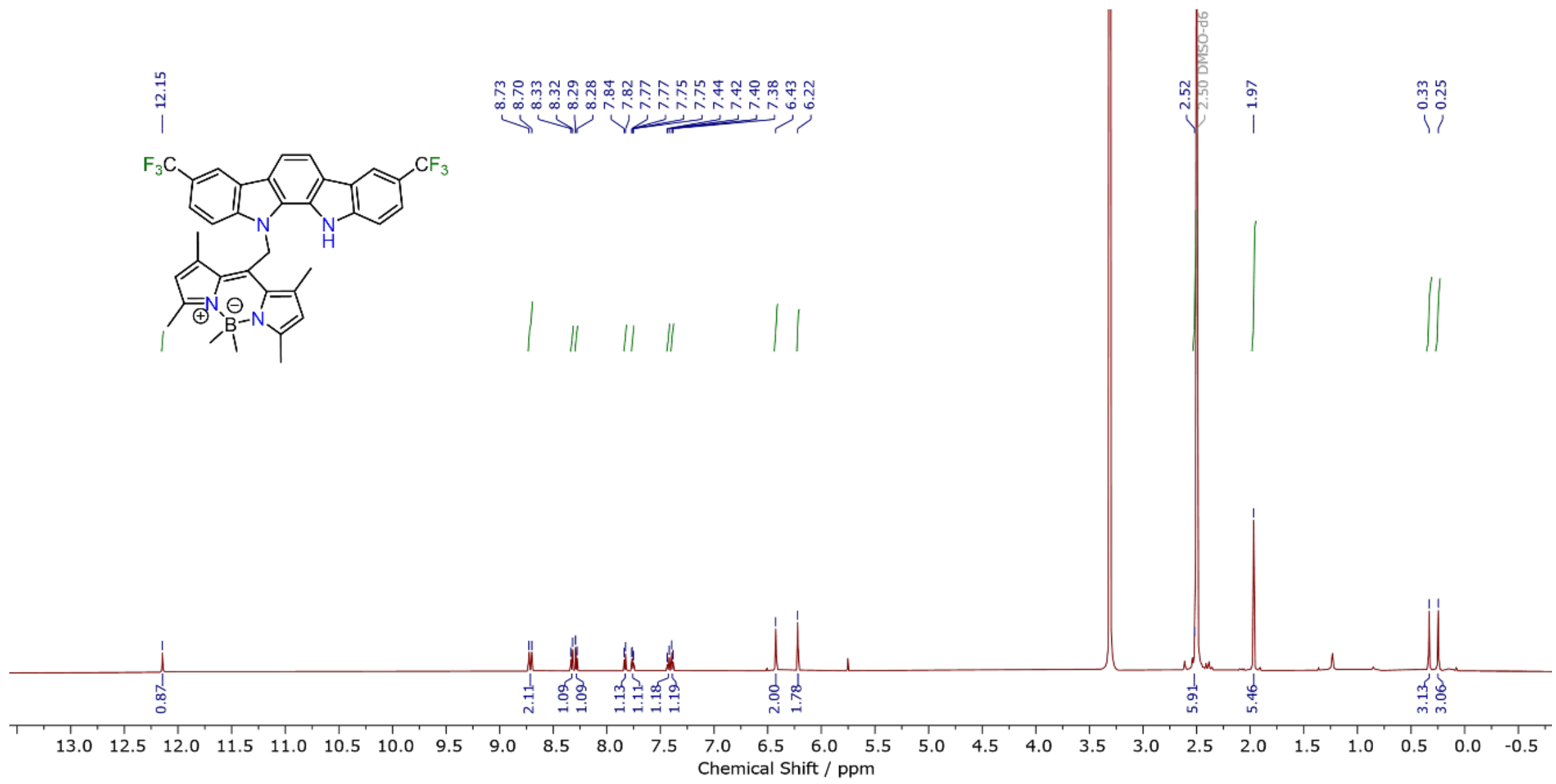

Figure S15: ^{1}H NMR Spectrum of **6** (DMSO-d_6, 298 K, 600 MHz)

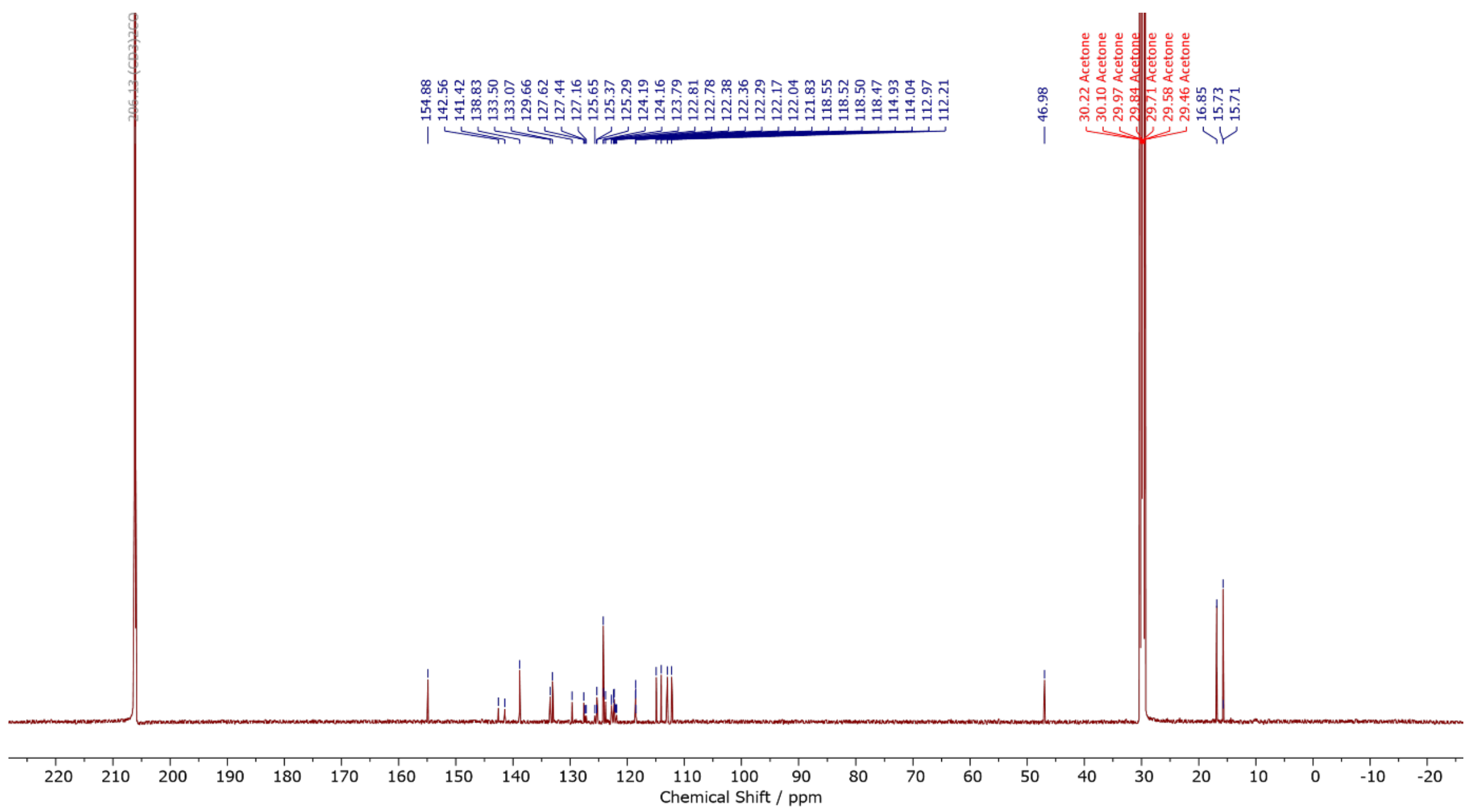

Figure S16: ^{13}C NMR Spectrum of **6** (Acetone-d_6, 298 K, 151 MHz)

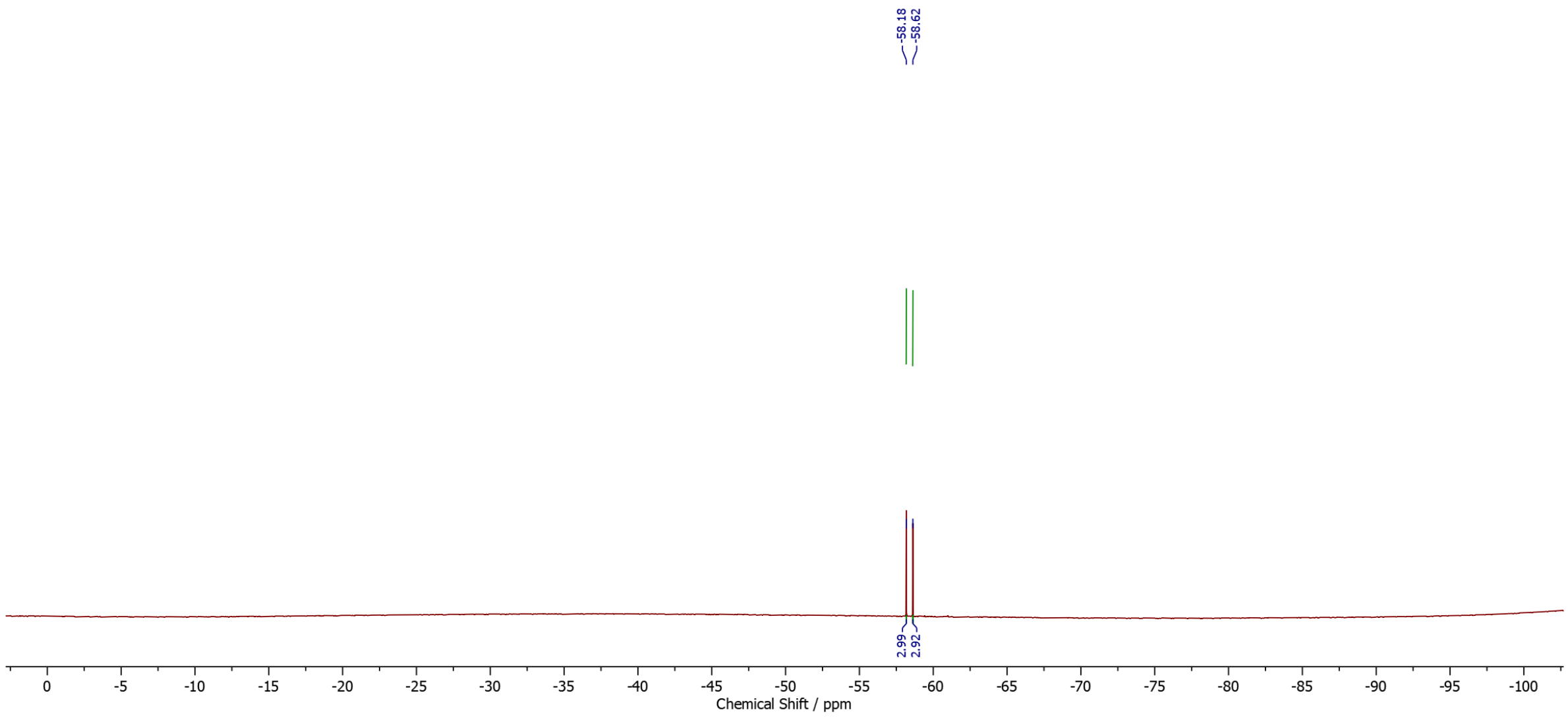

Figure S17: ^{19}F NMR Spectrum of **6** (DMSO-d_6, 298 K, 565 MHz)

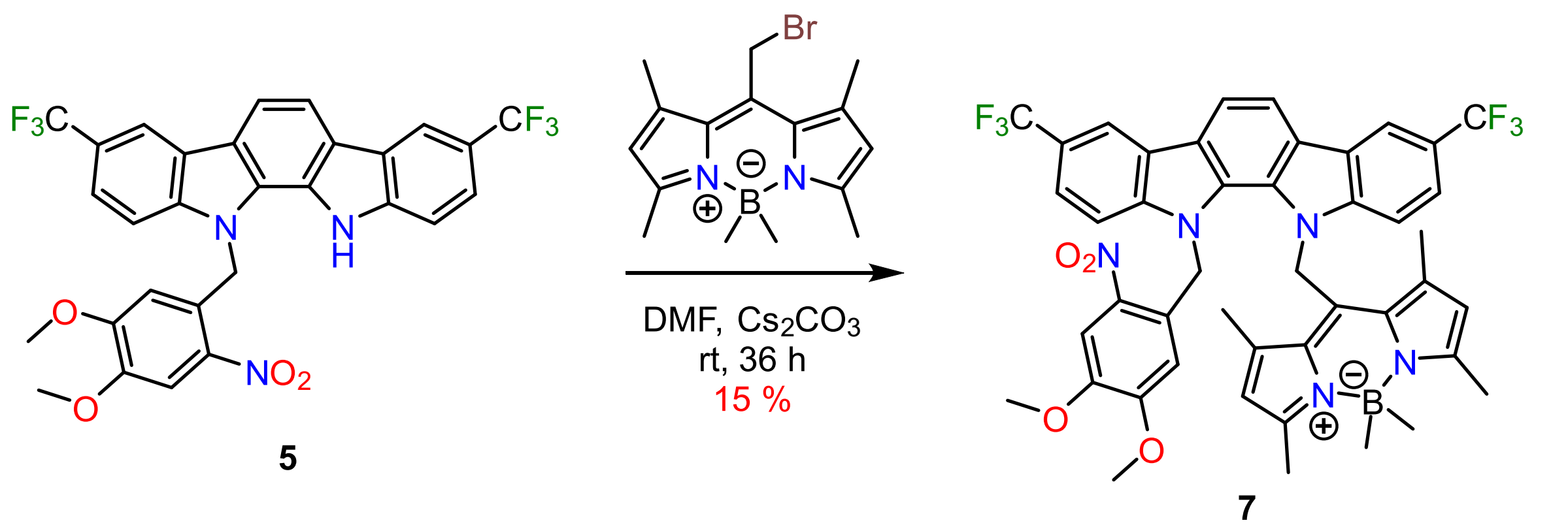

Scheme S7: Synthesis of dual-caged dimethoxy-*o*-nitrobenzyl BODIPY 3,8-(trifluoromethyl)-indolo[2,3-α]carbazole **7**

Dual-caged transporter 7. Transporter **5** (35.9 mg, 0.06 mmol, 1 equiv.), boron-dipyrromethene bromide **II** (30.5 mg, 0.09 mmol, 1.5 equiv.), Cs_2CO_3 (79.6 mg, 0.24 mmol, 4 equiv.), and NaI (13.7 mg, 0.09 mmol, 1.5 equiv.) were dissolved in dry DMF (3 mL) and left to react at rt for 16 h. The reaction mixture was washed with 5% LiCl (3 x 15 mL) and the organic layer was extracted with EtOAc (3 x 5 mL). The organic layers were combined and then dried over $MgSO_4$, filtered and concentrated *in vacuo.* The crude product was dry-loaded onto silica and purified by silica flash column chromatography in hexane: ethyl acetate 9:1. The solvent was evaporated *in vacuo* to yield the product as a red-orange crystalline solid (6.7 mg, 0.01 mmol, 13 %). ^{1}H NMR (600 MHz, Acetone-d_6) δ 8.76 – 8.73 (m, 1H), 8.62 (d, *J* = 2.0 Hz, 1H), 8.45 (d, *J* = 8.3 Hz, 1H), 8.41 (d, *J* = 8.2 Hz, 1H), 7.89 (s, 1H), 7.75 (dd, *J* = 8.6, 1.9 Hz, 1H), 7.64 (d, *J* = 8.6 Hz, 1H), 7.49 (d, *J* = 8.9 Hz, 1H), 7.40 (dd, *J* = 8.9, 1.9 Hz, 1H), 6.96 (s, 1H), 6.44 (s, 2H), 6.12 (d, *J* = 1.1 Hz, 1H), 6.08 (s, 2H), 6.05 (d, *J* = 1.1 Hz, 1H), 4.92 (d, *J* = 5.1 Hz, 1H), 4.38 (t, *J* = 5.2 Hz, 1H), 3.91 (s, 3H), 3.45 (s, 3H), 2.53 (s, 3H), 2.43 (s, 3H), 2.42 (s, 3H), 1.86 (s, 3H), 0.16 (s, 6H). ^{13}C NMR (151 MHz, Acetone-d_6) δ 206.1, 155.6, 154.5, 152.7, 149.6, 146.4, 142.9, 141.2 - 131.9 (q), 140.5 - 132.3 (q), 131.6, 128.8, 127.2 - 123.9 (q), 127.0 - 125.4 (q), 126.1, 125.2, 124.3, 123.7 (q), 123.0, 122.9, 122.8, 122.8 - 122.7 (q), 122.6, 118.6 - 118.5 (q), 118.5 - 118.4 (q), 115.7, 115.6, 113.6, 113.2, 110.4, 110.2, 56.7, 56.6, 56.2, 53.4, 49.0, 30.2, 30.1, 30.0, 29.7, 29.6, 29.5, 16.8, 16.6, 16.0, 15.9. ^{19}F NMR (565 MHz, Acetone-d_6) δ -60.75, -60.94. HRMS-ESI (m/z) calculated for $[C_{45}H_{41}BF_6N_5O_4]^+$, $[M+H]^+$: 840.3172, found: 840.3150

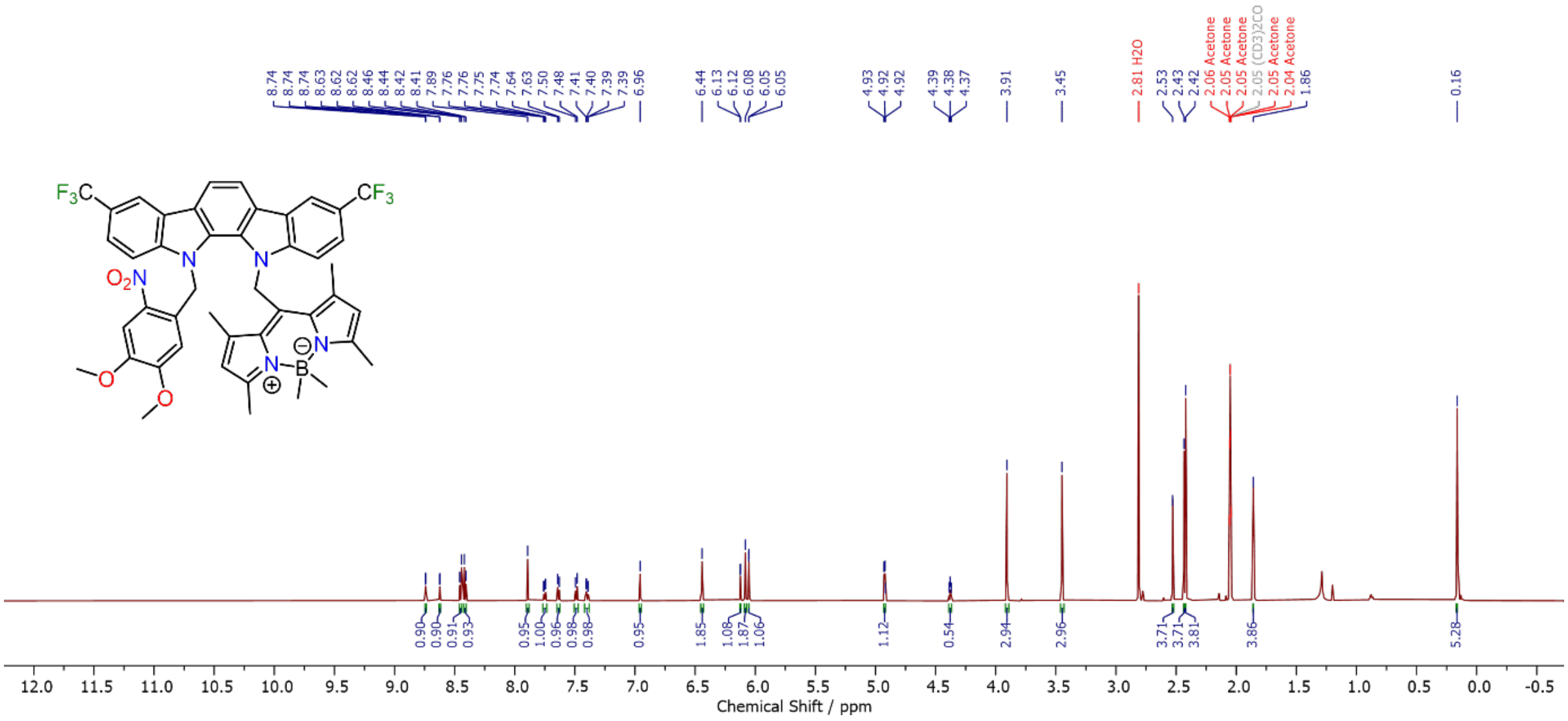

Figure S18: ^{1}H NMR Spectrum of **7** (Acetone-d_6, 298 K, 600 MHz)

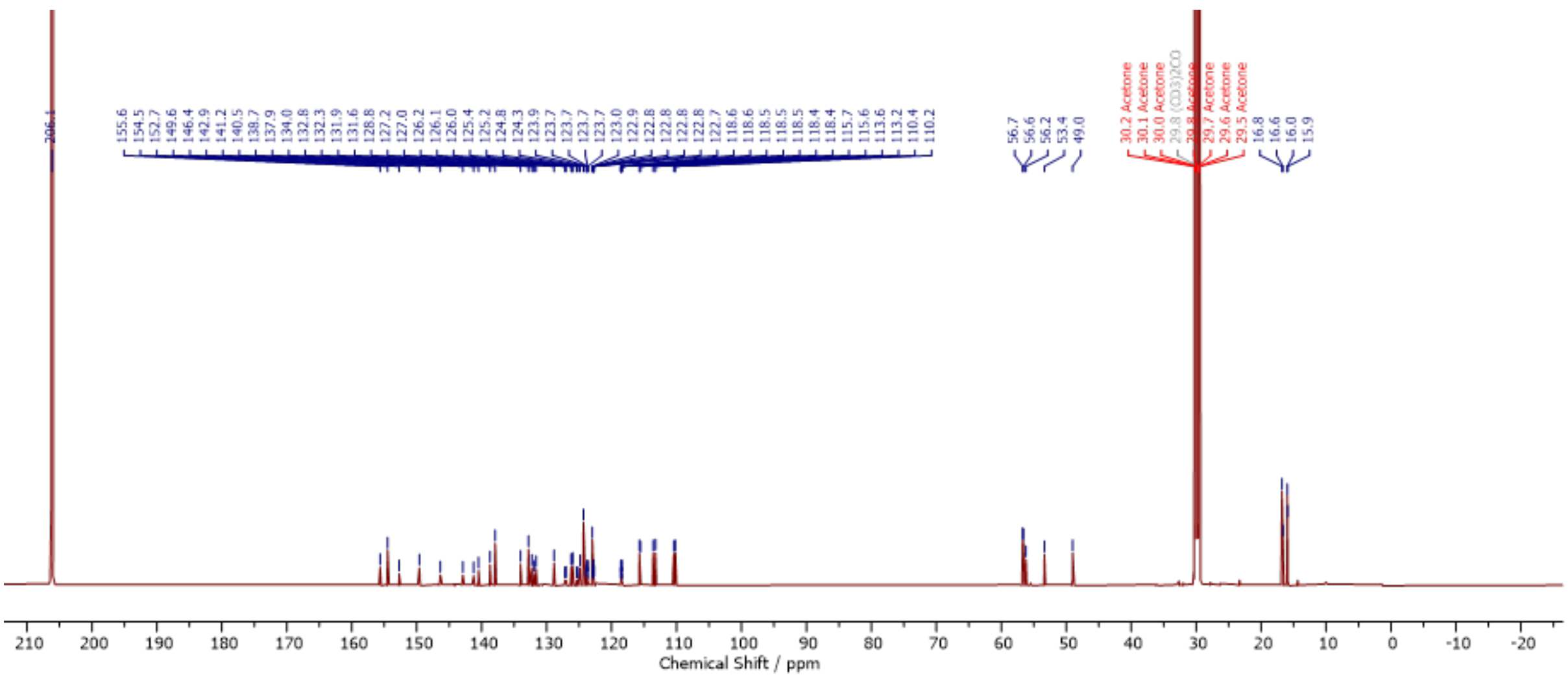

Figure S19: ^{13}C NMR Spectrum of **7** (Acetone-d_6, 298 K, 151 MHz)

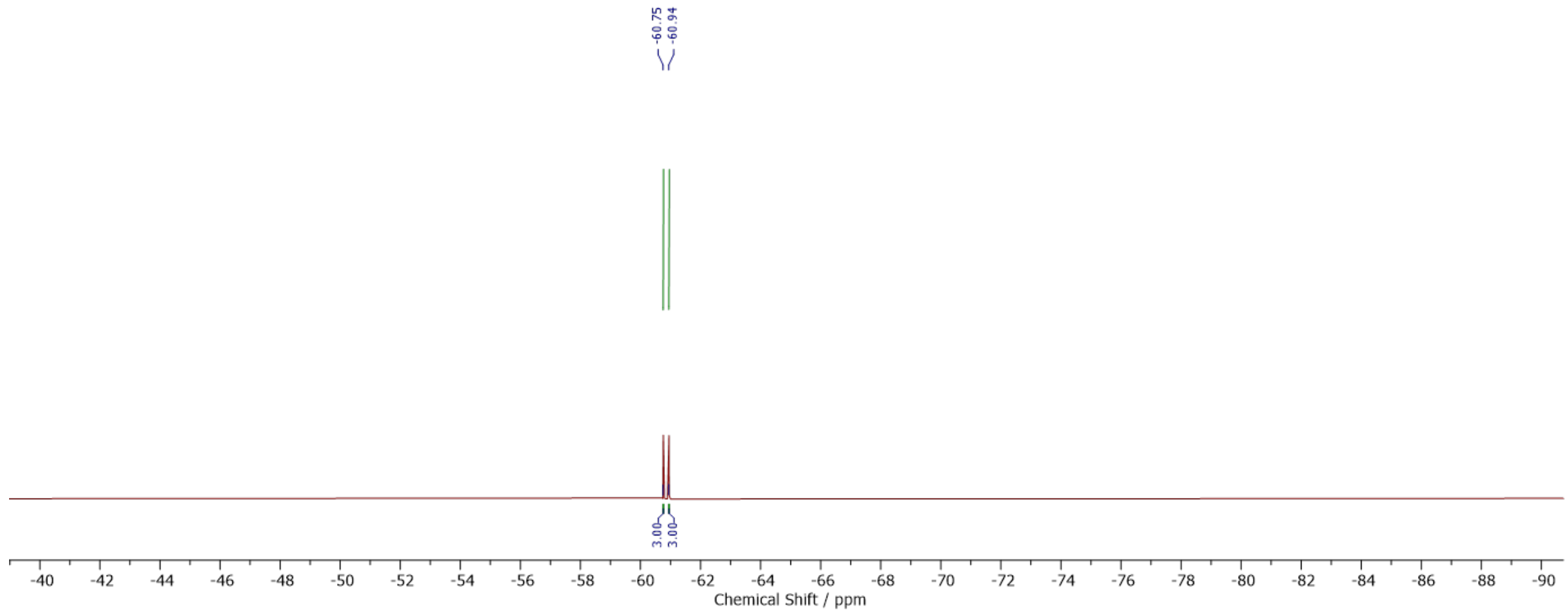

Figure S20: ^{19}F NMR Spectrum of **7** (Acetone-d_6, 298 K, 565 MHz)

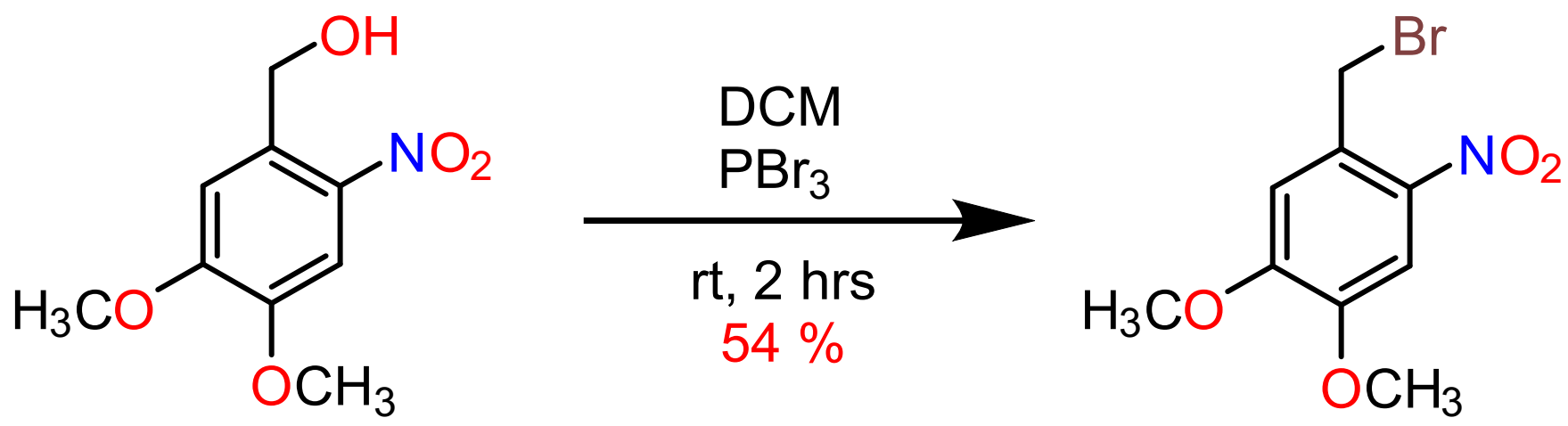

I

Scheme S8: Synthesis of 4,5-dimethoxy-2-nitrobenzyl bromide

4,5-Dimethoxy-2-nitrobenzyl bromide (I): Prepared by literature procedure.[5, 6] Under a N_2 atmosphere, 4,5-dimethoxy-2-nitrobenzyl alcohol (2.50 g, 11.7 mmol, 1 equiv.) was dissolved in dry DCM (10 mL) at 0°C. PBr_3 (1.23 mL, 12.9 mmol, 1.1 equiv.) was added dropwise *via* syringe to the stirred solution. Stirring was continued for 2 h at rt. The reaction mixture was neutralised by saturated $NaHCO_3$ (100 mL) and washed with brine (50 mL). The organic layer was extracted with DCM (3 x 15 mL). The organic layers were combined and dried over $MgSO_4$, filtered and concentrated *in vacuo.* The crude product was dry-loaded onto silica and purified by silica flash column chromatography in hexane: ethyl acetate 7:3. The solvent was evaporated *in vacuo* to yield the product as a yellow crystalline solid (1.75 g, 6.35 mmol, 54 %). ^1H NMR (400 MHz, $CDCl_3$) δ 7.67 (s, 1H), 6.94 (s, 1H), 4.87 (s, 2H), 4.00 (s, 3H), 3.96 (s, 3H). Data consistent with the literature.[5]

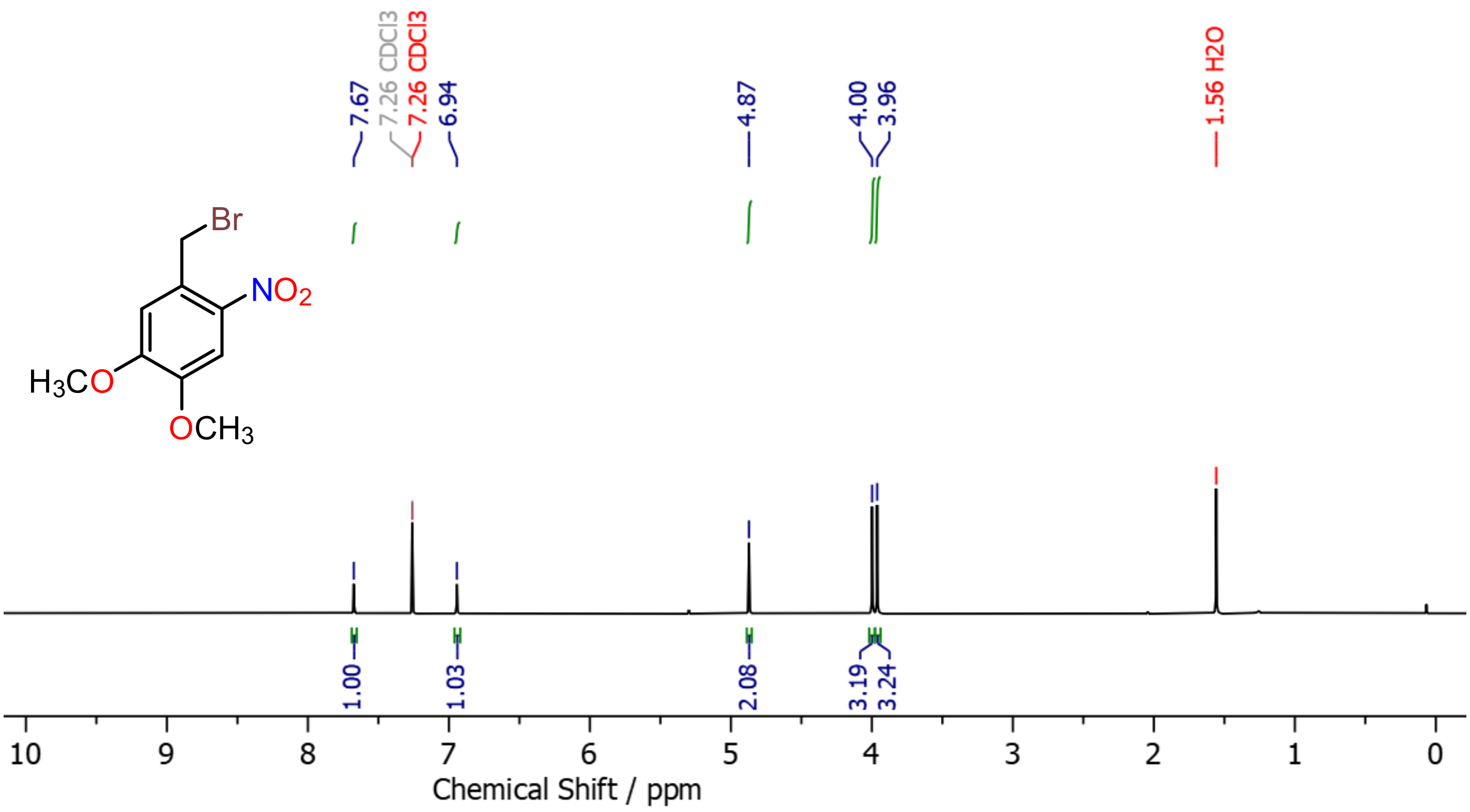

Figure S21: ^1H NMR Spectrum of **I** ($CDCl_3$, 298 K, 400 MHz)

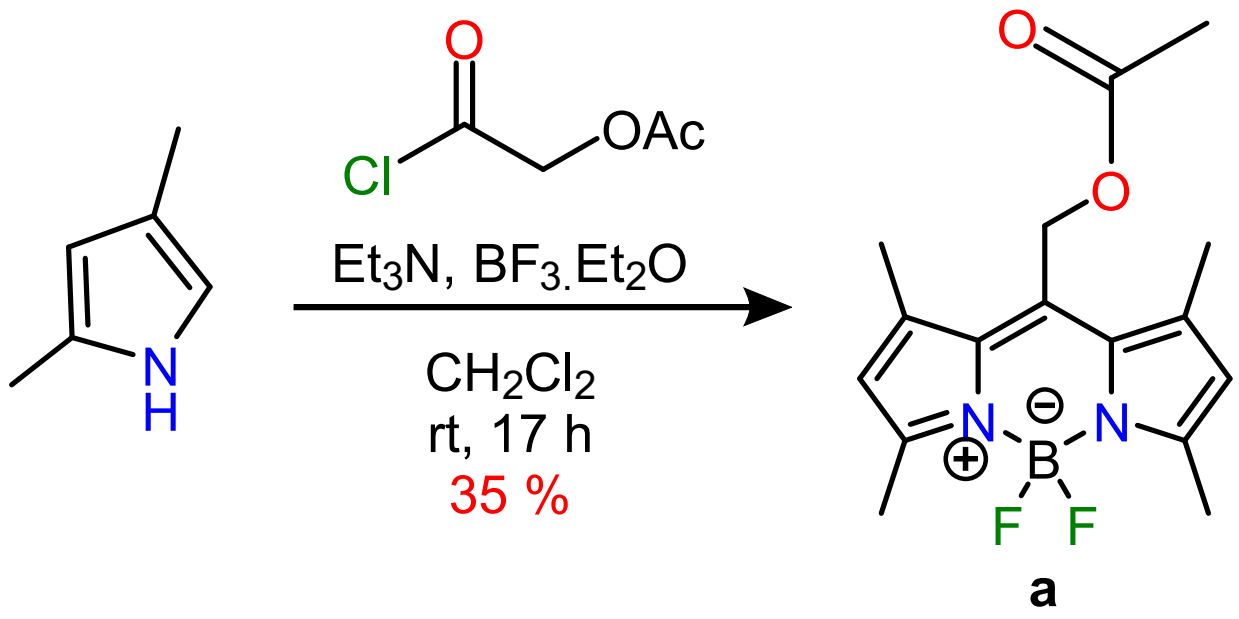

Scheme S9: Synthesis of 8-Acetoxymethyl-1,3,5,7-tetramethyl pyrromethene fluoroborate

8-Acetoxymethyl-1,3,5,7-tetramethyl pyrromethene fluoroborate (a): Prepared by literature procedure.[7] 2-Chloro-2-oxoethyl acetate (1.72 mL, 16.0 mmol, 1.2 equiv.) was added to a solution of 2,4-dimethylpyrrole (2.54 g, 26.7 mmol, 2 equiv.) in dry DCM (125 mL) under a nitrogen atmosphere. The reaction mixture was stirred in the dark at rt for 17 h. After this time, the flask was opened and TEA (11.13 mL, 79.84 mmol, 6 equiv.) was added. The resulting mixture was allowed to stir for 15 min. The flask was again put under a nitrogen atmosphere, and boron trifluoride diethyl etherate (14.78 mL, 119.8 mmol, 9 equiv.) was added. After 1 h, another portion of TEA (11.13 mL, 79.84 mmol, 6 equiv.) and boron trifluoride diethyl etherate (14.78 mL, 119.8 mmol, 9 equiv.) was added. Silica (100 g) was added to the flask, and the solvents were evaporated. Compound **(a)** was purified by silica gel flash-column chromatography using pentane:Et_2O 2:1 as the eluent. The product was obtained as red-gold crystals (1.49 g, 4.65 mmol, 35 %). ^1H NMR (400 MHz, $CDCl_3$) δ 6.09 (s, 2H), 5.30 (s, 2H), 2.53 (s, 6H), 2.36 (s, 6H), 2.13 (s, 3H). Data consistent with that given in the literature.[7, 8]

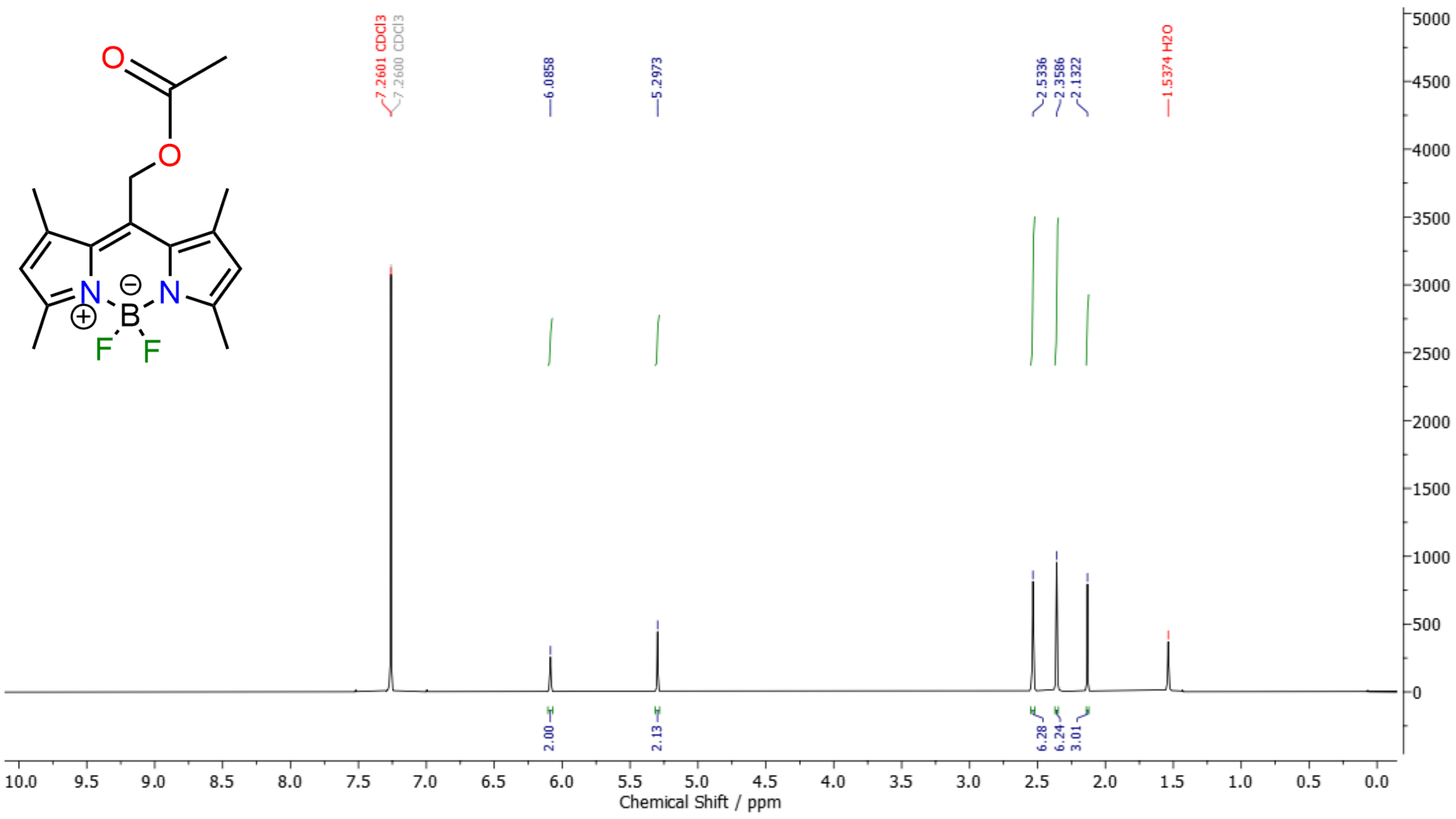

Figure S22: ^1H NMR Spectrum of **(a)** ($CDCl_3$, 298 K, 400 MHz)

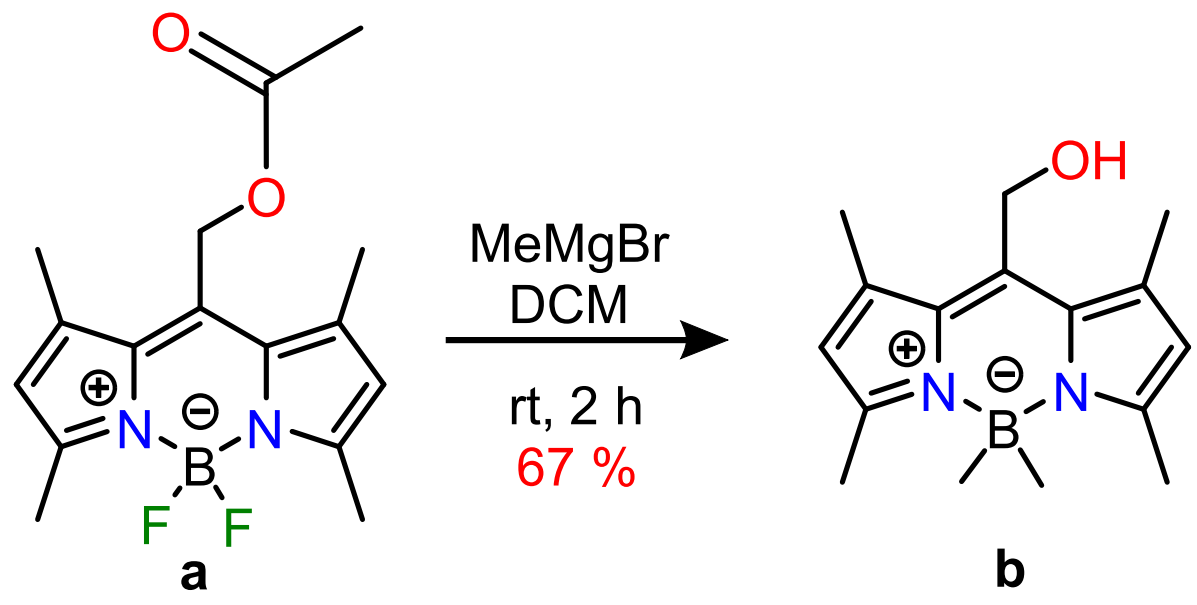

Scheme S10: Synthesis of 8-Hydroxymethyl-1,3,5,7-tetramethyl pyrromethene methylborate

8-Hydroxymethyl-1,3,5,7-tetramethyl pyrromethene methylborate: Prepared by literature procedure.[9] Methylmagnesium bromide in THF (1.08 mL, 9.34 mmol, 15 equiv.) was added to a solution of 8-Acetoxymethyl-1,3,5,7-tetramethyl pyrromethene fluoroborate **(a)** (200 mg, 0.62 mmol, 1 equiv.) in dry DCM (10 mL) under a nitrogen atmosphere. The reaction mixture was stirred in the dark at rt for 1 h, after which the reaction was complete by TLC. The reaction was quenched with NH_4Cl (100 mL). The organic layer was extracted with EtOAc (50 mL). The organic layer was washed once with NH_4Cl (60 mL) and with brine (2 x 60 mL), then dried over $MgSO_4$, filtered and concentrated *in vacuo*.. Compound **(b)** was purified by silica gel flash-column chromatography using DCM as the eluent. The product was obtained as a dark pink solid (113.7 mg, 0.421 mmol, 68 %). ^{1}H NMR (400 MHz, $CDCl_3$) δ 6.09 (s, 2H), 4.96 (s, 2H), 2.52 (d, 6H), 2.46 (d, 6H), 1.56 (s, 1H), 0.18 (s, 6H). Data is consistent with that given in the literature.[8-10]

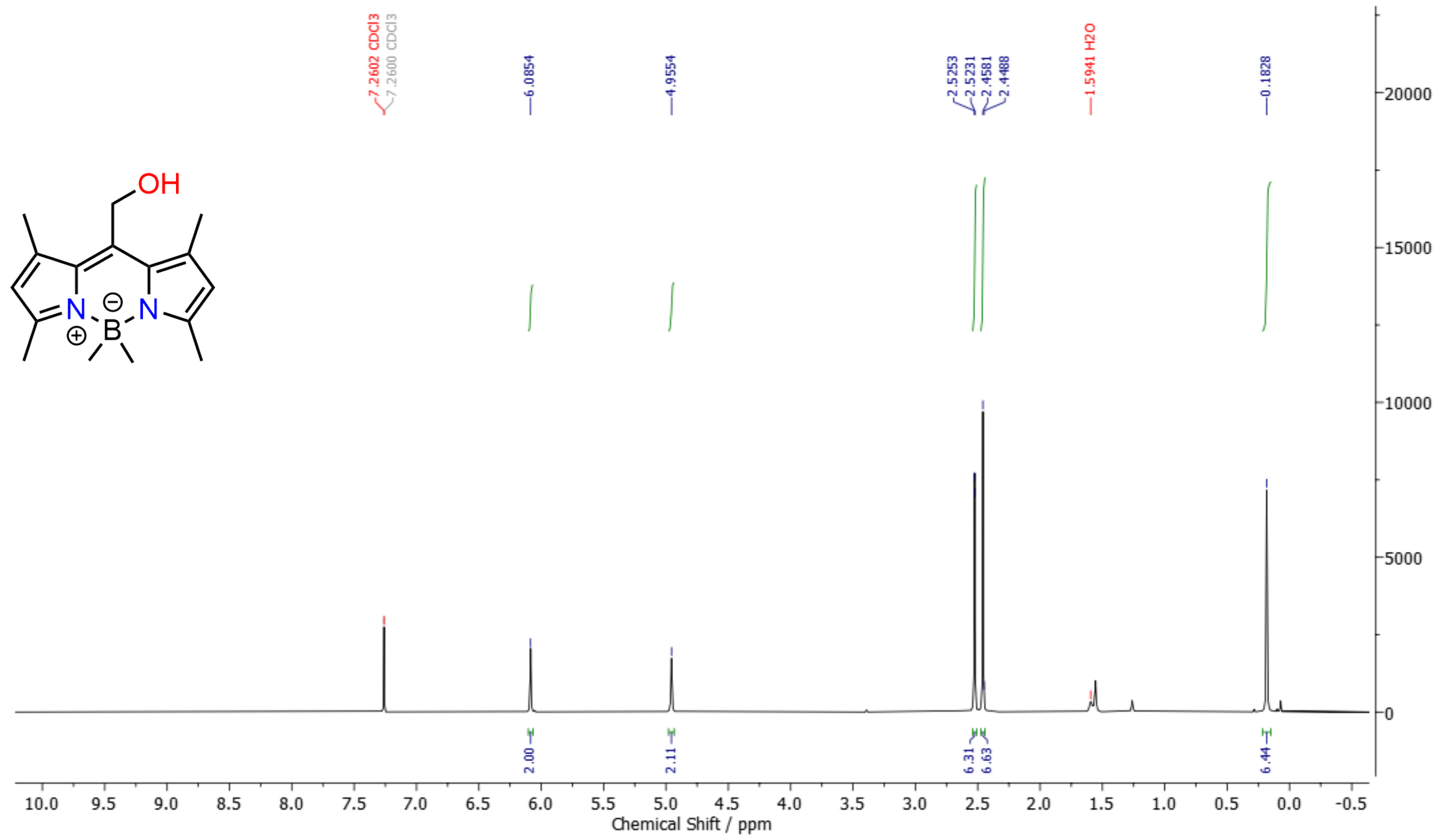

Figure S23: ^{1}H NMR Spectrum of **(b)** ($CDCl_3$, 298 K, 400 MHz)

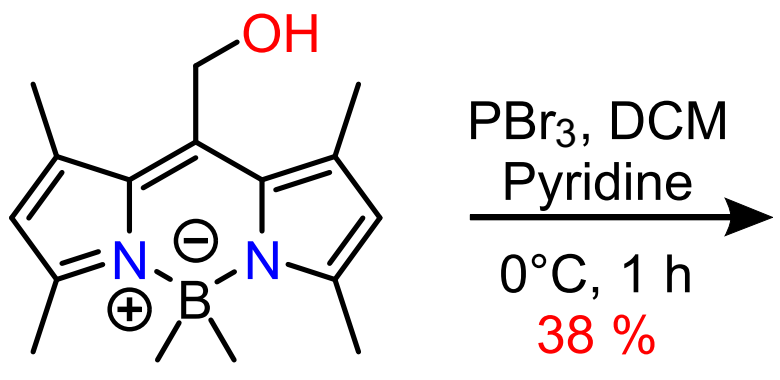

b **II**

Scheme S11: Synthesis of 8-Bromomethyl-1,3,5,7-tetramethyl pyrromethene methylborate

8-Bromomethyl-1,3,5,7-tetramethyl pyrromethene methylborate: Prepared by literature procedure.[9] Pyridine (66 µL, 0.8 mmol, 2 equiv.), followed by phosphorous tribromide (55 µL, 0.6 mmol, 1.5 equiv.) was added to a solution of 8-hydroxymethyl-1,3,5,7-tetramethyl pyrromethene methylborate **(b)** (110 mg, 0.41 mmol, 1 equiv.) in dry DCM (5.5 mL) at 0 °C under a nitrogen atmosphere. The reaction mixture was stirred in the dark for 1 h, after which the reaction was complete by TLC. Ice water (20 mL) was added to the reaction mixture and the organic layer was extracted with DCM (10 mL). The organic layer was washed with saturated Na_2CO_3 (20 mL), saturated NH_4Cl (20 mL), and brine (20 mL), and then dried over $MgSO_4$, filtered and concentrated *in vacuo.*. Compound **II** was purified by silica gel flash-column chromatography using hexane:DCM 9:1 as the eluent. The product was obtained as a reddish solid (50.9 mg, 0.15 mmol, 38 %). ^{1}H NMR (400 MHz, $CDCl_3$) δ 6.09 (s, $2H_{2,6}$), 4.77 (s, $2H_9$), 2.58 (d, *J* = 0.9 Hz, $6H_{11,12}$), 2.46 (d, $6H_{13,14}$), 0.21 (s, $3H_{15/16}$), 0.15 (s, $3H_{16/15}$). Data consistent with that given in the literature.[9]

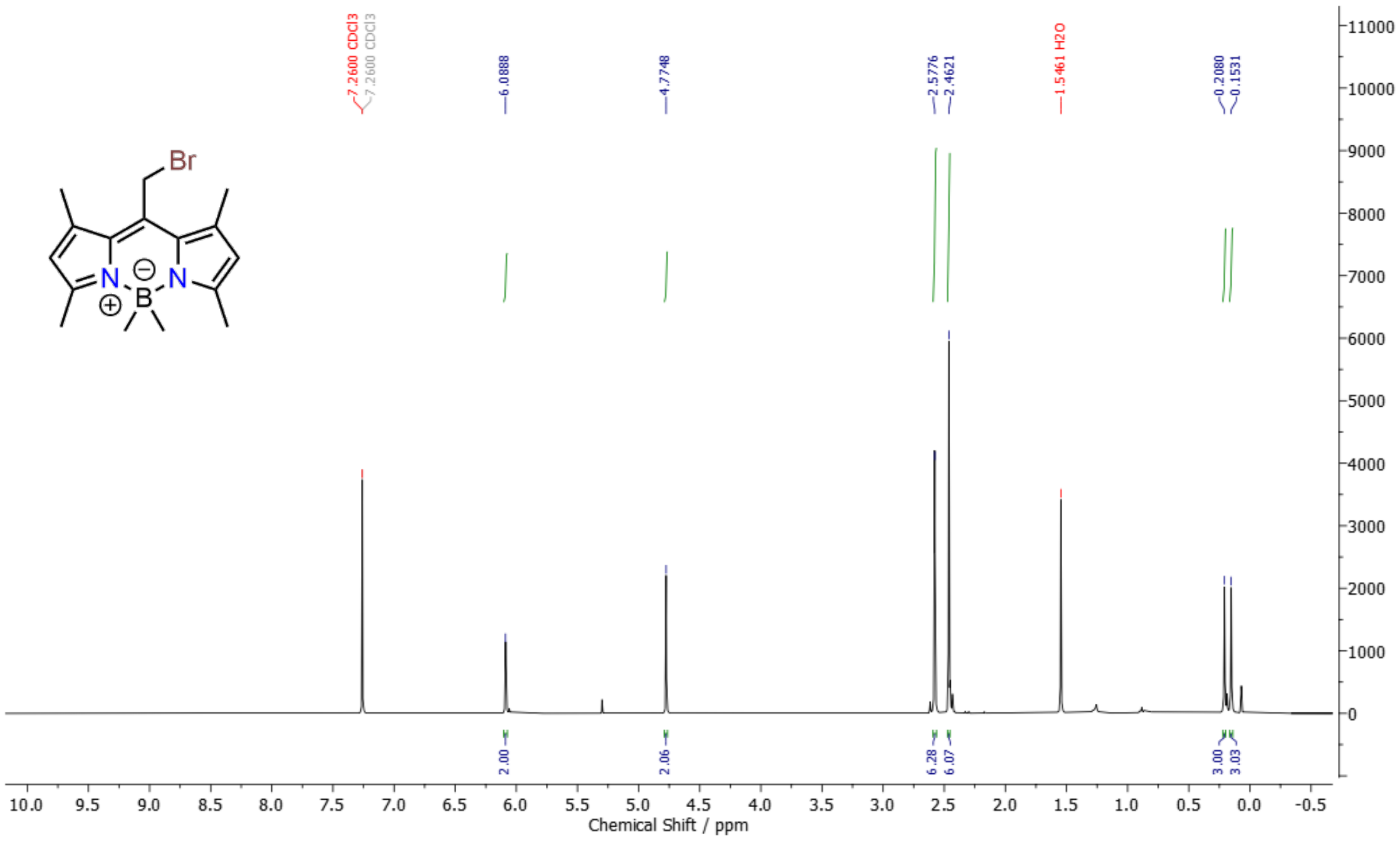

Figure S24: ^{1}H NMR Spectrum of **II** ($CDCl_3$, 298 K, 400 MHz)

3 UV-Vis Titration Experiments

UV-Vis spectra were recorded on a V-770 UV-Visible/NIR Spectrophotometer equipped with Peltier temperature controller and stirrer using quartz cuvettes of 1 cm path length. Experiments were conducted at 25°C. UV-visible titration anion binding experiments were performed to investigate the binding affinities of the indolocarbazole compounds to chloride. Compounds **1**, **2**, and **3** were dissolved in acetonitrile at a concentration of 10 µM. The anionic guest (as the tetrabutylammonium (TBA) salt), was dissolved in the host stock solution at a concentration of 10 mM, and aliquots added to 1.0 mL of the host solution in a quartz glass cuvette. The UV-visible spectra were recorded upon successive additions of the TBA salt solution. The absorbance intensities of the peak maxima were fitted to a 1:1 stoichiometric host-guest binding isotherm using the Musketeer software.[11, 12]

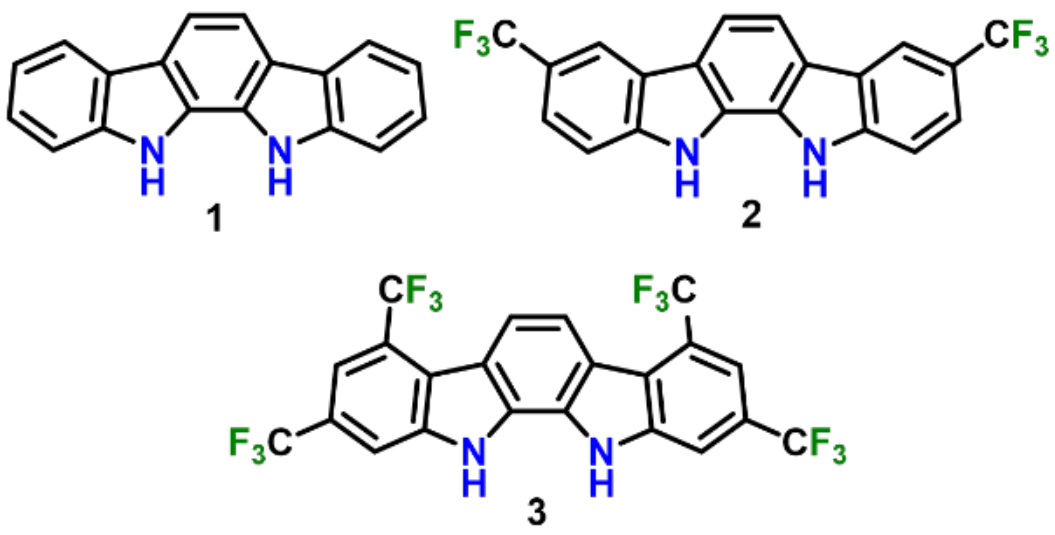

Compound	ClogP	K (M^{-1}) /Cl$^-$ [a]	EC$_{50}$ /mol % [b]	n [c]
**1**	4.11	1900 ± 80	- [d]	-
**2**	6.12	6705 ± 135	1.4 ± 0.1	1.0 ± 0.08
**3**	8.06	8710 ± 440	> 15 [d]	1.1 ± 0.26

Figure S25: **LEFT** Indolocarbazole derivatives **1**, **2**, and **3**. **RIGHT** Clog *P* values were calculated using Swiss ADME.[13] ***a*** Binding constants were obtained by UV-Vis spectroscopy (10 µM, in acetonitrile), using Musketeer fitted to a 1 : 1 binding isotherm.[11, 12] ***b*** Effective concentration to reach 50% of maximal activity across POPC-LUVs (200 nm, lipid concentration 31mM) encapsulating 1 mM HPTS, 100 mM NaCl, buffered at pH 7.0 using 10 mM HEPES. ***c*** Hill coefficient. ***d*** EC$_{50}$ could not be reliably determined due to the lack of transport activity of **1**, and insolubility of **3**.

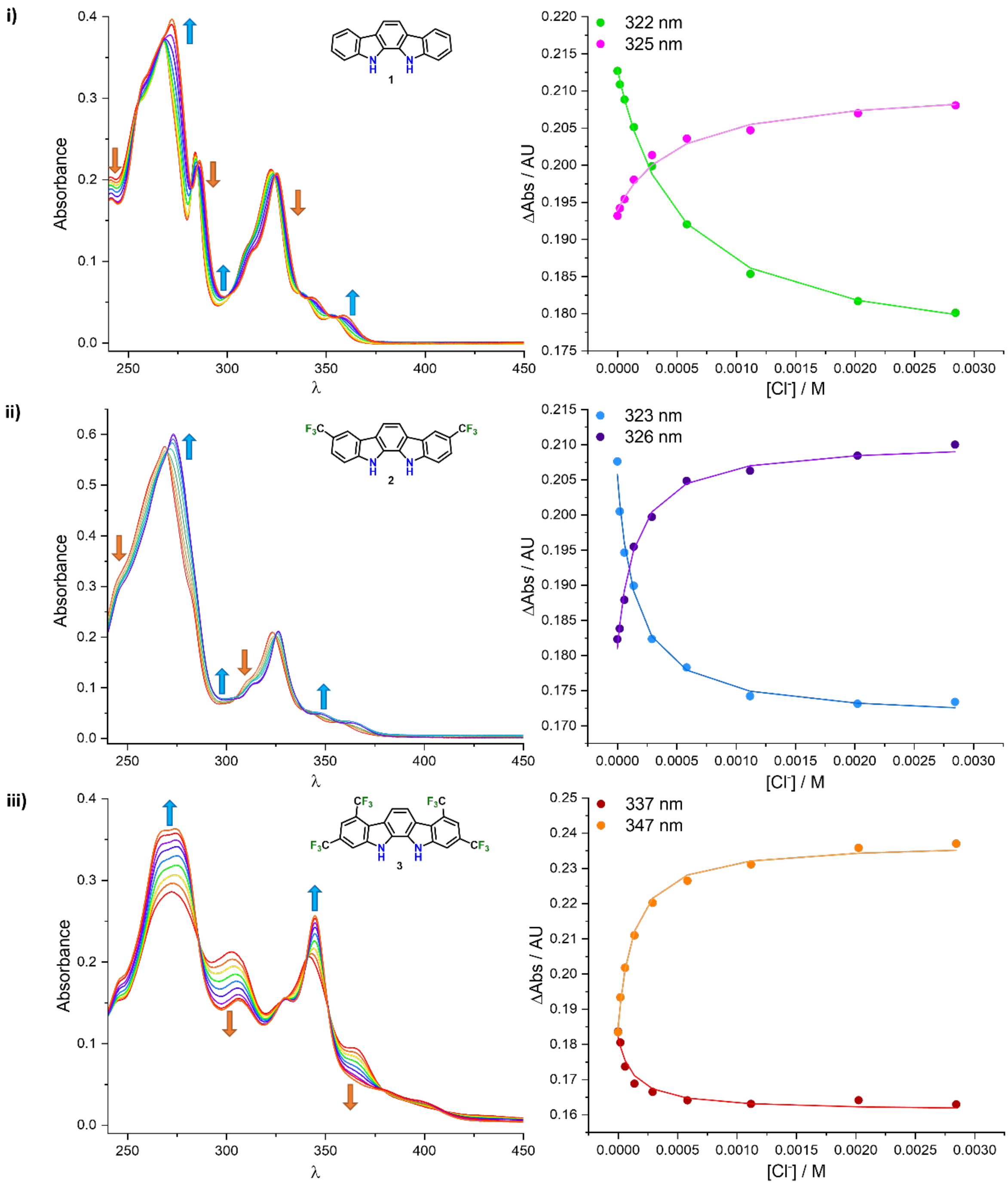

Figure S26: **LEFT** UV-Visible Spectra of 10 µM solution of host in acetonitrile upon successive addition of 10 mM TBACl salt of **(i) 1**, **(ii) 2**, and **(iii) 3**. **RIGHT** Chloride binding isotherm for **(i) 1**, **(ii) 2**, and **(iii)**. Experimental data shown by symbols (●), fitted 1:1 binding isotherm shown by solid lines (─).

4 ^{1}H NMR Titration Experiments

Chloride binding of the double-photocaged **4**, and mono-photocaged **5** compounds were assessed by ^{1}H NMR titrations using a Bruker AVIII 500 spectrometer at 500 MHz and 298 K. The initial sample volumes were 0.5 mL, at a concentration of 1 mM of host dissolved in 2.0% D_2O-acetone-d_6 (v/v). Known volumes of the anion guest, as the tetrabutylammonium (TBA) salt in the same solvent were added to the host and the spectra were recorded after 0, 0.1, 0.2, 0.3, 0.4, 0.5, 1.0, 2.0, 3.0, 4.0, 6.0, 8.0, and 10.0 equivalents. The chemical shift perturbations of the host spectra were monitored as a function of guest concentration. The spectra were referenced to the residual solvent peak. The data was analysed using a global fit procedure using the Bindfit program,[12, 14] using non-linear least squares analysis to obtain the best fit between observed and calculated chemical shifts for the 1:1 binding stoichiometry.

The binding affinity for **5** was obtained by fitting to a 1:1 stoichiometric host-guest binding model using Bindfit,[12, 14] with a binding association constant, $K_{1:1}$, of 30 M^{-1}. The association of guest and host was fast on the NMR timescale.

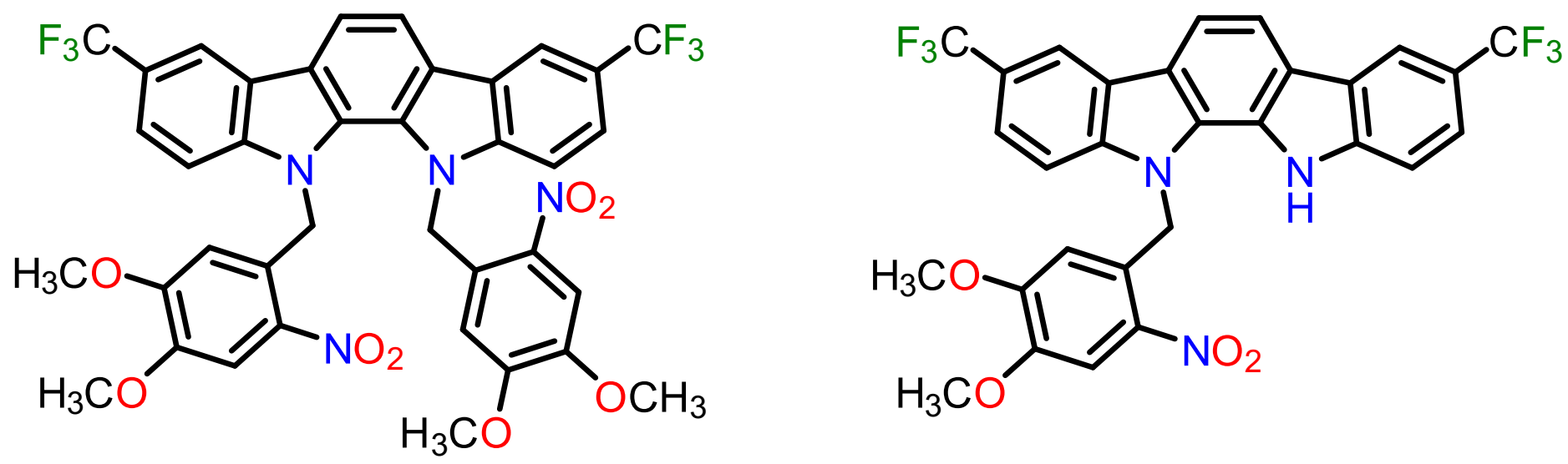

4 **5**

Figure S27: Double-caged transporter **4** and mono-caged transporter **5**

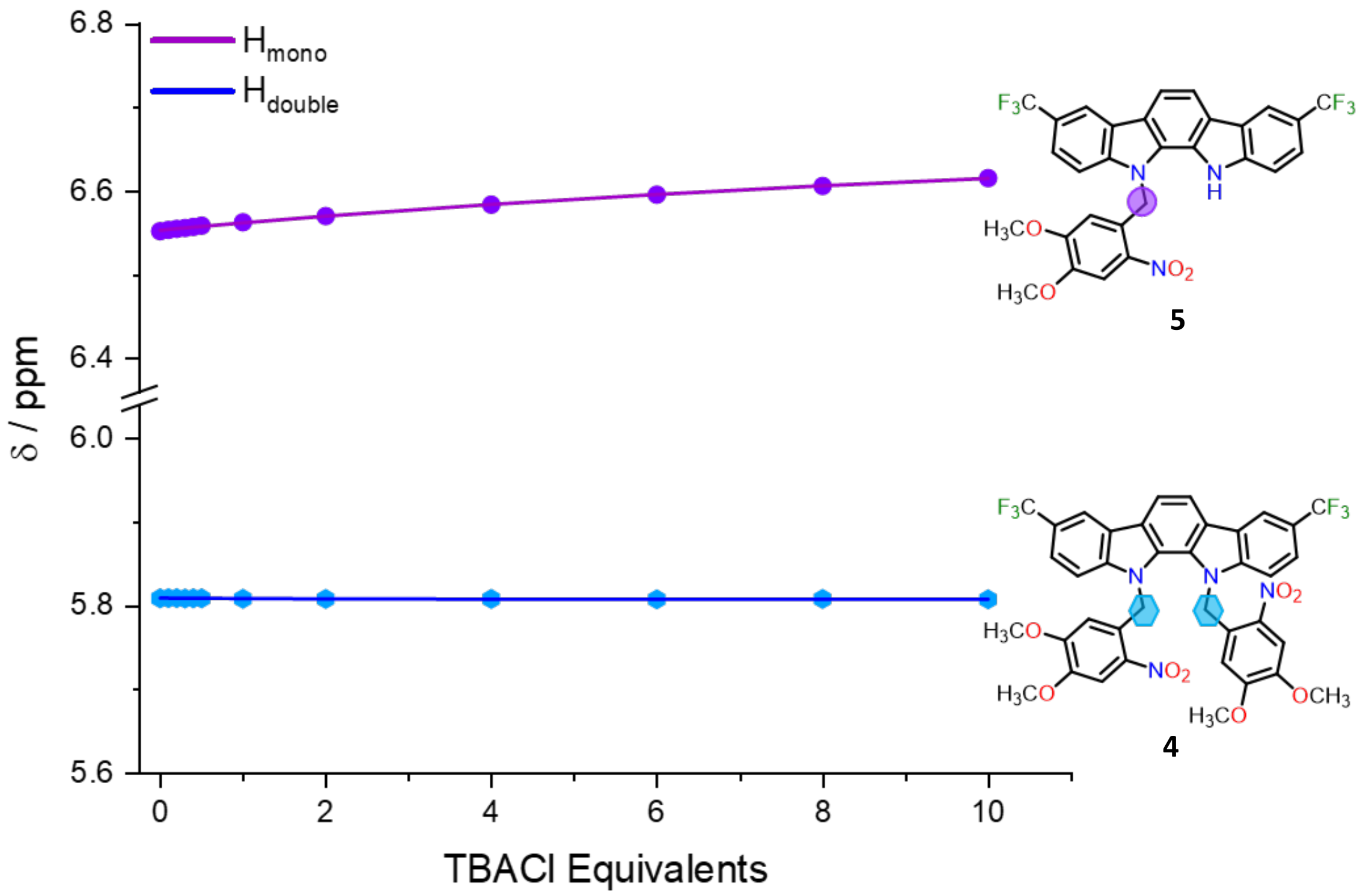

Figure S28: Comparison of chloride binding isotherms for **4** and **5** in 2.0 % D_2O-acetone-d_6. Experimental data shown by symbols, fitted 1:1 binding isotherm shown by solid lines.

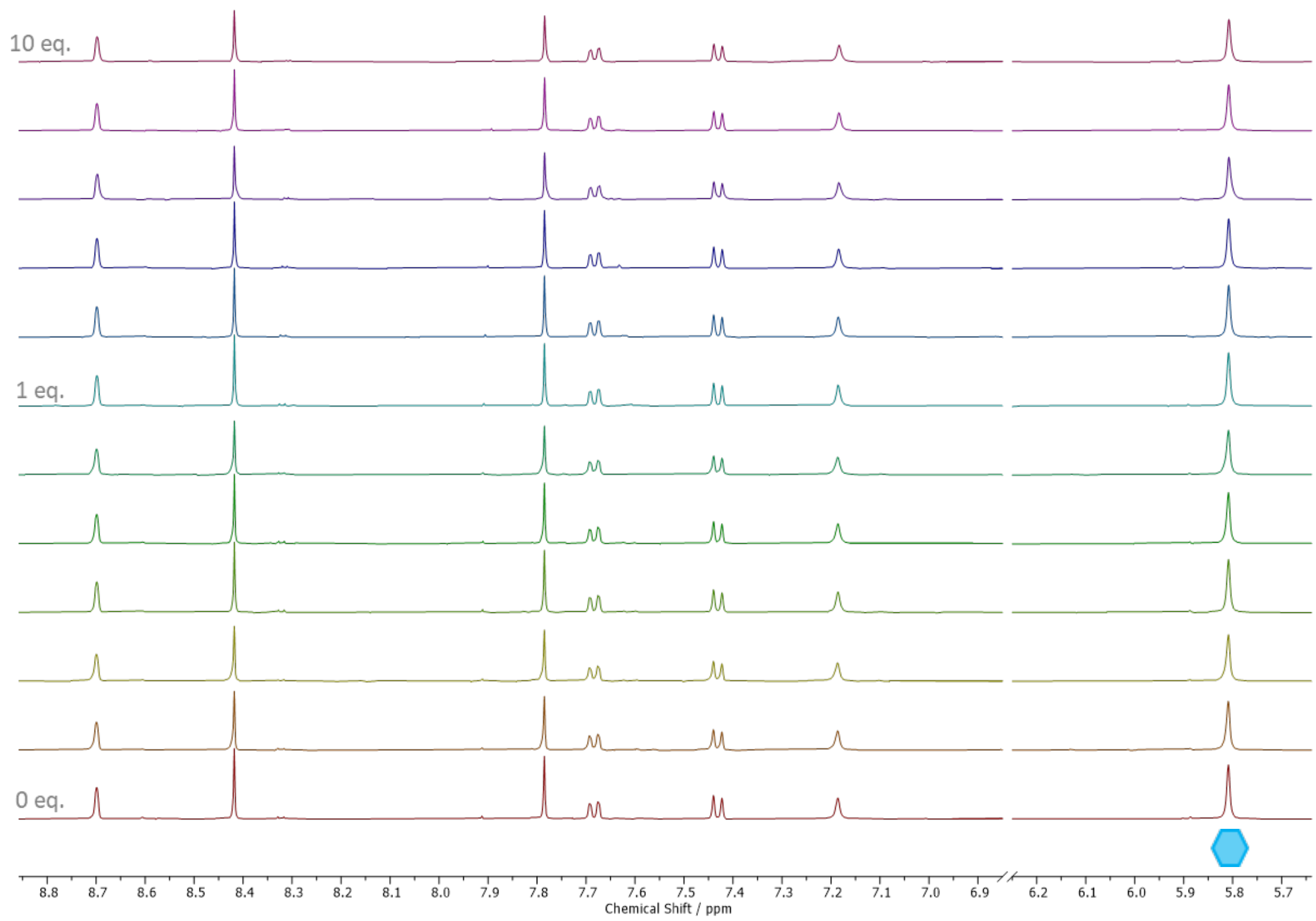

Figure S29: Stacked partial ^{1}H NMR Spectra of **4** with increasing equivalents of TBACl

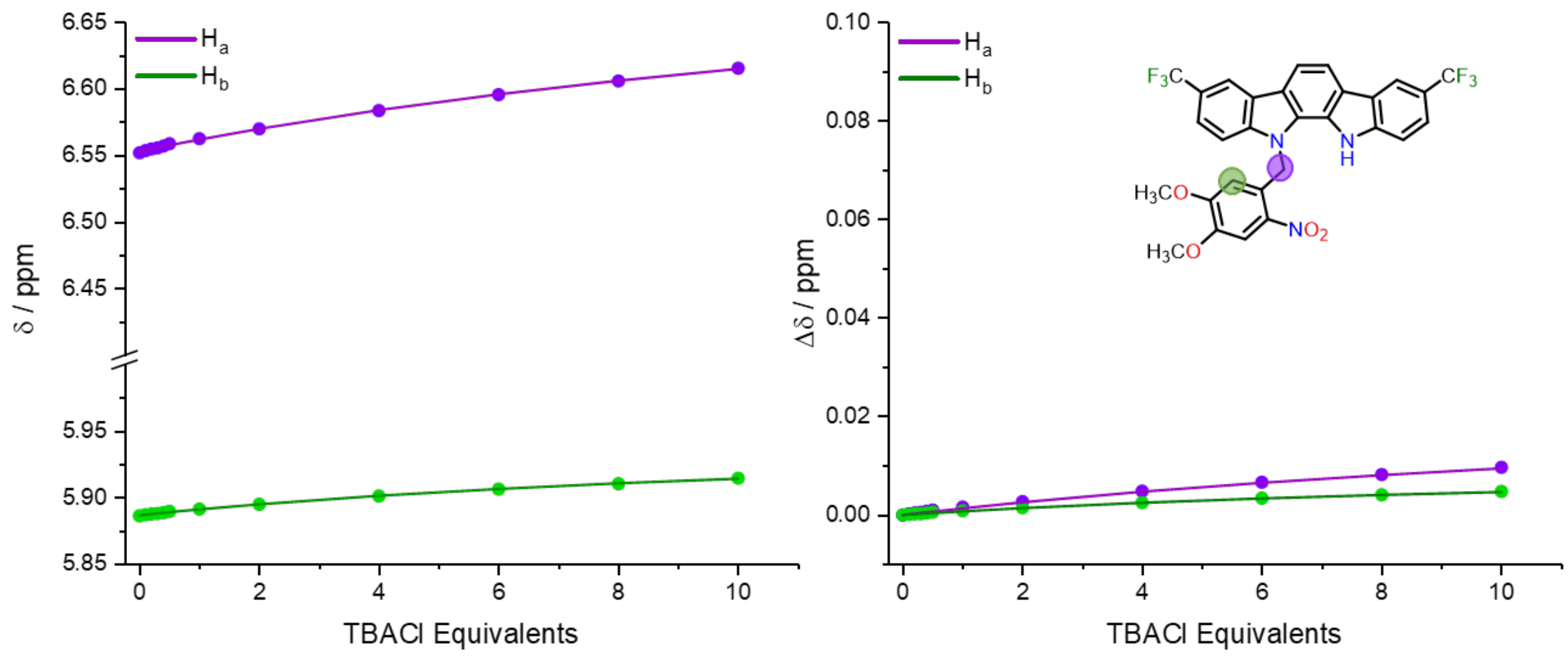

Figure S30: LEFT Chloride binding isotherms for **5** in 2.0% D_2O-acetone-d_6. Experimental data shown by symbols, fitted 1:1 binding isotherm shown by solid lines. **RIGHT** Change in chemical shift .

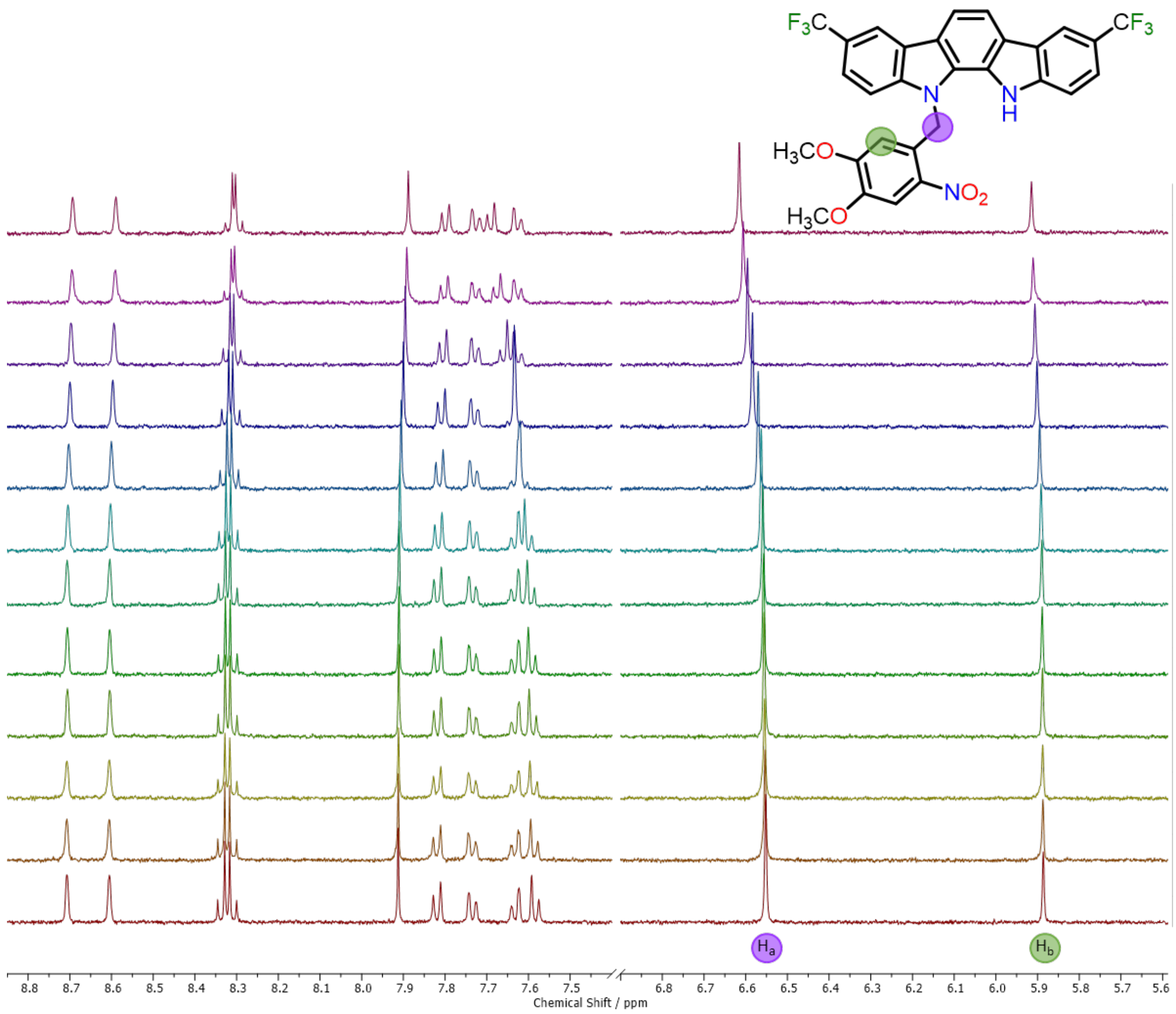

Figure S31: Stacked partial ^{1}H NMR Spectra of **5** with increasing equivalents of TBACl

5 Photocleavage Experiments

5.1 Irradiation Set-up

Photo-irradiation of samples was carried out using Thorlabs high-power mounted LEDs (M530L4, 530 nm, 370 mW; M405L4, 405 nm, 1 W; M365L3, 365 nm, 880 mW) using an in-house custom-built set-up with optical components supplied by Thorlabs (Figure S32).[15] For irradiating small vials of samples, NMR tubes and cuvettes, a Thorlabs cuvette holder (CVH100/M) equipped with the mounted LEDs was used. Samples were irradiated for various times to reach the photocleaved state, as confirmed by ^{1}H NMR or observed by UV-vis experiments. The same set-up was used for *in situ* anion transport experiments in LUVs, whereby the cuvette containing HPTS vesicles and photo-caged transporter was placed in the cuvette holder and covered for specific irradiation times.

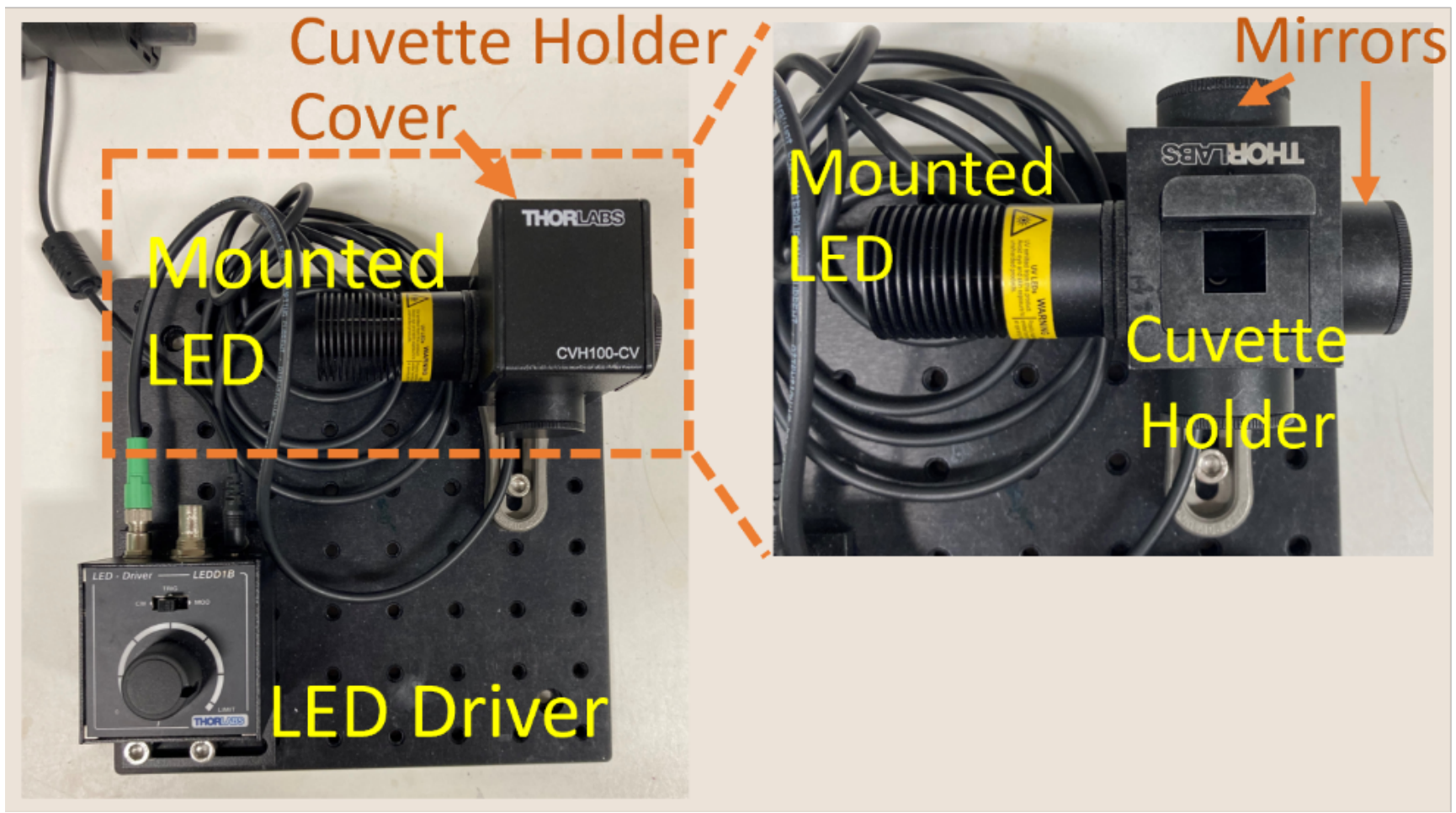

Figure S32: In-house custom-built set-up for irradiating samples for de-caging analysis by ^{1}H NMR, UV-Vis, and for LUV transport experiments.

5.2 UV-Vis Analysis

Photocleavage of **4** and **5** was observed by UV-vis spectroscopy by irradiating 20 µM samples in DMSO with Thorlabs mounted LEDs at 405 nm (using M405L4) in a Thorlabs cuvette holder (CVH100/M). The LEDs were supplied with 1 A current using Thorlabs T-cub LED driver (LEDD1B). The deprotection was monitored by UV-vis spectra at known time intervals.

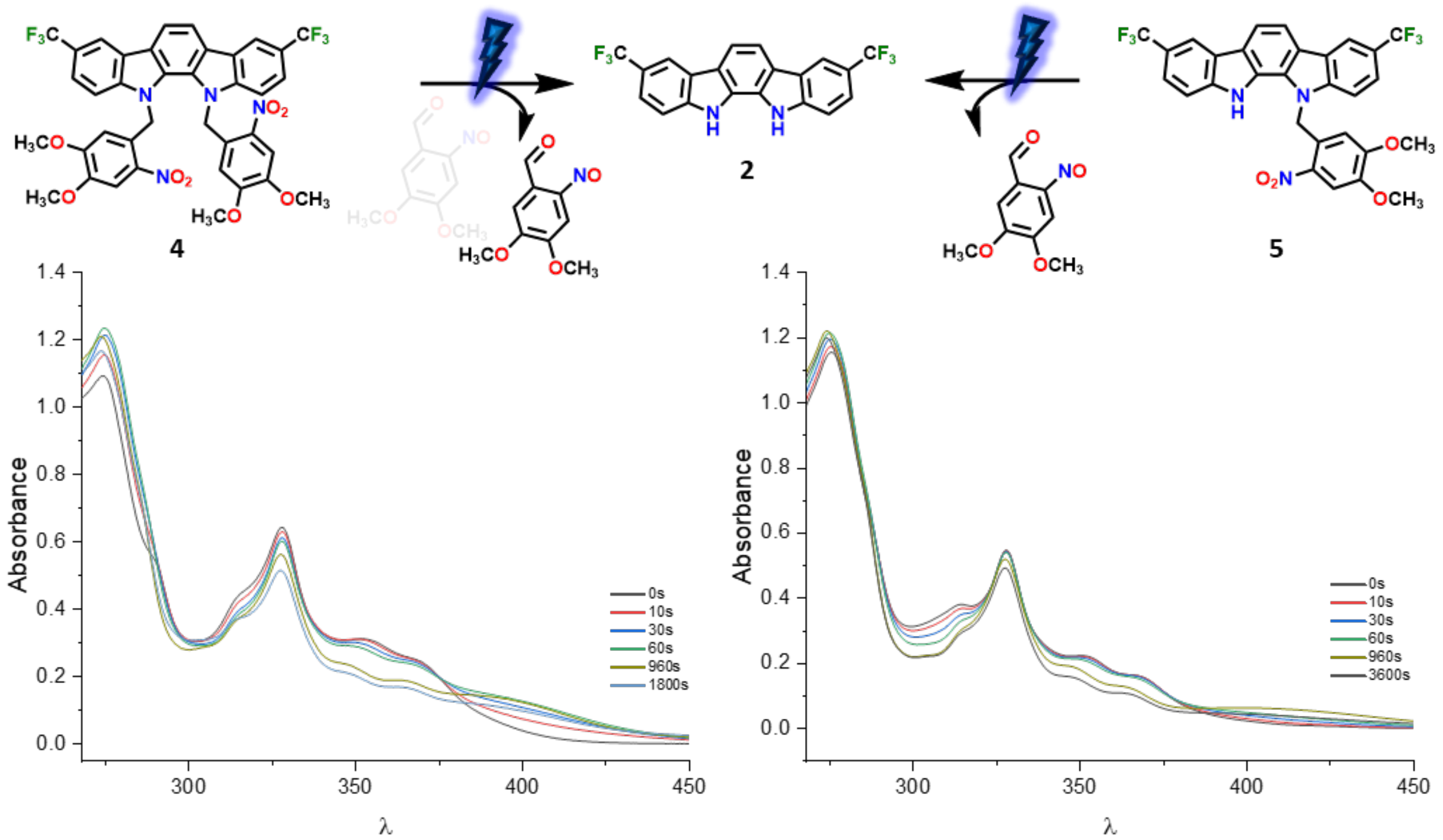

Figure S33: Stacked spectra of **4** (left) and **5** (right) after increasing irradiation times (given) with a 405 nm LED (1 W)

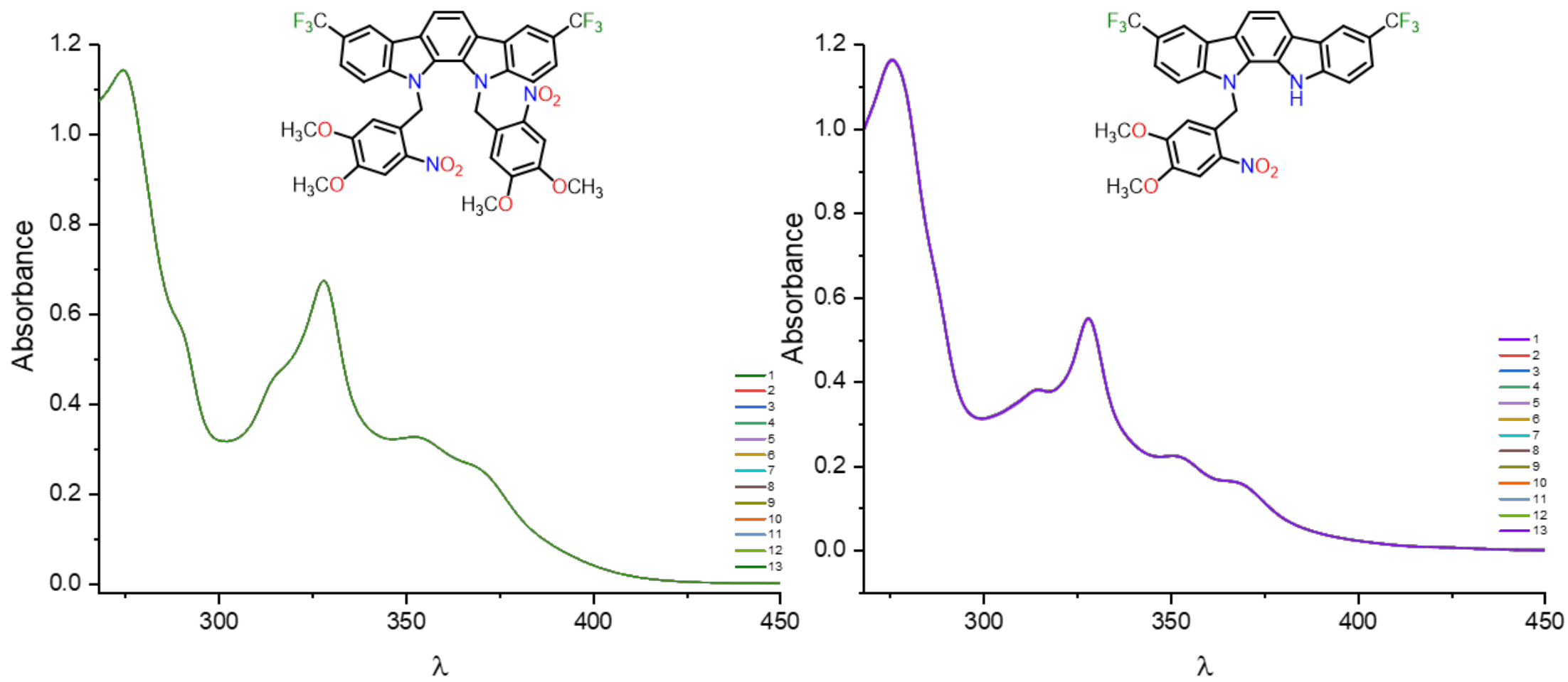

Figure S34: Stacked spectra of **4** (left) and **5** (right) after 13 successive UV-Vis spectra recordings. This control experiment demonstrates that no photocleavage is induced by irradiation by the spectrometer

5.3 ^{1}H NMR Analysis

Photocleavage of **4** and **5** was conducted in the solution phase by irradiating 1 mM samples with Thorlabs mounted LED at 405 nm (using M405L4) in a Thorlabs cuvette holder (CVH100/MM). The LED was supplied with 1 A current using Thorlabs T-cub LED driver (LEDD1B). The deprotection was monitored using ^{1}H NMR experiments at known time intervals.

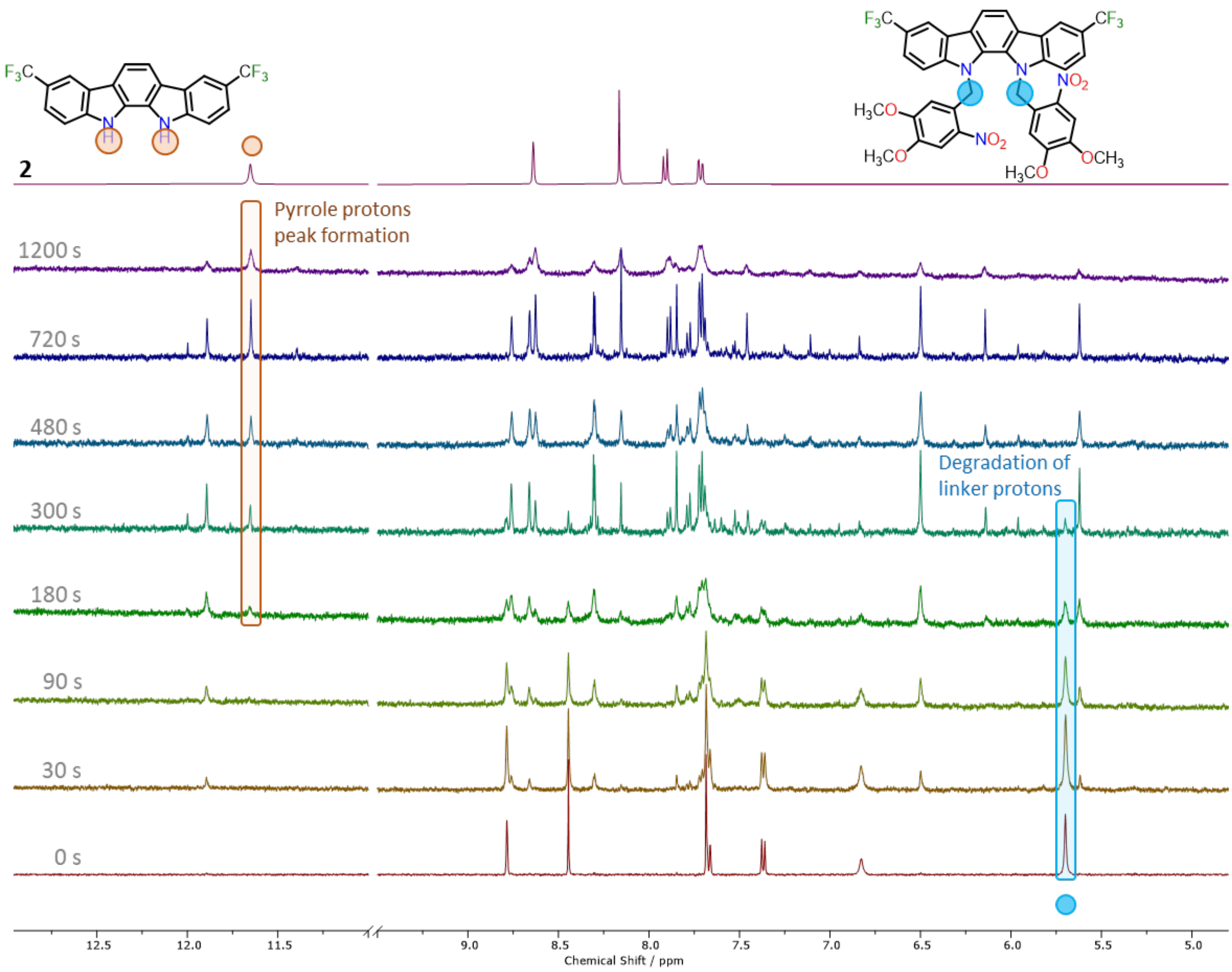

Figure S35: Stacked partial ^{1}H NMR Spectra of **4** (1 mM sample in DMSO-d_6, 298 K) with increasing irradiation times (0-1200 seconds) with a 405 nm LED (1 W); the spectrum of the free transporter **2** is included for reference.

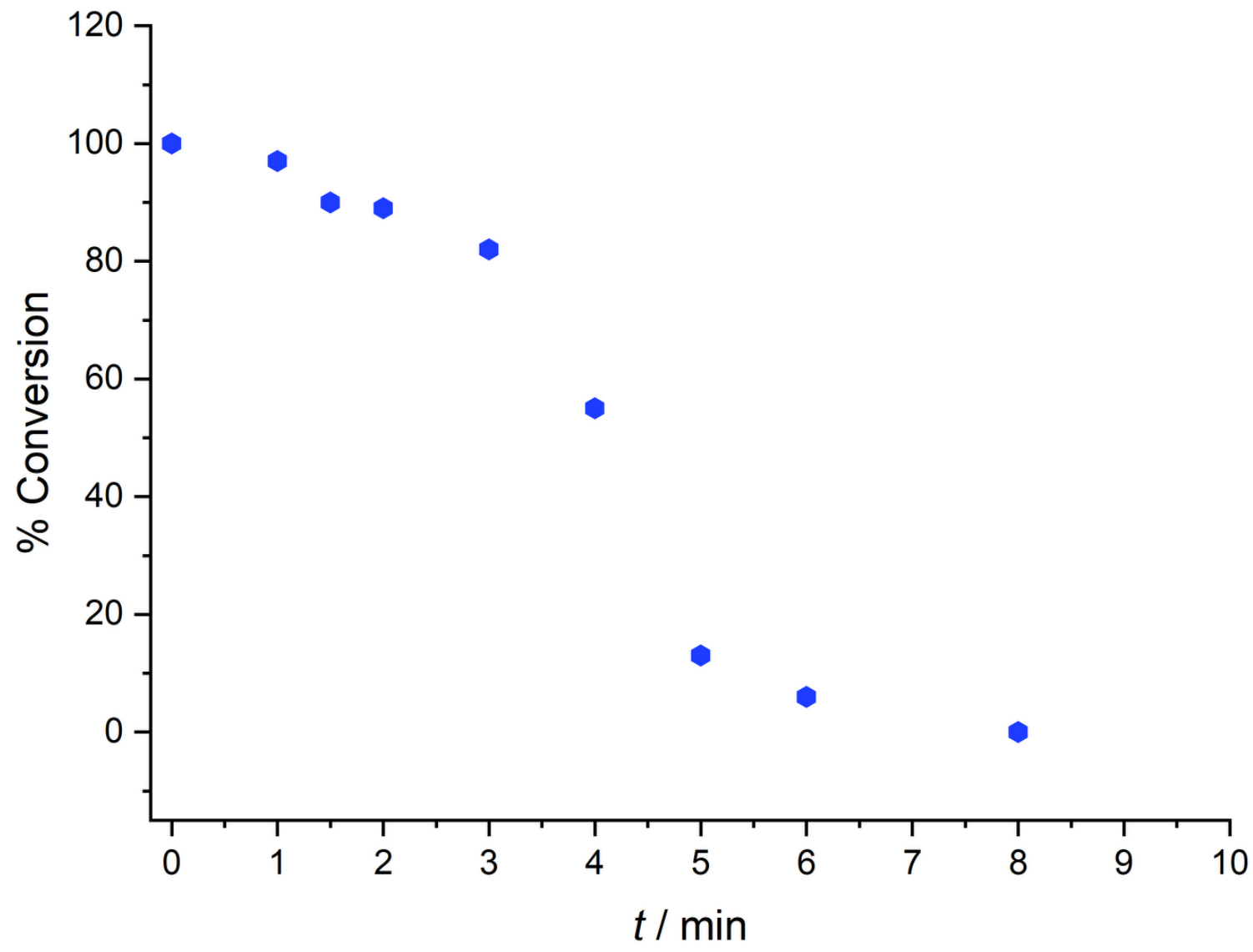

Figure S36: The % conversion of double-photocaged transporter **4** after irradiation with a 405 nm LED (1 W). Full decaging was achieved after 8 minutes in these conditions.

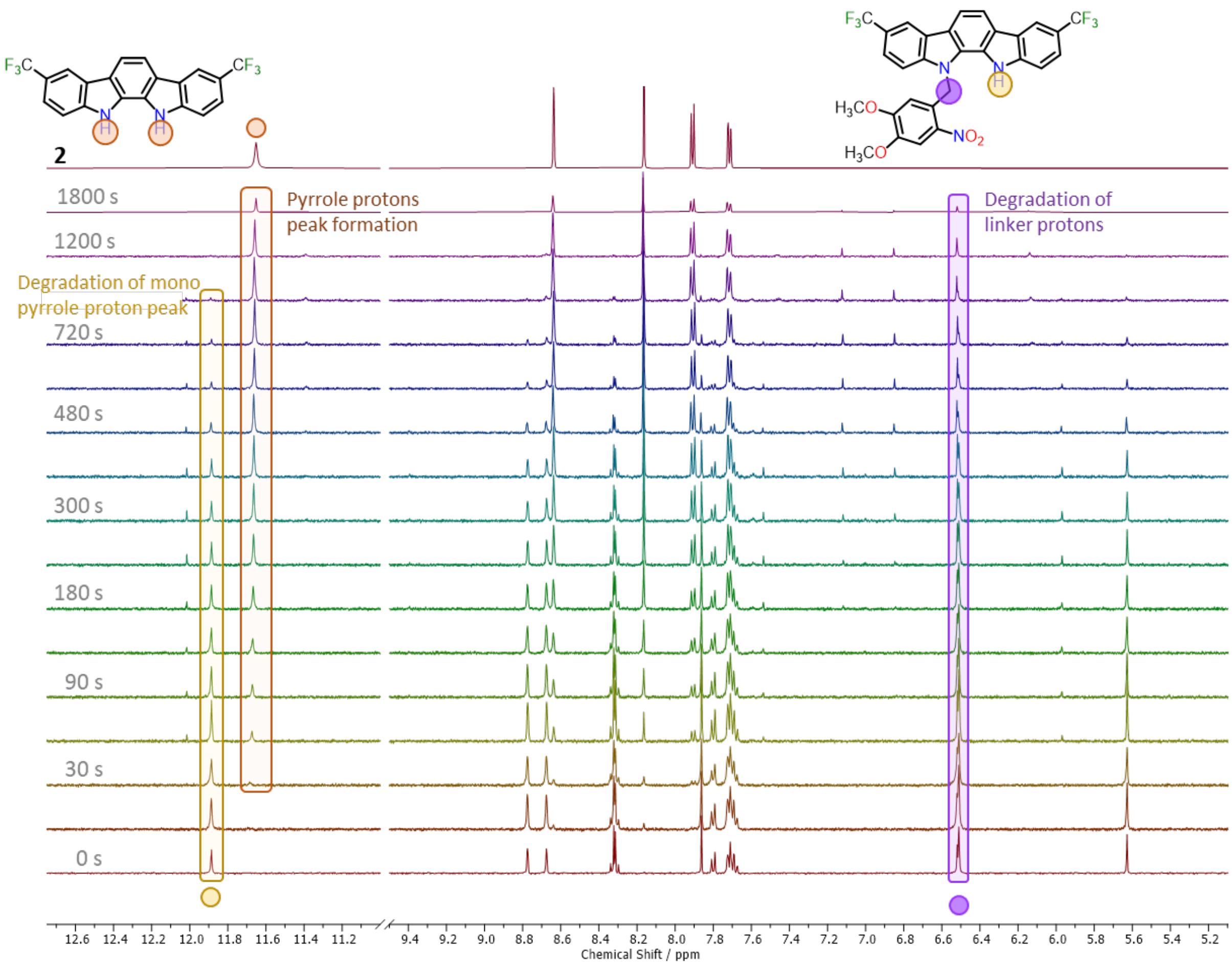

Figure S37: Stacked partial ^{1}H NMR Spectra of **5** (1 mM sample in DMSO-d_6, 298 K) with increasing irradiation times (0-1800 seconds) with a 405 nm LED (1W); the spectrum of the free transporter **2** is included for reference.

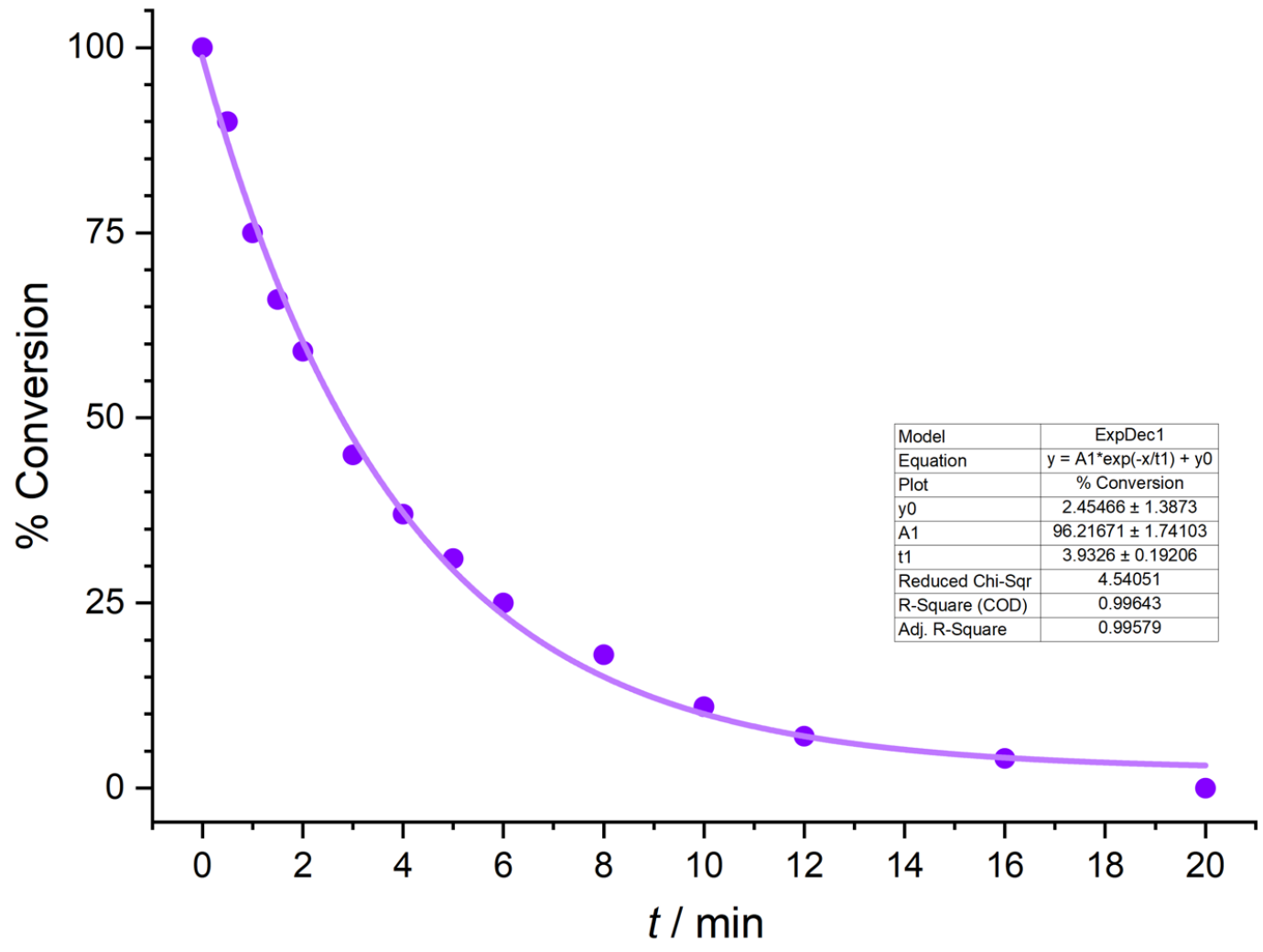

Figure S38: The % conversion of mono-photocaged transporter **5** after irradiation with a 405 nm LED (1 W). The half-life of decaging was determined as $t_{½}$ = 157 s.

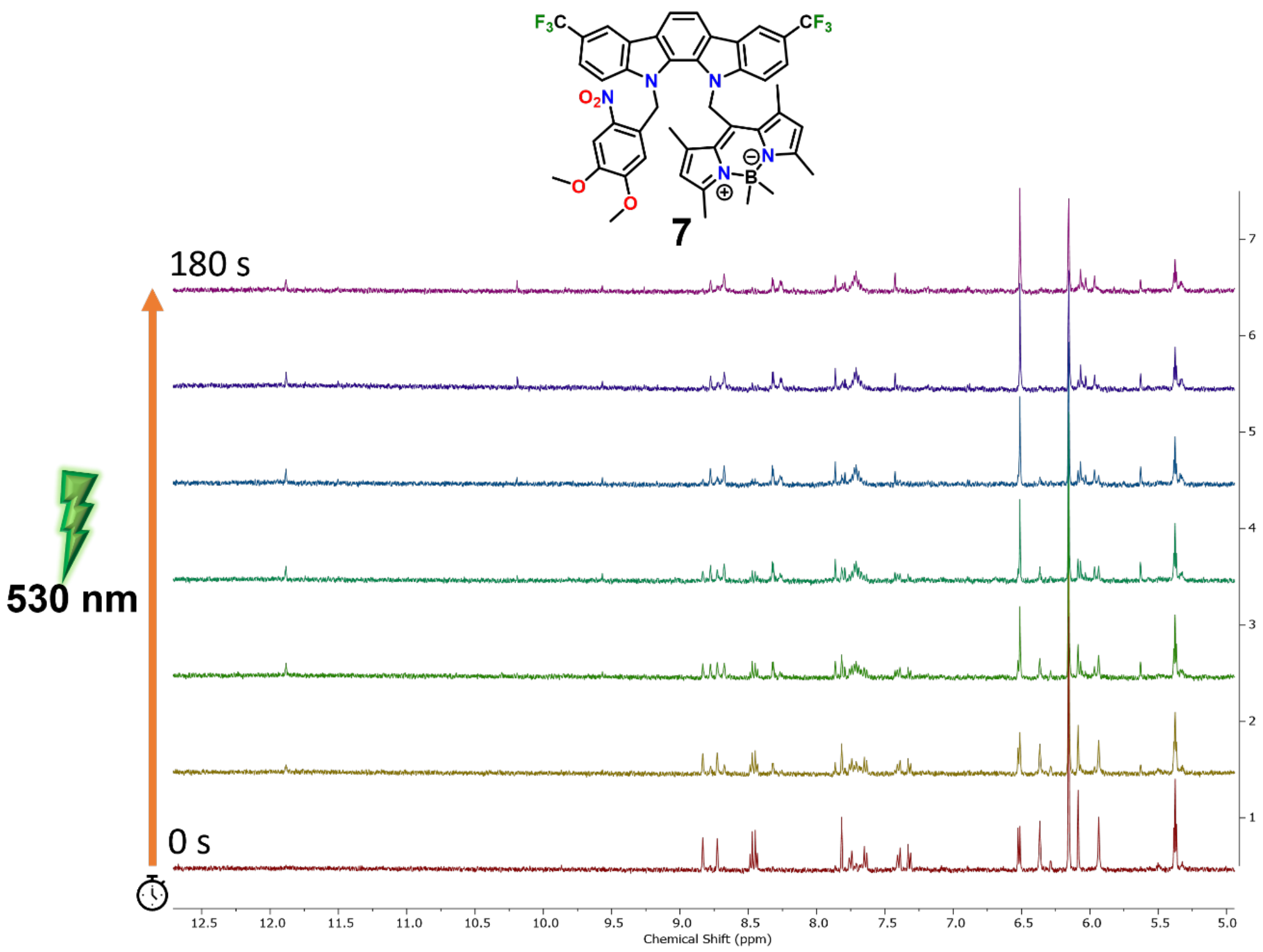

Figure S39: Stacked partial ^{1}H NMR Spectra of **7** (1 mM sample in DMSO-d_6, 298 K) with increasing irradiation times (0-180 seconds) with a 530 nm LED (370 mW)

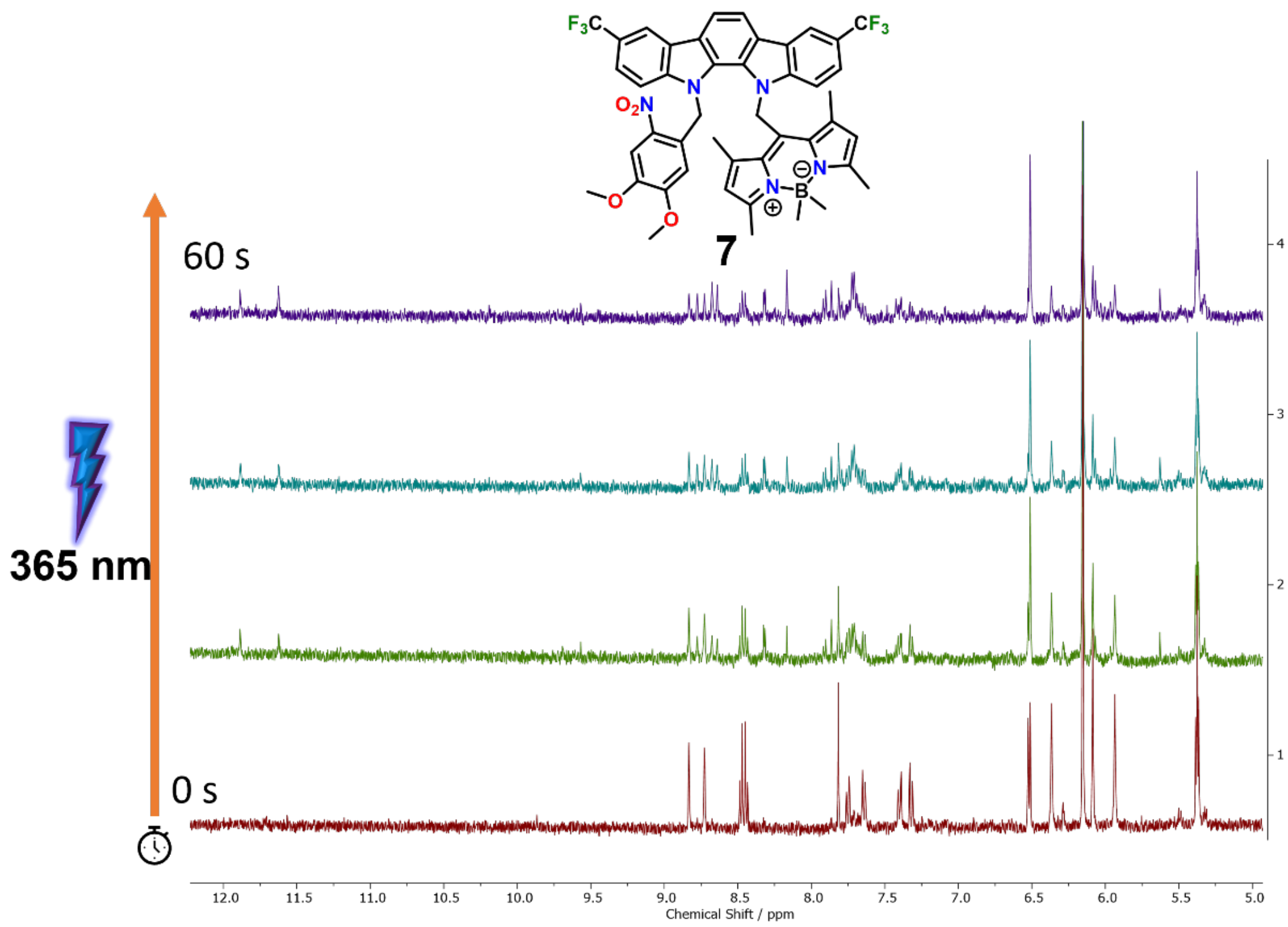

Figure S40: Stacked partial ^{1}H NMR Spectra of **7** (1 mM sample in DMSO-d_6, 298 K) with increasing irradiation times (0-60 seconds) with a 365 nm LED (880 mW).

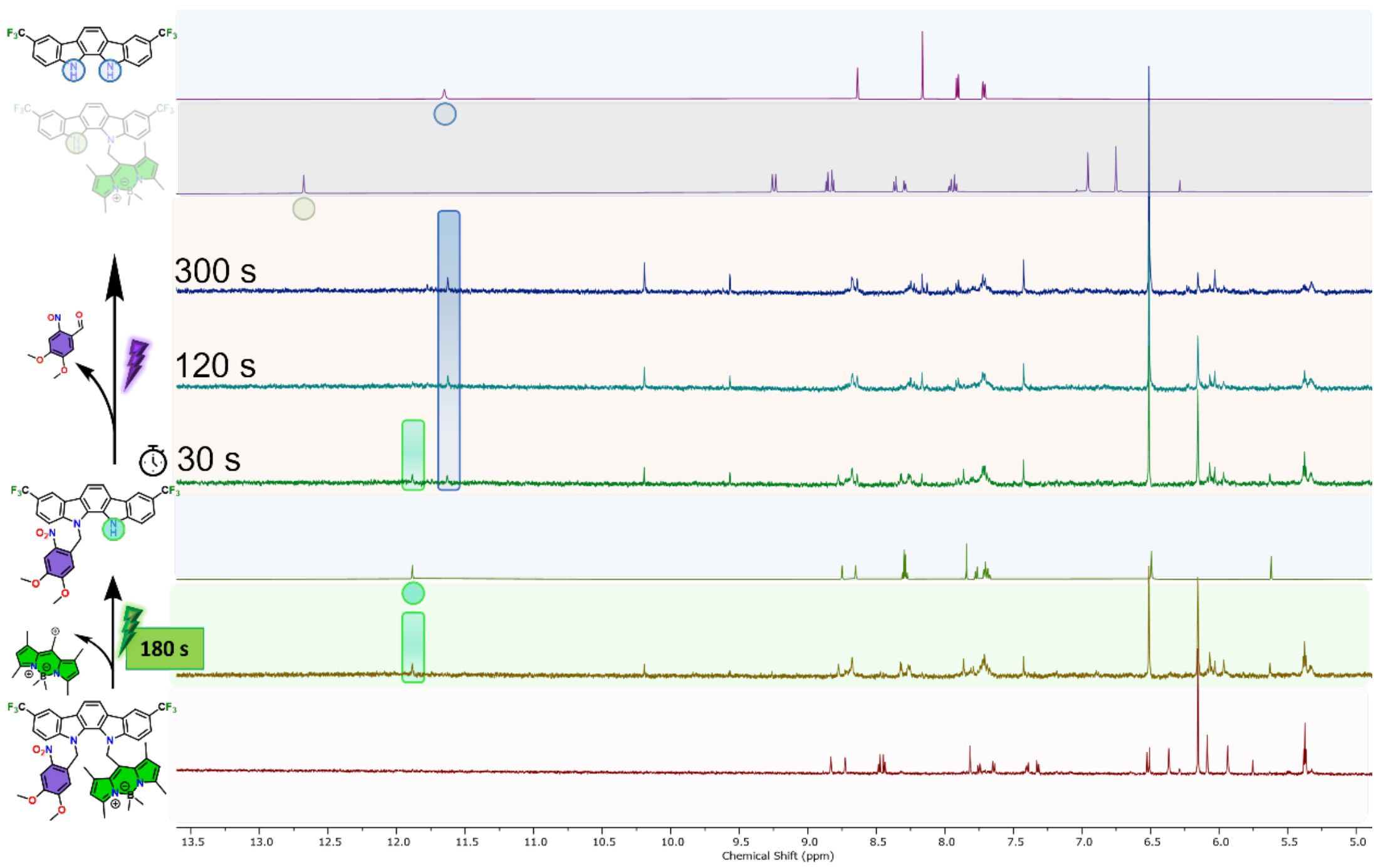

Figure S41: Stacked partial ^{1}H NMR Spectra of **7** (1 mM sample in DMSO-d_6, 298 K) showing sequential irradiation with a 530 nm LED (370 mW) for 180 s followed by irradiation with increasing times (30-300 seconds) with a 365 nm LED (880 mW).

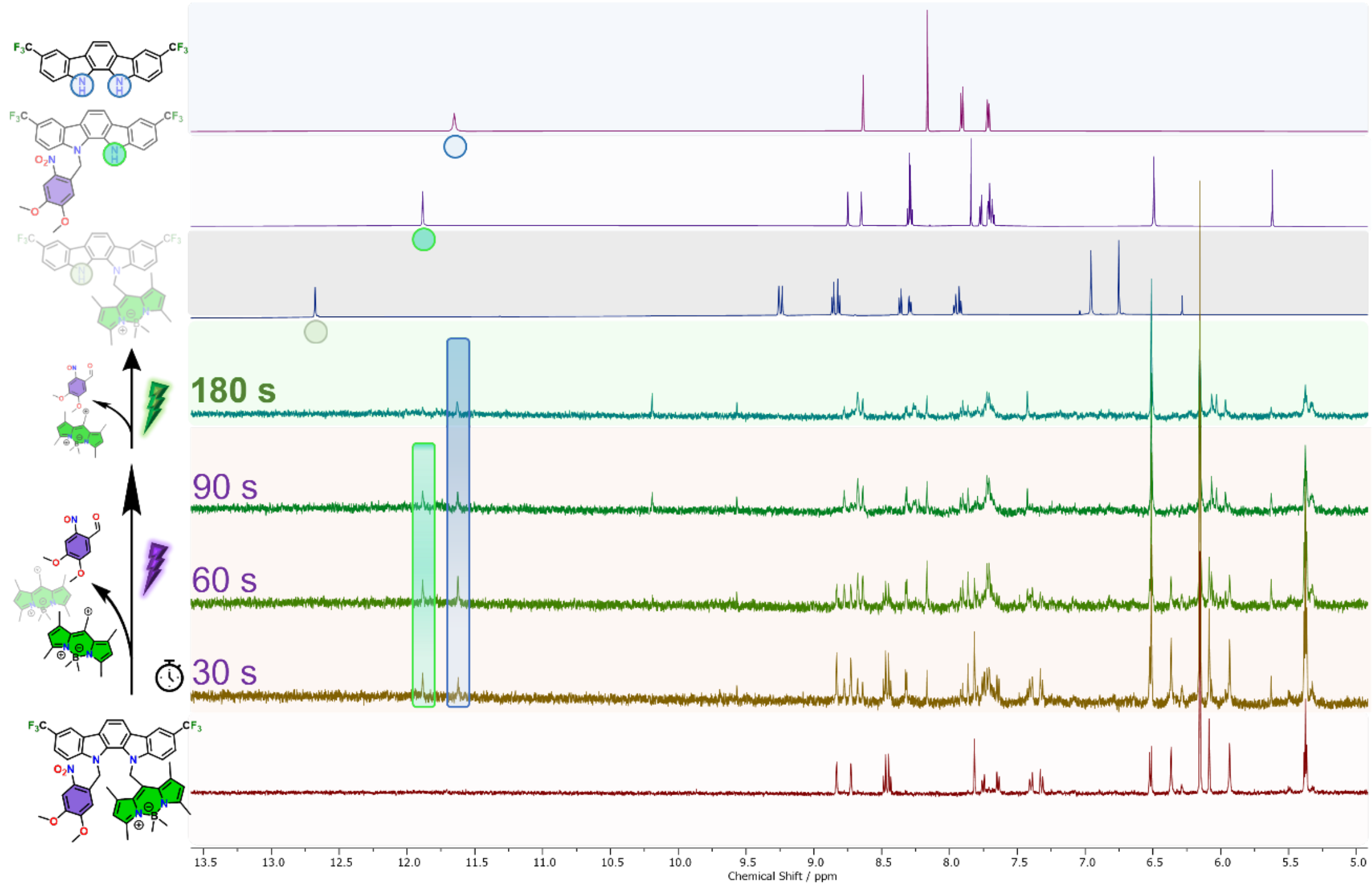

Figure S42: Stacked partial ^{1}H NMR Spectra of **7** (1 mM sample in DMSO-d_6, 298 K) showing sequential irradiation with a 365 nm LED (880 mW) with increasing times (0-90 seconds) followed by irradiation with a 530 nm LED (370 mW) for 180 s.

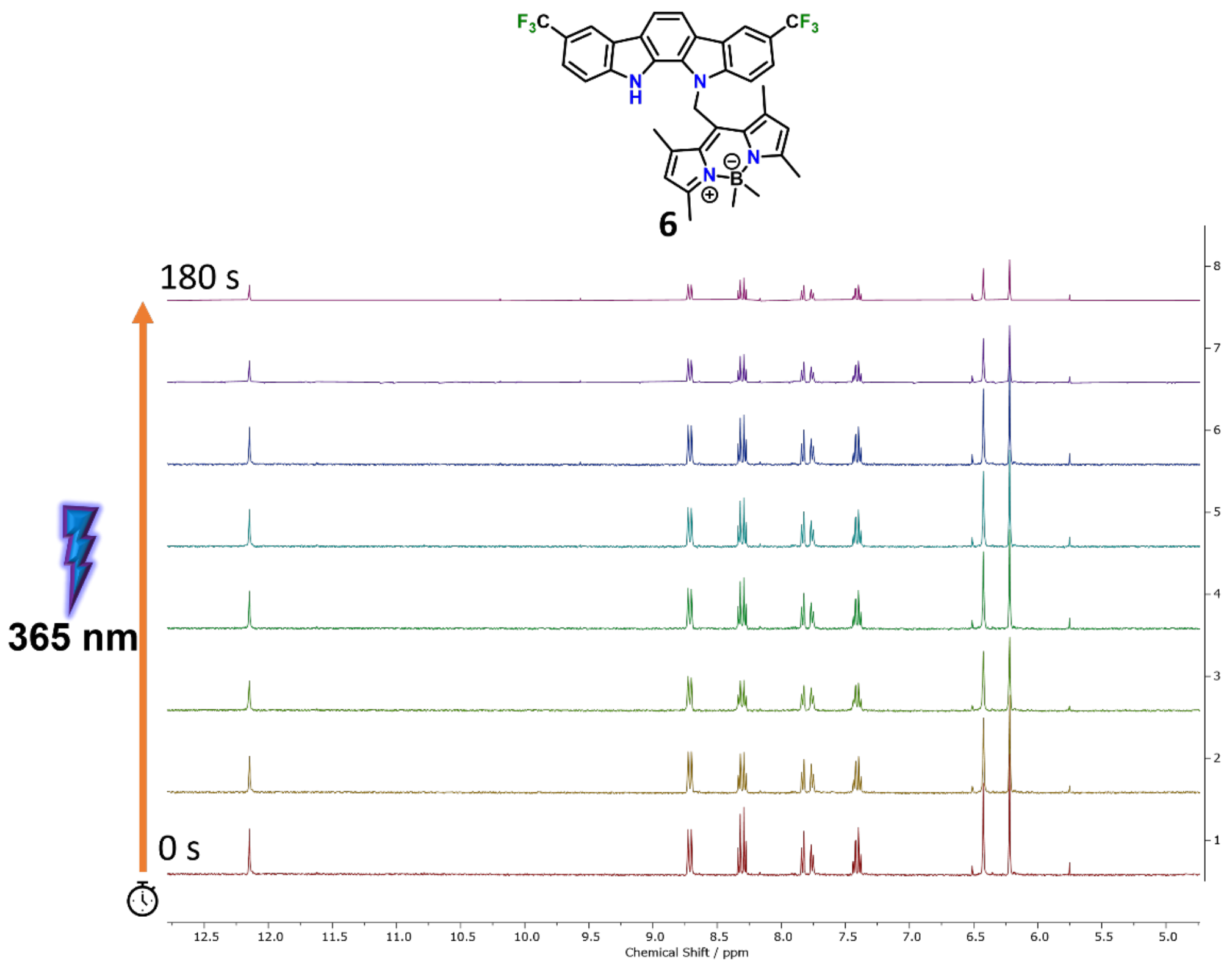

Figure S43: Stacked partial ^{1}H NMR Spectra of **6** (1 mM sample in DMSO-d_6, 298 K) with increasing irradiation times (0-180 seconds) with a 365 nm LED (880 mW) (Control Experiment).

6 Anion Transport Experiments

6.1 Vesicle Preparation

A thin film of lipid (1-palmitoyl-2-oleoyl-sn-3-phosphatidylcholine (POPC) or dipalmitoyl phosphatidylcholine (DPPC)) was formed by evaporating a chloroform solution of the lipid under reduced pressure on a rotary evaporator (40 °C) and then under high vacuum for 6 hours. Then the lipid film was hydrated by vortexing with 1 mL of the prepared internal buffer solution. The lipid suspension was then subjected to 5 freeze-thaw cycles using liquid nitrogen and a water bath (40°C) followed by extrusion 19 times through a polycarbonate membrane (pore size 200 nm). Extrusion was performed at 50°C in the case of DPPC lipids. Extra-vesicular components were removed by size exclusion chromatography on a Sephadex G-25 column with 100 mM NaCl, 10 mM HEPES, pH 7.0. Final conditions: LUVs (2.5 mM lipid); inside 100 mM NaCl, 10 mM HEPES, 1 mM 8-Hydroxypyrene-1,3,6-trisulfonic acid trisodium salt (HPTS), pH 7.0; outside: 100 mM NaCl, 10 mM HEPES, pH 7.0. Vesicles for the sodium gluconate assay were prepared by the same procedure, substituting NaCl for NaGluconate in the buffer solution.

6.2 The HPTS Assay

In a typical experiment, the LUVs containing HPTS (25 µL, final lipid concentration 31 µM) were added to buffer (1950 µL of 100 mM NaCl, 10 mM HEPES, pH 7.0) at 25°C under gentle stirring. A pulse of NaOH (20 µL, 0.5 M – final concentration 5 mM) was added at 40 secs to initiate the experiment. At 90 s the test transporter (various concentrations, typically in 5 µL DMSO) was added, followed by detergent (25 µL of Triton X100 in 7:1 (v/v) H_2O-DMSO) at 300 secs to lyse the vesicles for calibration. The fluorescence emission was monitored at λ_{em} = 510 nm (λ_{ex} = 460/405 nm). For transporter **2**, a Hill plot was fitted to 12 data points spanning the required concentration range, and each individual concentration was repeated at-least twice and averaged. The fractional fluorescence intensity (I_{rel}) was calculated using Equation S1, where R_t is the fluorescence ratio at time t, R_0 is the fluorescence ratio at time 0, and R_d is the fluorescence ratio after the addition of detergent.

$$I_{\mathrm{rel}} = \frac{R_t - R_0}{R_d - R_0} \qquad \text{(S1)}$$

The fractional fluorescence intensity (I_{rel}) at 276 s just prior to lysis, defined as the fractional activity y, was plotted as a function of the transporter concentration (x / mol%). Hill coefficients (n) and EC_{50} values were calculated by fitting to the Hill equation (S2)

$$y = y_0 + (y_{max} - y0\,\frac{x^n}{EC_{50}^n + x^n} \qquad \text{(S2)}$$

where y_0 is the fractional activity in the absence of transporter, y_{max} is the fractional activity with excess transporter, x is the transporter concentration in the cuvette.

The k_{ini} values were calculated by fitting the transport kinetics curves (I_{rel} vs t) with the exponential function I_{rel} = a – exp(-t/b) using Origin 2025. k_{ini} (the initial rate of transport immediately after the base pulse) is then given by differentiation as k_{ini} = 1/b (s^{-1}).

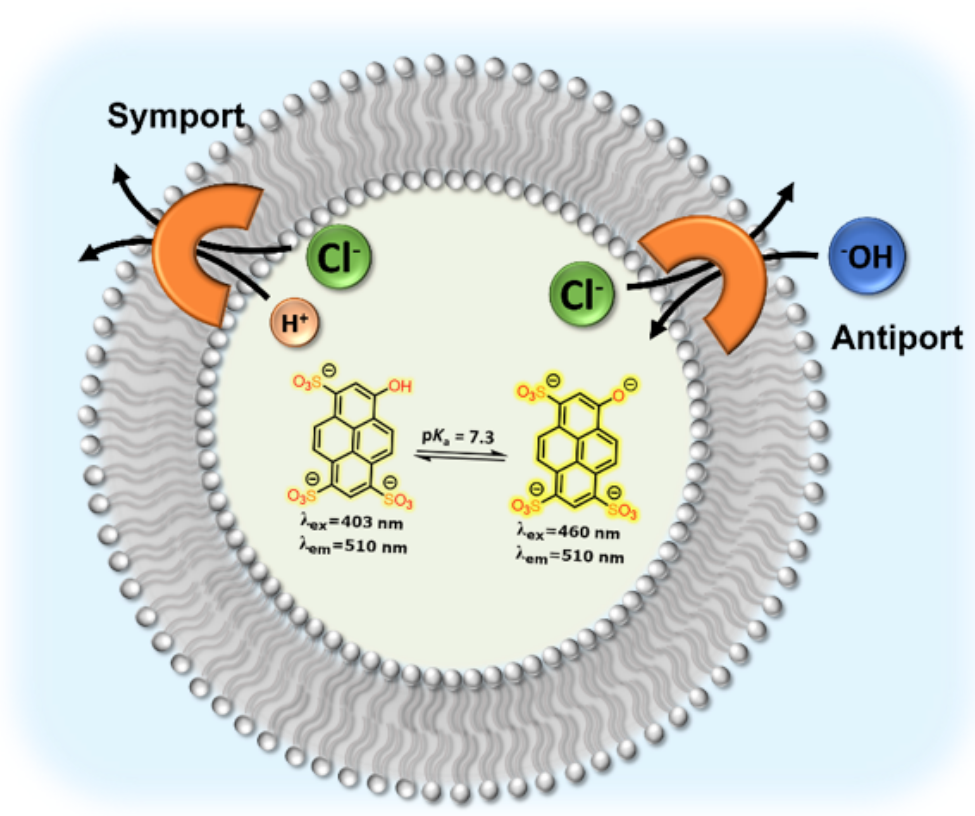

Figure S44: POPC vesicles encapsulating HPTS probe to monitor ion transport across the lipid membrane

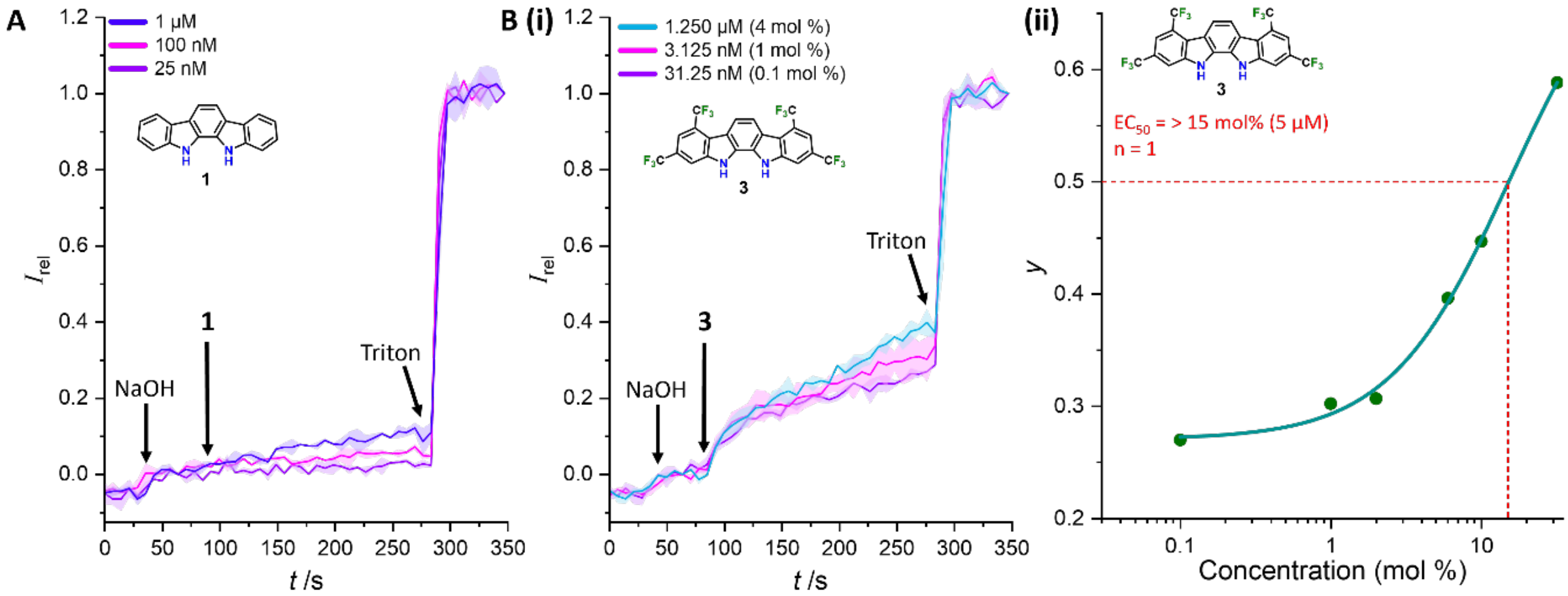

Figure S45: Change in ratiometric emission, I_{rel} (λ_{em} = 510 nm; λ_{ex1} = 405 nm, λ_{ex2} = 460 nm) with increasing concentration of ion transporter **1 (A)** and **3 (B i)**, upon addition of a NaOH base pulse (5 mM) to POPC LUVs (31 μM) containing 1 mM HPTS, 100 mM internal and external NaCl, buffered with 10 mM HEPES at pH 7.0. **B (ii)** Dependence on fractional activities (*y*, the relative intensity at *t* = 276 s immediately prior to lysis) on concentration of **3**, (●), and fitted to the Hill equation (▬)

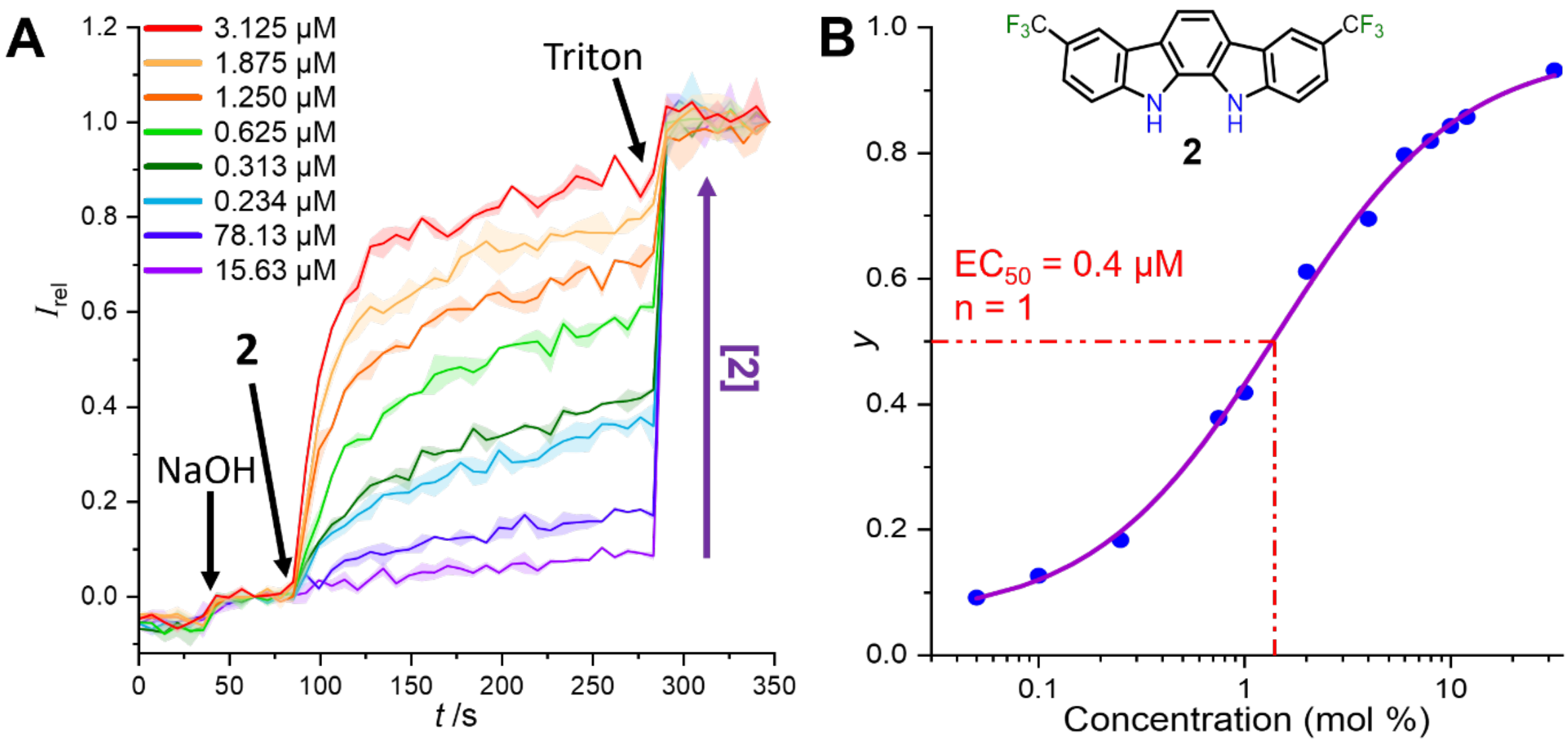

Figure S46: LEFT Change in ratiometric emission, I_{rel} (λ_{em} = 510 nm; λ_{ex1} = 405 nm, λ_{ex2} = 460 nm) with increasing concentration of ion transporter **2**, upon addition of a NaOH base pulse (5 mM) to POPC LUVs (31 μM) containing 1 mM HPTS, 100 mM internal and external NaCl, buffered with 10 mM HEPES at pH 7.0. **RIGHT** Dependence on fractional activities (*y*, the relative intensity at *t* = 276 s immediately prior to lysis) on concentration of **2**, (●), and fitted to the Hill equation (▬)

6.3 Sodium Gluconate HPTS Assay

POPC LUVs were prepared with an internal solution of 100 mM sodium gluconate, 10 mM HEPES, 1 mM HPTS buffered to pH 7.0 and an external buffer of 100 mM sodium gluconate, 10 mM HEPES, pH 7.0. This assay was performed with **2** at concentration of 0.625 µM (2 mol% transporter with respect to lipid) and repeated at least twice.

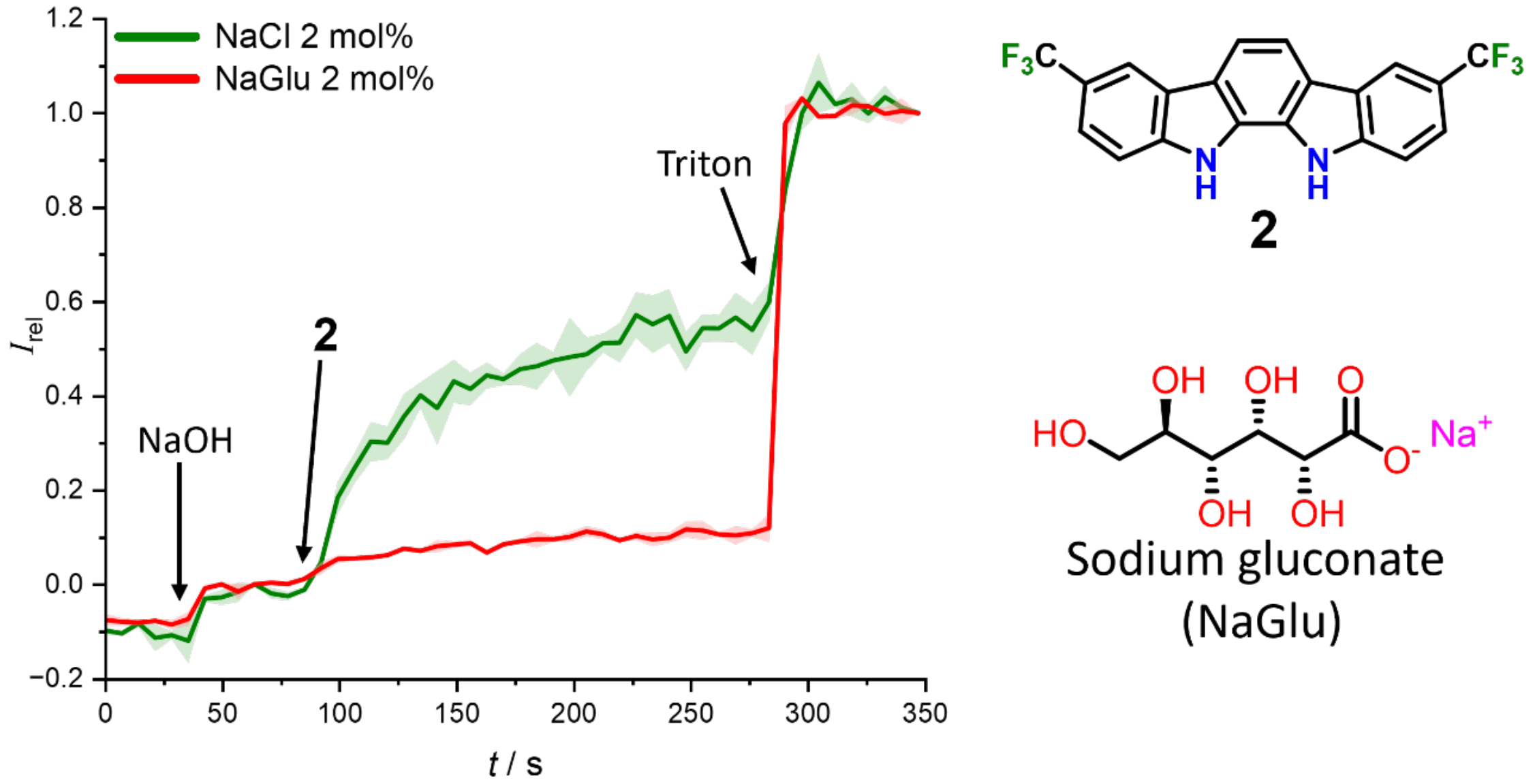

Figure S47: LEFT Ion transport HPTS assay data transporter **2** (2 mol% w.r.t lipid) in POPC LUVs with sodium gluconate (NaGlu) buffer (red line), with comparison of ion transport activity at the same mol% concentration with sodium chloride (NaCl) buffer.

6.4 HPTS Assay with DPPC Lipids

The HPTS assay was carried out using the same procedure as described above, however, the POPC LUVs were replaced with dipalmitoyl phosphatidylcholine (DPPC) LUVs. These lipids are extruded at 50 °C. Experiments with DPPC lipids were conducted in the same way as described above. For elevated temperature studies, the buffer was equilibrated at 45 °C for 5 minutes prior to initiating the experiment.

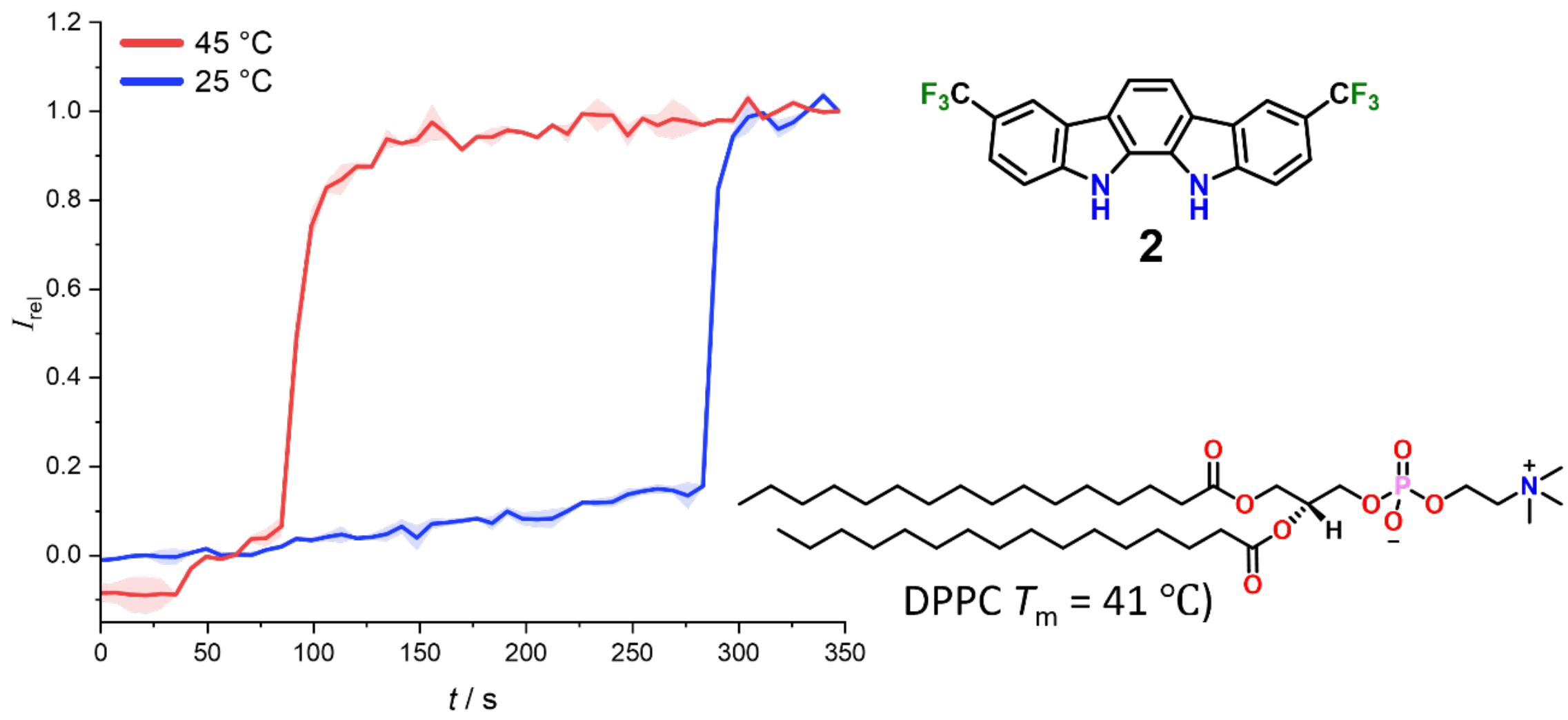

Figure S48: Ion transport HPTS assay data for transporter **2** (8 mol% w.r.t lipid) in DPPC LUVs at 25 °C and 45 °C

6.5 HPTS Assay with FCCP

Experiments in the presence of protonophore trifluoromethoxy carbonylcyanide phenylhydrazone (FCCP) were carried out using the above procedure, except that a DMSO solution of FCCP (5 µL of 100 µM solution, final concentration 0.25 µM/0.8 mol%) was added to the vesicle suspension prior to the addition of the NaOH pulse, but after the irradiation step. At this concentration, FCCP does not cause appreciable dissipation of the transmembrane pH gradient alone.

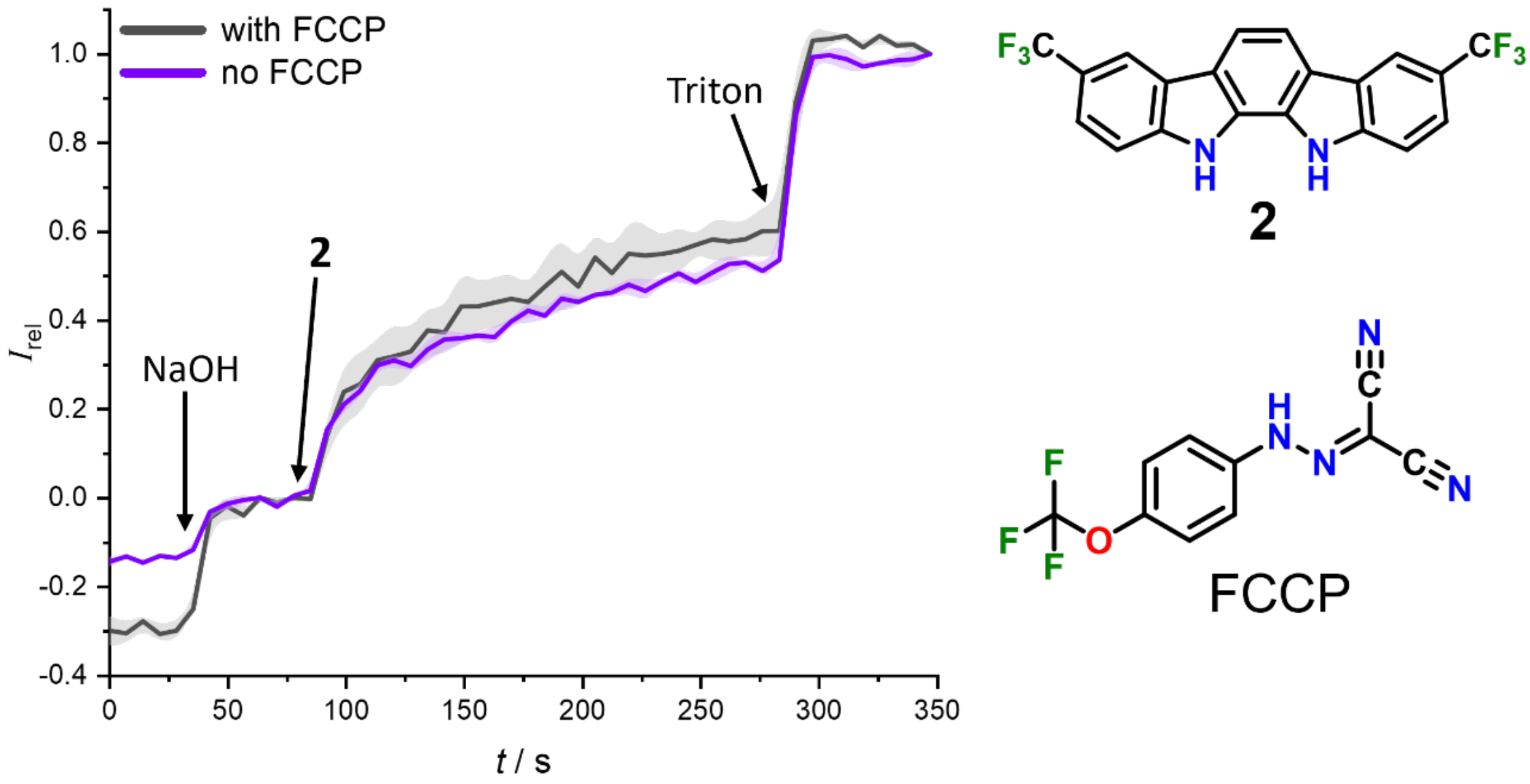

Figure S49: Ion transport HPTS assay data for transporter **2** (8 mol% w.r.t lipid) in POPC LUVs with 100 mM NaCl in 10 mM HEPES buffer in the presence and absence of FCCP (5 µL of 100 µM solution in DMSO) added prior to start of the experiment.

6.6 Photo-triggered HPTS Transport Assay

The HPTS assay was modified to allow for *in situ* irradiation of the photocaged transporters, and was carried out as follows: the desired concentration of the photocaged transporters in DMSO (5 µL) was added to the lipid suspension (POPC LUVs containing 1 mM HPTS, 100 mM internal and external NaCl, buffered with 10 mM HEPES at pH 7.0) and stirred for 1 minute to allow for thorough distribution. The sample was then irradiated with a Thorlabs mounted LED in a Thorlabs cuvette holder (CVH100/MM). The LEDs were supplied with 1 A current using Thorlabs T-cub LED driver (LEDD1B). The transport assay was initiated with a base pulse of NaOH (20 µL, 0.5 M) and the change in ratiometric emission (λ_{em} = 510 nm; λ_{ex} = 460/405 nm) was monitored over time, before calibration with Triton-X100. The photo-triggered transport activity was monitored with increasing irradiation times.

Photo-triggered transport assays were also conducted by *ex situ* irradiation of a vial containing a stock solution of the photocaged transporters in a DMSO solution. The irradiated sample in DMSO (5 µL) was added to the lipid suspension at 90 s as per the typical HPTS assay as described in Section 6.2.

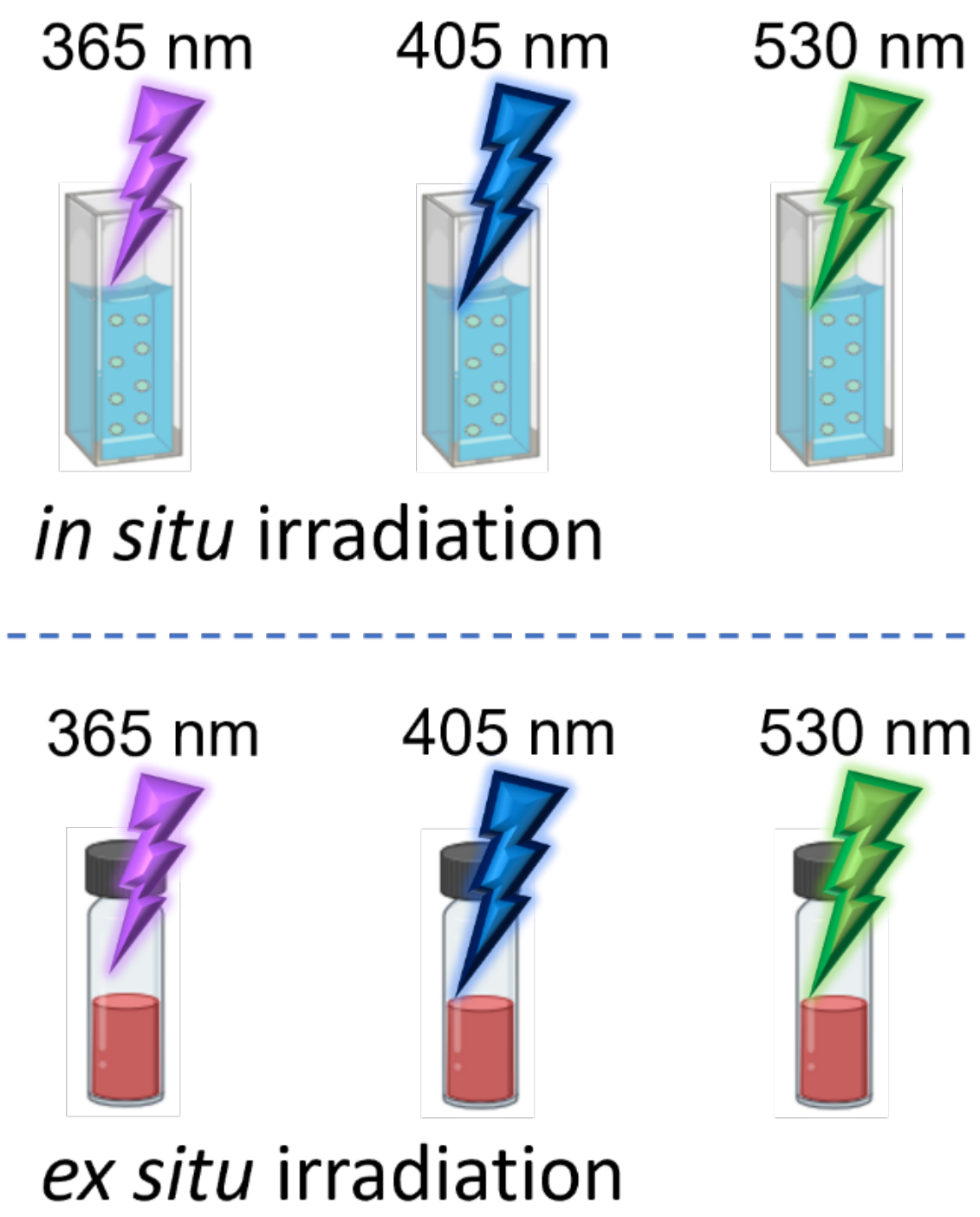

Figure S50: Schematic depiction of *in situ* irradiation in a cuvette containing the photocaged transporter and POPC LUVs in buffer (top) and *ex situ* irradiation of a vial containing stock solution of photocaged transporters in DMSO (bottom), at different wavelengths.

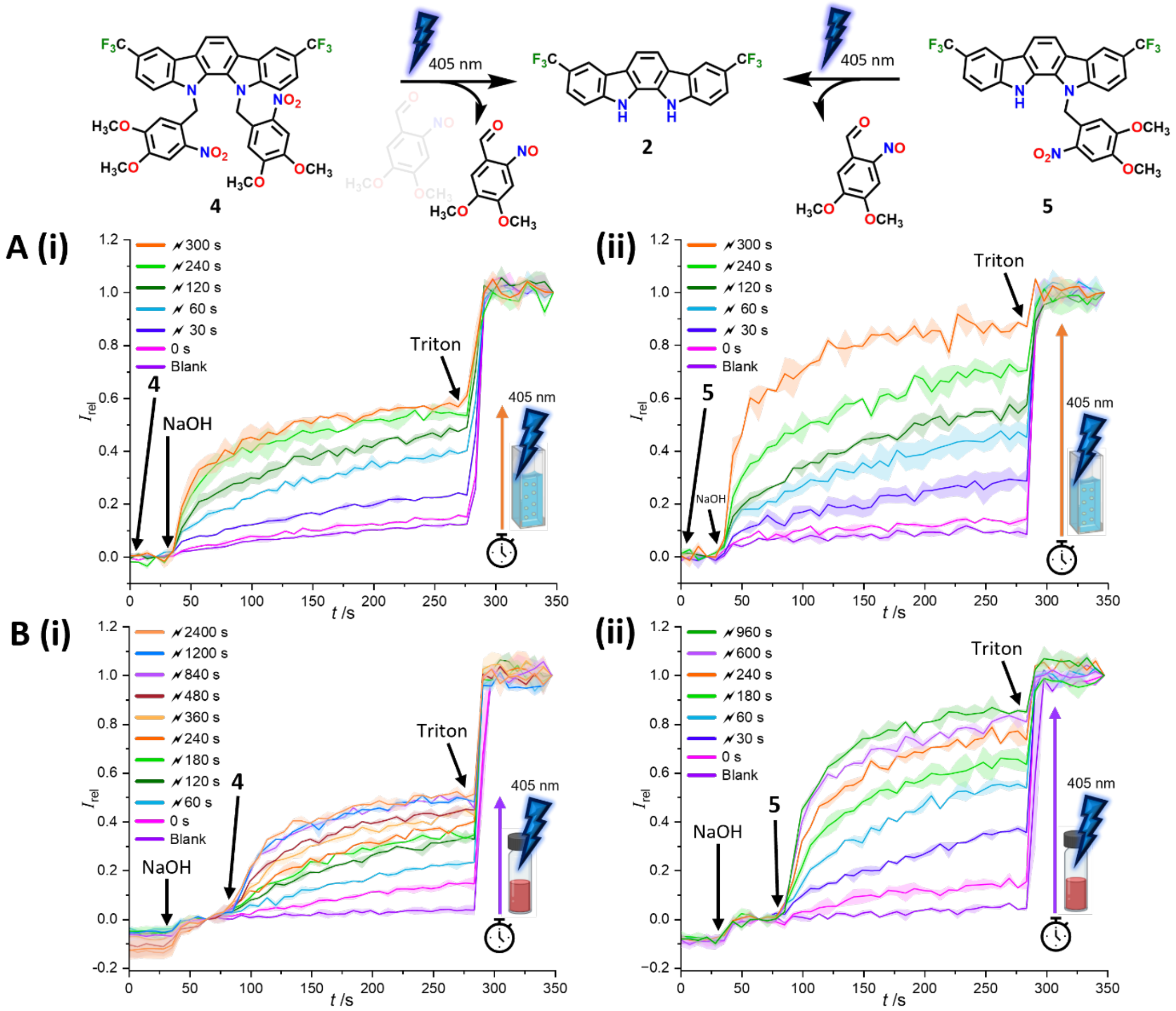

Figure S51: Change in ratiometric emission, I_{rel} (λ_{em} = 510 nm; λ_{ex1} = 405 nm, λ_{ex2} = 460 nm) with increasing irradiation time of photo-caged transporters *in situ* **(A)** and *ex situ* **(B)** with a 405 nm LED (1W), upon addition of a NaOH base pulse (5 mM) to POPC LUVs (31 µM) containing 1 mM HPTS, 100 mM internal and external NaCl, buffered with 10 mM HEPES at pH 7.0 of **(i)** double- (**4**), and **(ii)** mono- (**5**), photocaged bis(trifluoromethyl)indolo[2,3-α]carbazoles.

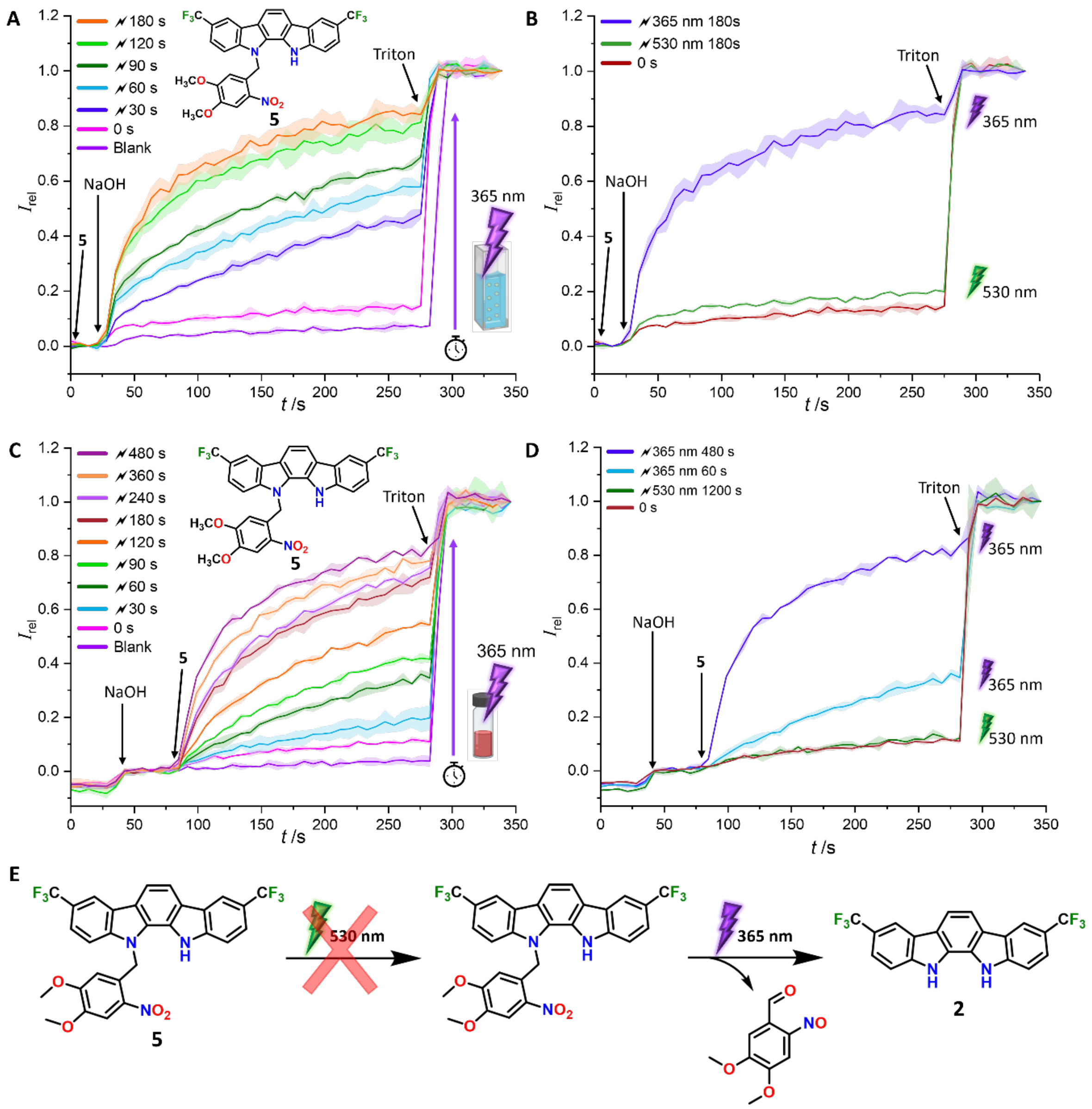

Figure S52: Change in ratiometric emission, I_{rel} (λ_{em} = 510 nm; λ_{ex1} = 405 nm, λ_{ex2} = 460 nm) with increasing irradiation time of mono-photocaged transporter **5** *in situ* **(A)** and *ex situ* **(C)** with a 365 nm LED (880 mW), upon addition of a NaOH base pulse (5 mM) to POPC LUVs (31 µM) containing 1 mM HPTS, 100 mM internal and external NaCl, buffered with 10 mM HEPES at pH 7.0. **(B)** *in situ* and **(C)** *ex situ* control assays of **5** after irradiation with a 530 nm LED (370 mW) showing no activation of transport. Irradiation for the same time with a 365 nm LED shows significant ion transport. **(E)** Photo-decaging scheme showing cleavage only occurring with irradiation at 365 nm.

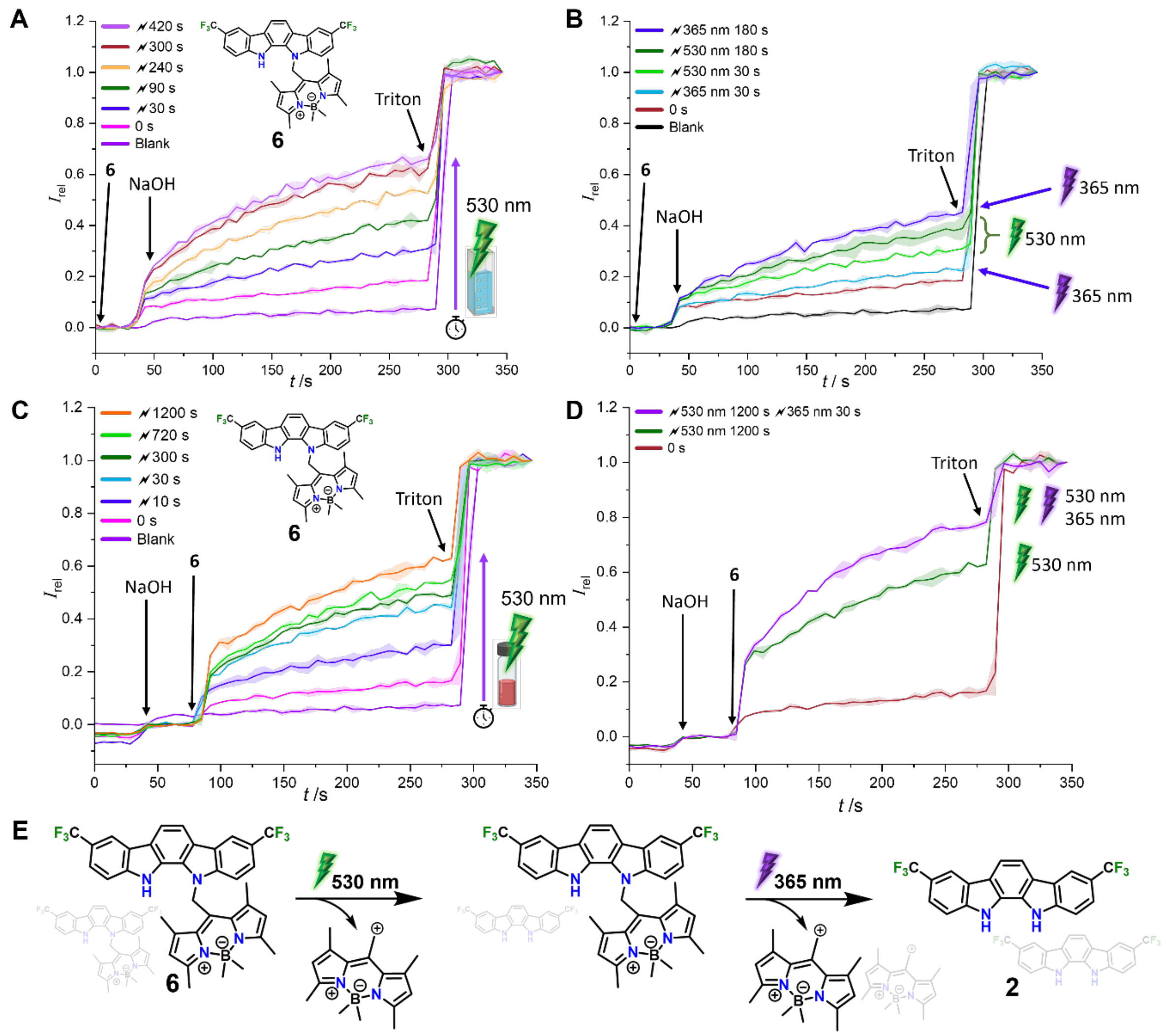

Figure S53: Change in ratiometric emission, I_{rel} (λ_{em} = 510 nm; λ_{ex1} = 405 nm, λ_{ex2} = 460 nm) with increasing irradiation time of mono-photocaged transporter **6** *in situ* **(A)** and *ex situ* **(C)** with a 530 nm LED (370 mW), upon addition of a NaOH base pulse (5 mM) to POPC LUVs (31 µM) containing 1 mM HPTS, 100 mM internal and external NaCl, buffered with 10 mM HEPES at pH 7.0. **(B)** *in situ* and **(C)** *ex situ* control assays of **6** after irradiation with a 365 nm LED (880 mW) showing at shorter wavelengths there is no selective cleavage, and transport is activated. **(E)** Photo-decaging scheme showing cleavage occurring with irradiation at 530 nm or 365 nm.

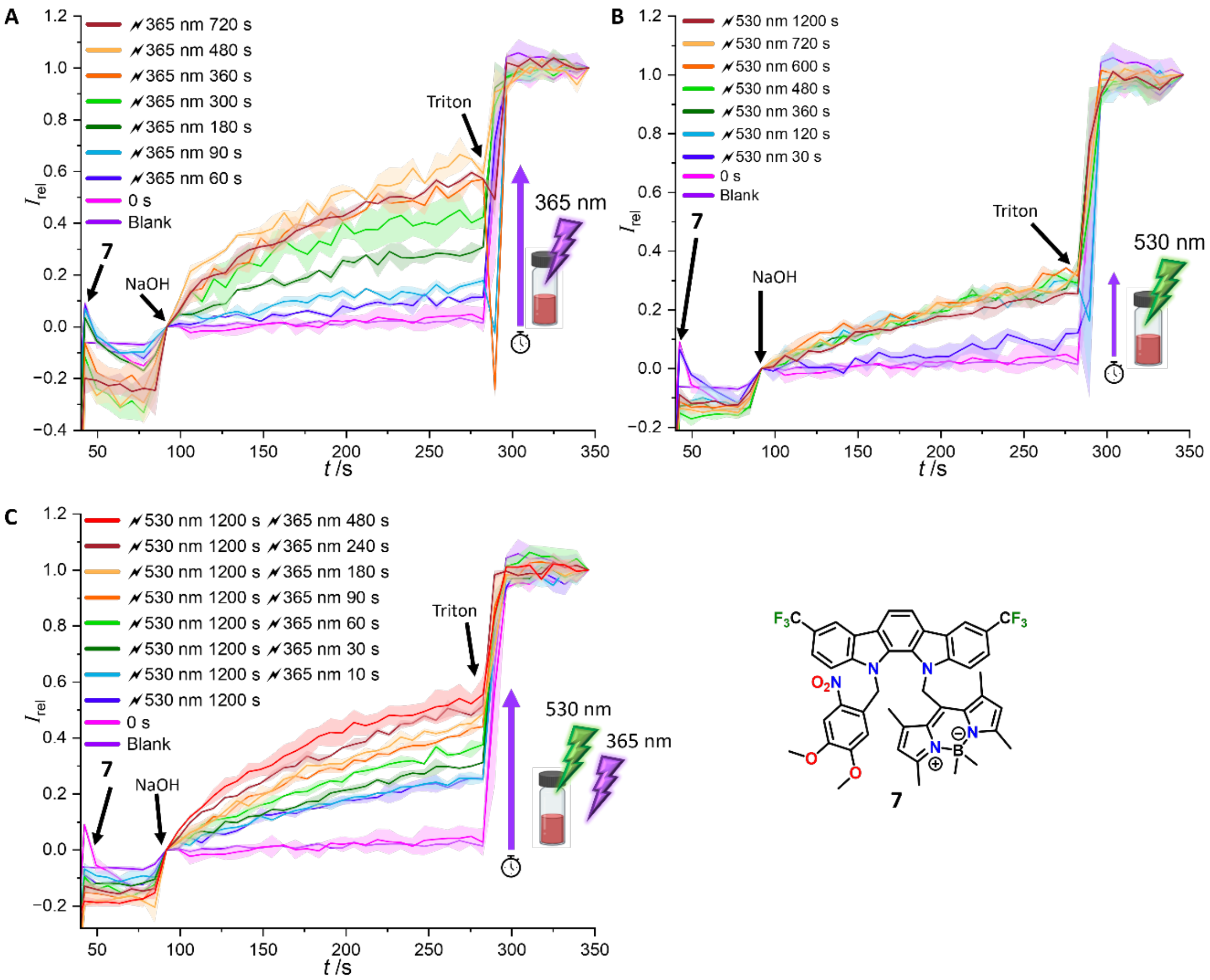

Figure S54: Change in ratiometric emission, I_{rel} (λ_{em} = 510 nm; λ_{ex1} = 405 nm, λ_{ex2} = 460 nm) with increasing *ex situ* irradiation time of dual-photocaged transporter **7** upon addition of a NaOH base pulse (5 mM) to POPC LUVs (31 µM) containing 1 mM HPTS, 100 mM internal and external NaCl, buffered with 10 mM HEPES at pH 7.0. Irradiation of **7** with **(A)** a 365 nm LED (880 mW), **(B)** a 530 nm LED (370 mW), **(C)** sequential irradiation with a 530 nm LED, followed by irradiation with a 365 nm LED.

6.7 Photo-triggered HPTS Transport Assay in Designated Vesicle Populations

Photo-triggered activation of chloride transport in designated vesicle populations was conducted using the HPTS assay. The lipid suspension (POPC LUVs containing 16 mM HPTS, 100 mM internal and external NaCl, buffered with 10 mM HEPES at pH 7.0) was divided into two cuvettes. In each cuvette, either photocaged transporter **6** (Population A), or photocaged transporter **5** (Population B), in DMSO (1.25 µM or 4 mol% w.r.t lipid) was added to the lipid suspension and stirred for 1 minute to allow for thorough distribution. Population B was then added to Population A, forming a 50:50 mixture of populations A and B (total photocaged transporter concentration 2.5 µM or 8 mol% w.r.t lipid), and stirred for a further minute. The sample was then irradiated (*in situ*) with a Thorlabs mounted LED in a Thorlabs cuvette holder (CVH100/MM). The LEDs were supplied with 1 A current using Thorlabs T-cub LED driver (LEDD1B). The transport assay was initiated with a base pulse of NaOH (20 µL, 0.5 M) and the change in ratiometric emission (λ_{em} = 510 nm; λ_{ex} = 460/405 nm) was monitored over time, before calibration with Triton-X100. The photo-triggered transport activity was monitored with increasing irradiation times with either, or both, 365 nm LED (880 mW) and 530 nm LED (370 mW).

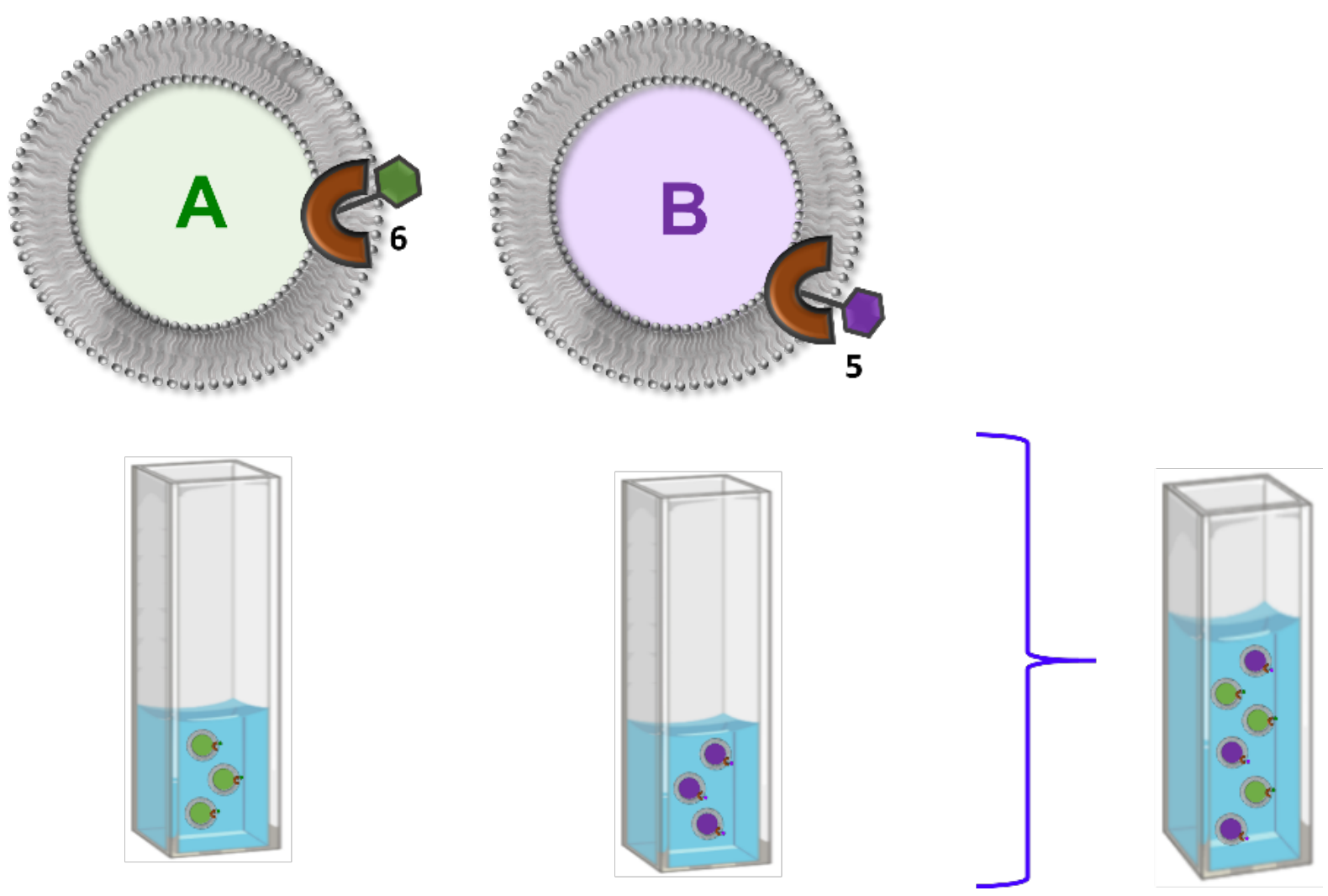

Figure S55: Schematic representation of the 50:50 vesicle populations A and B preparation. Vesicle population A contains photocaged transporter **6** (4 mol% w.r.t lipid), and population B contains photocaged transporter **5** (4 mol% w.r.t lipid). For the assay, equal amounts of populations A and B are combined to form a 50:50 mixture of the two vesicle populations.

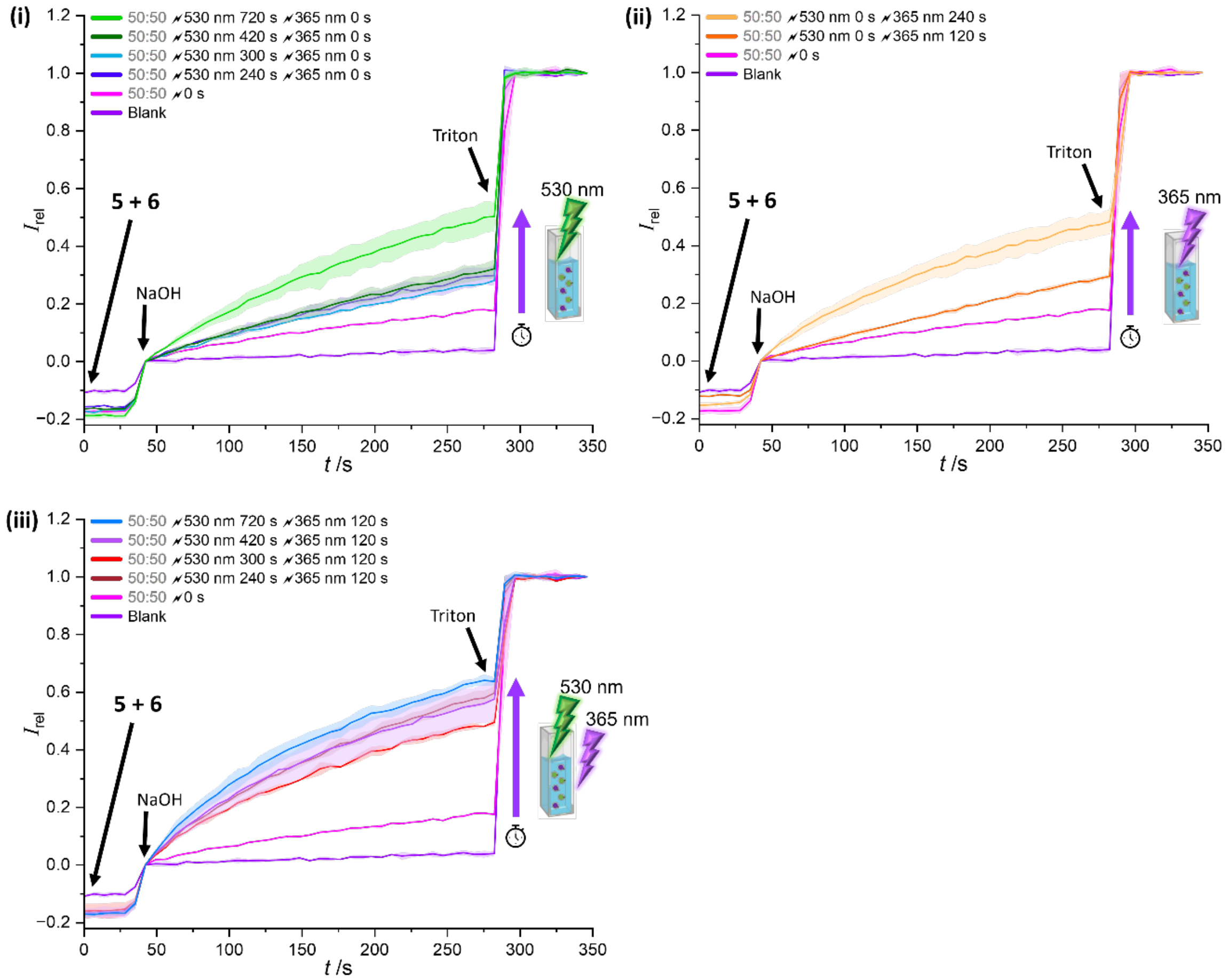

Figure S56: Change in ratiometric emission, I_{rel} (λ_{em} = 510 nm; λ_{ex1} = 405 nm, λ_{ex2} = 460 nm) with increasing *in situ* irradiation time of 50:50 vesicle populations A (with photocaged transporter **6**) and population B (with photocaged transporter **5**), upon addition of a NaOH base pulse (5 mM) to POPC LUVs (31 µM) containing 16 mM HPTS, 100 mM internal and external NaCl, buffered with 10 mM HEPES at pH 7.0. Irradiation of A:B populations with **(i)** a 530 nm LED (370 mW), **(ii)** a 365 nm LED (880 mW **(C)** sequential irradiation with a 530 nm LED, followed by irradiation with a 365 nm LED.

A control assay was conducted to investigate the potential of the transporter migrating from one vesicle population to another. Two different vesicle populations were prepared – Population C was prepared from a lipid suspension containing pre-incorporated transporter **2** (1 mol% w.r.t lipid) (POPC LUVs, 100 mM internal and external NaCl, buffered with 10 mM HEPES at pH 7.0); Population D was prepared as a lipid suspension (POPC LUVs containing 1 mM HPTS, 100 mM internal and external NaCl, buffered with 10 mM HEPES at pH 7.0). The transport assay was initiated with a base pulse of NaOH (20 µL, 0.5 M) and the change in ratiometric emission (λ_{em} = 510 nm; λ_{ex} = 460/405 nm) was monitored over time, before calibration with Triton-X100 (Fig. S57).

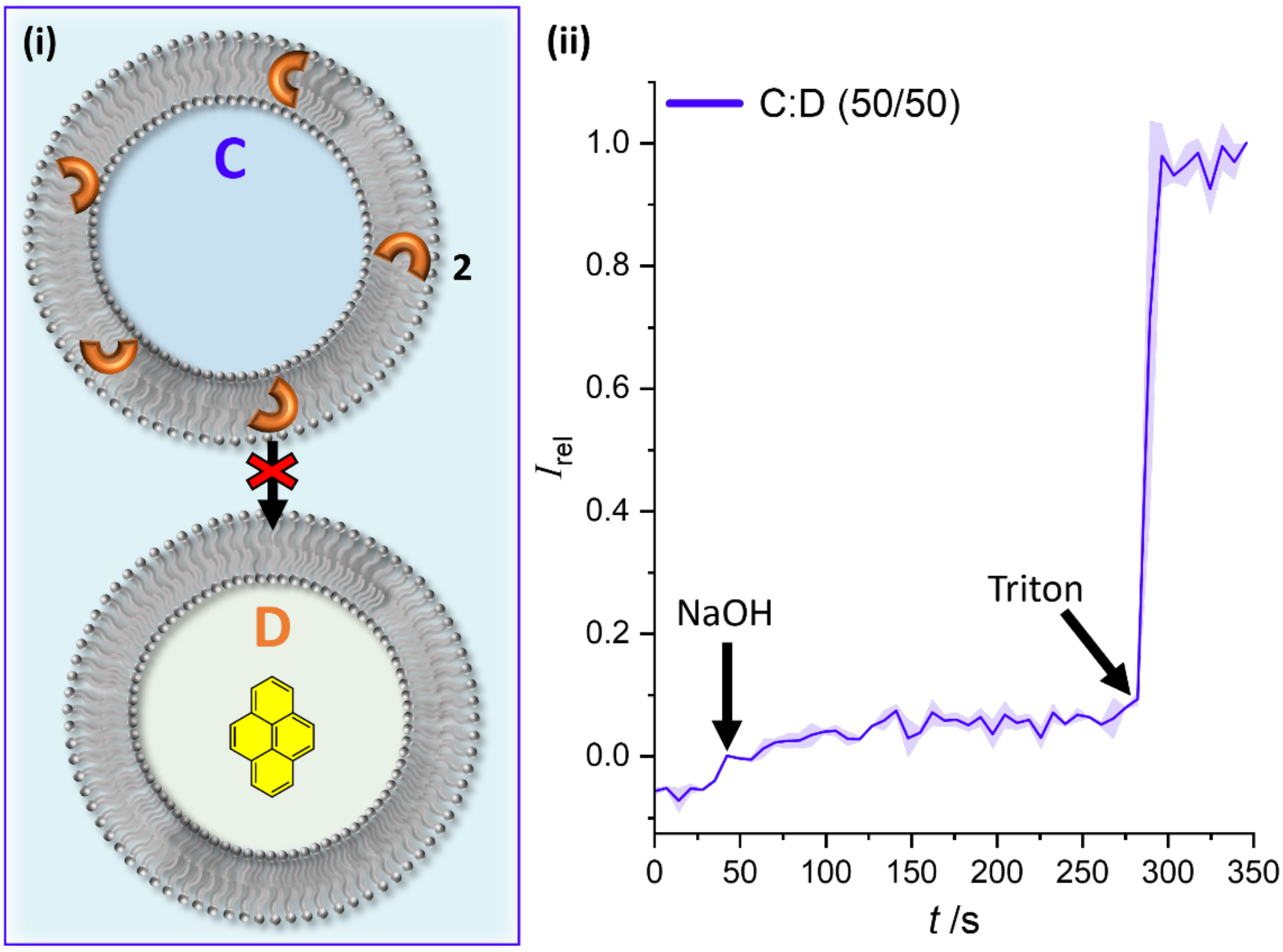

Figure S57(i): Schematic representation of the control assay to investigate ionophore transfer between two vesicle populations. Vesicle population **C** contains uncaged transporter **2** pre-incorporated in the bilayer membrane; vesicle population **D** contains internal HPTS fluorophore **(ii)** Change in ratiometric emission, I_{rel} (λ_{em} = 510 nm; λ_{ex1} = 405 nm, λ_{ex2} = 460 nm) upon addition of a NaOH base pulse (5 mM) to a cuvette containing 50:50 mixture of vesicle populations **C** and **D**. Vesicle population **C** contains 1 mol% (w.r.t lipid) of uncaged transporter **2** pre-incorporated in the bilayer membrane, and population **D** encapsulates 1 mM HPTS. Conditions for both **C** and **D**: POPC LUVs (31 µM) containing 100 mM internal and external NaCl, buffered with 10 mM HEPES at pH 7.0.

7 References

1. A. Brown, K. M. Mullen, J. Ryu, M. J. Chmielewski, S. M. Santos, V. Felix, A. L. Thompson, J. E. Warren, S. I. Pascu and P. D. Beer, *J. Am. Chem. Soc.*, 2009, **131**, 4937–4952.
2. D. Curiel, A. Cowley and P. D. Beer, *Chem. Commun.*, 2005, **2**, 236–238.
3. Y.-Z. Hu and Y.-Q. Chen, *Synlett*, 2004, **1**, 42–48.
4. P. Wu, H. He, H. Ma, B. Tu, J. Li, S. Guo, S. Chen, N. Cao, W. Zheng, X. Tang, D. Li, X. Xu, X. Zheng, Z. Sheng, W. David Hong and K. Zhang, *Bioorg. Chem.*, 2021, **107**, 104580.
5. K. K. Behara, Y. Rajesh, Y. Venkatesh, B. R. Pinninti, M. Mandal and N. D. P. Singh, *Chem. Commun.*, 2017, **53**, 9470–9473.
6. M. Wilcox, R. W. Viola, K. W. Johnson, A. P. Billington, B. K. Carpenter, J. A. McCray, A. P. Guzikowski and G. P. Hess, *J. Org. Chem.*, 1990, **55**, 1585–1589.
7. K. Sitkowska, B. L. Feringa and W. Szymański, *J. Org. Chem.*, 2018, **83**, 1819–1827.
8. P. Shrestha, A. Mukhopadhyay, K. C. Dissanayake and A. H. Winter, *J. Org. Chem.*, 2022, **87**, 14334–14341.
9. J. A. Peterson, L. J. Fischer, E. J. Gehrmann, P. Shrestha, D. Yuan, C. S. Wijesooriya, E. A. Smith and A. H. Winter, *J. Org. Chem.*, 2020, **85**, 5712–5717.
10. K. Krumova and G. Cosa, *J. Am. Chem. Soc.*, 2010, **132**, 17560–17569.
11. D. O. Soloviev and C. A. Hunter, *Chem. Sci.*, 2024, **15**, 15299–15310.
12. D. Brynn Hibbert and P. Thordarson, *Chem. Commun.*, 2016, **52**, 12792–12805.
13. A. Daina, O. Michielin and V. Zoete, *Scientific Reports*, 2017, **7**, 42717.
14. BindFit v0.5 | Supramolecular, http://app.supramolecular.org/bindfit/, (accessed 20 June 2023).
15. A. Kerckhoffs and M. J. Langton, *Chem Sci*, 2020, **11**, 6325–6331.
